# A pH-responsive double network hydrogel for control of tomato bacterial wilt

**DOI:** 10.1038/s41467-026-73922-3

**Published:** 2026-06-12

**Authors:** Shunyu Xiang, Meijun Chen, Shaorui Tian, Haoran Peng, Hulian Sun, Mengji Cao, Cécilia Ménard-Moyon, Xianchao Sun, Alberto Bianco

**Affiliations:** 1 CNRS, Immunology, Immunopathology and Therapeutic Chemistry, UPR 3572, University of Strasbourg, ISIS, Strasbourg, France; 2 College of Plant Protection, National Citrus Engineering Research Center, Citrus Research Institute, Southwest University, Chongqing, China; 3 University of Strasbourg Institute of Advanced Study (USIAS), Strasbourg, France

**Keywords:** Nanobiotechnology, Nanostructures, Plant biotechnology

## Abstract

*Ralstonia solanacearum* is a major plant pathogen causing bacterial wilt, whose unpredictable onset hinders timely detection and effective control. Here, we report the design, preparation and field use of a dual pH-responsive multifunctional double network (DN) hydrogel for the efficient and sustainable control of bacterial wilt. The primary network of carboxylated agarose chelates Zn^2+^ and loosens under acidic conditions (pH ≤ 5) to release a pesticide (zhongshengmycin) and Zn^2+^, while the secondary L-phenylalanine (Phe)/ Zn^2+^ network disassembles to provide additional bioactive components (Phe and Zn^2+^). This dual-triggered release achieves a combined antibacterial effect, enhances plant growth, and activates plant disease resistance pathways. A simple root application protects plants for up to 14 days, and field experiments demonstrate disease control for up to 30 days, significantly preserving tomato yield. Here, we present a sustainable, effective system for managing bacterial wilt and highlight the potential of smart hydrogels in crop protection.

## Abstract (web summary)

Bacterial wilts unpredictable onset makes control difficult. Here, the authors report on a dual pH-responsive hydrogel system which releases antimicrobial agents and plant immune elicitors, L-phenylalanine and zinc, in acidic soils to enable control of bacterial wilt.

## Introduction

Bacterial wilt, caused by *Ralstonia solanacearum* (*R. solanacearum*), is a highly destructive soil-borne disease that severely impacts over 200 plant species across more than 40 families^1,2^. Notable examples include *Solanaceae* crops like tomatoes, potatoes, peppers, ginger, and eggplants^3,4^. The disease is reported to have an infection rate exceeding 50% in many regions worldwide^3^, with peppers being especially vulnerable, suffering a 100% infection degree^1^. Even more concerning is the irreversible nature of the disease once it is established, as no effective treatment exists to halt the plant’s inevitable demise. Consequently, bacterial wilt can result in crop yield reductions ranging from 15% to 55%, with losses as high as 88% in tomatoes and 75% in potatoes^5,6^. Currently, bacterial wilt is considered one of the most serious threats to global agricultural production, affecting over 1.7 million acres across more than 80 countries and causing annual economic losses exceeding one billion dollars^7,8^. Despite continuous research and efforts to improve control measures, their effectiveness remains limited^3,6^. The overuse of agricultural antibiotics, often seen as a solution, not only promotes pathogen resistance but also presents serious environmental risks^9^. These antibiotics disrupt soil ecosystems, favor harmful microorganisms, and potentially exacerbate the disease^9,10^. Accordingly, the development of sustainable and effective strategies for the management of bacterial wilt has been a major focus of research over the past several decades.

Recent studies have established a strong correlation between bacterial wilt and the physicochemical properties of soil^11^. For example, the proliferation of *R. solanacearum* in the soil may produce organic acids that could contribute to soil acidification^12,13^. Additionally, external factors such as acid rain and improper fertilization further exacerbate this acidification, thereby worsening the disease^14^. Field investigations consistently show that soils from fields with high incidences or severe outbreaks of bacterial wilt have significantly lower pH levels than those in fields with mild or no disease^11^. Laboratory studies confirmed that lowering soil pH to 4.5 increases both the occurrence and severity of bacterial wilt^11^. To mitigate this problem, researchers have explored soil amendments such as calcium carbonate, oyster shell powder, wood ash, and lime to raise soil pH^11,12,15^. While these methods have demonstrated moderate success in reducing disease incidence, their effectiveness is diminished in heavily colonized soils, as *R. solanacearum* exhibits remarkable resilience under adverse conditions^6,7,16^. Encouragingly, advancements in agricultural material science have facilitated the development of innovative pesticide formulations targeting specific diseases such as bacterial wilt^17–19^. One promising solution is pH-responsive nanocarriers, which selectively release pesticides in acidic soils^20,21^. For example, Liang et al. developed a pH-responsive core-shell metal-organic framework nanocarrier using ZnO and ZIF-8 via an in-situ growth strategy^20^. This system enabled pH-responsive release of berberine and significantly reduced the incidence and severity of tomato bacterial wilt compared to the aqueous formulation. In addition, other nanomaterials, such as mesoporous silica nanoparticles, have also been widely explored for the construction of pH-responsive pesticide delivery systems for plant disease control^22^. However, these systems are predominantly applied to foliar application^22^, and their potential for managing root-associated diseases has not been systematically investigated. Most importantly, the practical application of nanomaterials in soil is hindered by environmental variables, such as ion strength, pH, and organic matter content, which cause nanoparticle aggregation and uneven distribution, thereby reducing disease control effectiveness^23^. Therefore, the development of innovative pesticide carriers optimized for soil application is a critical priority for effectively managing soil-borne diseases like bacterial wilt.

Hydrogels, known for their adjustable mechanical properties, customizable structures (such as spherical, block-shaped, and nanoscale forms), and multi-stimulus responsiveness (to factors like pH, light, temperature, and enzymes), have become vital drug delivery carriers in a range of biomedical applications, including tumor therapy, wound healing, and bone repair^24,25^. These properties make hydrogels an attractive option for drug delivery to plant roots or soil. Several pesticide-loaded hydrogels targeting soil-borne bacteria and fungi have already been developed, demonstrating significant effectiveness in real-world applications^26–28^. Our previous research showed that pesticide-loaded hydrogels can be applied to soil for the effective control of plant virus development and infections^29,30^. To address the chronic nature of viral plant diseases, we developed a hydrogel that gradually releases antiviral agents and calcium ions, providing sustained disease resistance and long-term protection^29^. Additionally, this hydrogel supported plant growth and ensured safety for both plants and the environment. These findings highlight the potential of hydrogels as a promising solution for managing specialized plant diseases in soil-based applications.

In this study, we developed a multifunctional and dual-pH-responsive hydrogel specifically designed to combat bacterial wilt. Agarose was chosen as the primary carrier due to its high biocompatibility, excellent drug-loading capacity, biodegradability, and environmental friendliness^31,32^. To impart pH-responsiveness, carboxyl groups were introduced on the polymer through chemical modification^33,34^. The first strategy involved the introduction of carboxymethyl groups onto agarose to obtain carboxymethylated agarose (CMA). CMA can coordinate with Zn^2+^ to form a pH-responsive hydrogel. Under acidic conditions, the protonation of the carboxyl groups weakens their binding to Zn^2+^, leading to a loosening of the hydrogel structure and facilitating the release of both drugs and Zn^2+^^33,35^. Owing to this mechanism, CMA-based hydrogels have been widely applied in drug delivery systems, particularly for anticancer and antibacterial agents^33^. However, the synthesis of CMA is relatively complex and typically requires substantial amounts of organic solvents^33^, which may increase production costs and raise potential environmental concerns for agricultural applications. In addition, the relatively low density of carboxyl groups in CMA may limit its pH-responsiveness. To overcome these limitations, a second strategy was proposed by introducing citric acid, which contains multiple carboxyl groups, to modify agarose, yielding citrate agarose (CTA)^34^. Citric acid can be covalently linked to agarose via esterification under relatively mild conditions^34^, without the need for organic solvents, making the process more environmentally friendly and cost-effective. More importantly, the presence of carboxylic acids in the structure of citric acid is expected to enhance the coordination of Zn^2+^, thereby improving gel strength and pH-responsiveness. Despite these potential advantages, studies on CTA hydrogels for drug delivery remain limited, and it is still unclear whether CTA offers superior overall performance compared to CMA. Therefore, a systematic comparison between CMA- and CTA-based hydrogels is necessary to evaluate their relative performance and to determine their suitability for agricultural applications.

**Fig. 1:**
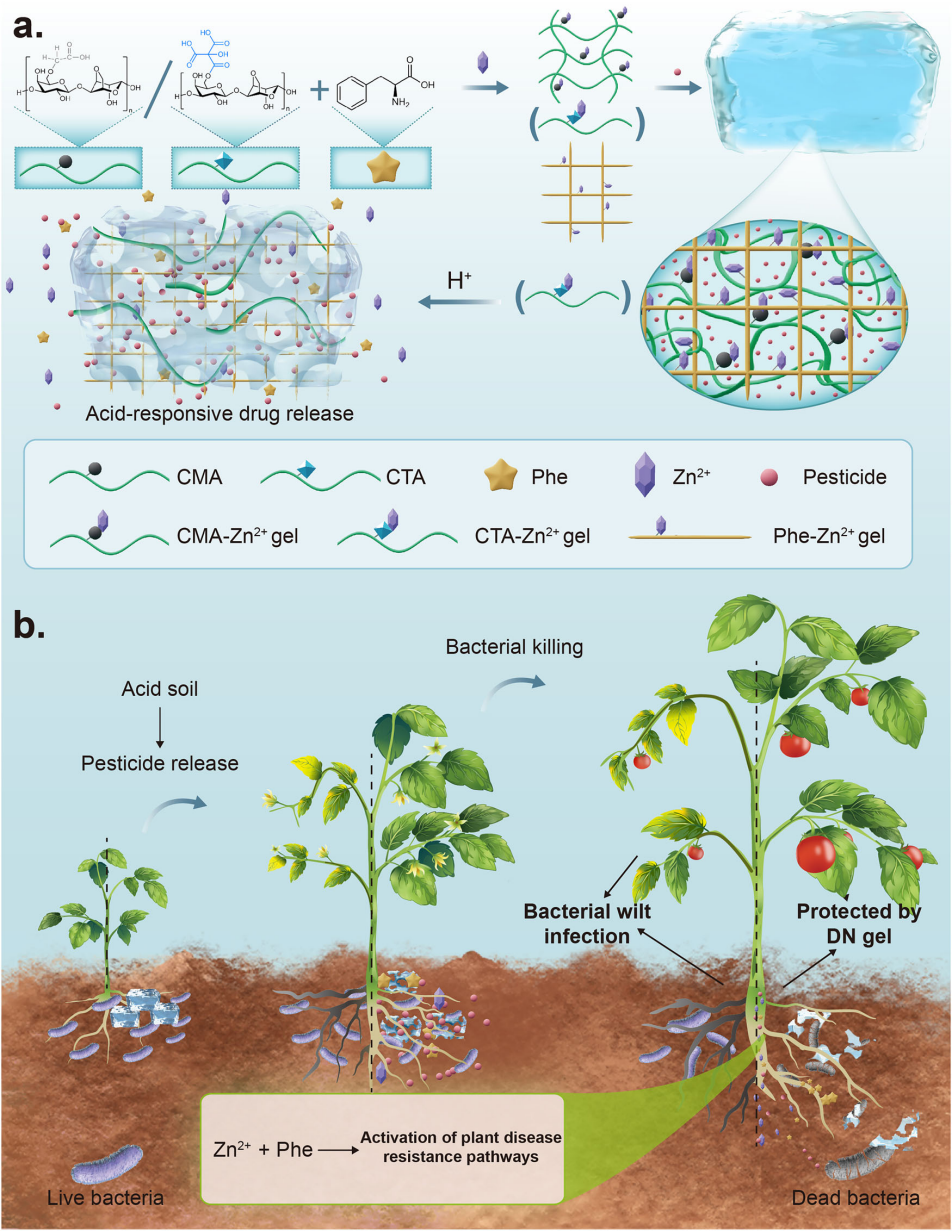
Synthesis of the CMA/CTA DN gels and mechanism of bacterial wilt control. **a** Schematic illustration of the fabrication of the CMA/CTA DN gels and their acid-responsive release of zhongshengmycin, Zn^2+^, and phenylalanine. **b** Proposed mechanism of CMA/CTA DN gels for controlling bacterial wilt through acid-triggered release of zhongshengmycin, Zn^2+^, and Phe, resulting in direct antibacterial activity and activation of systemic resistance in tomato plants.

However, agarose hydrogels alone have inherent limitations, including poor mechanical properties and uncontrolled drug release^31^. Their single-network structure often results in insufficient swelling and structural instability in complex environments like soil, causing uneven drug release and making it difficult to achieve precise pH-responsive release^32^. To overcome these limitations, we planned to introduce a second network into the CMA or CTA hydrogels. In this context, self-assembled amino acids were considered ideal building blocks, as they can function as an effective secondary network in DN systems. Their self-assembly is driven by non-covalent interactions, such as metal coordination, H-bonding and π–π stacking, leading to the formation of a dynamic, reversible, and stimuli-responsive structure. Among various amino acids and assembly strategies, L-phenylalanine (Phe), an essential amino acid for plant growth^36^, was selected. Specifically, Zn^2+^ was then employed to coordinate with Phe, enabling the in-situ formation of a Phe-Zn^2+^ supramolecular secondary network^37^. Notably, this assembly process occurs directly in aqueous conditions, avoiding the use of organic solvents (e.g., dimethylsulfoxide)^38,39^, which are commonly employed in solvent-mediated strategies to induce or regulate the self-assembly of amino acids or their derivatives, thereby ensuring improved environmental compatibility and safety. In addition, this strategy minimizes the complexity of the system, as the introduction of Zn^2+^ is sufficient to promote the formation of both the CMA/CTA network and the Phe-Zn^2+^ secondary network. More importantly, under acidic conditions, the protonation of the carboxylic group of Phe weakens its coordination with Zn^2+^, leading to the disassembly of the Phe-Zn^2+^ network. This feature further enhances the pH-responsiveness of the CMA and CTA hydrogels. Based on these advantages, in this study, the combination of the couple Phe-Zn^2+^ was ultimately selected for the formation of the secondary network. Thus, we constructed two types of dual pH-responsive DN gels by crosslinking CMA or CTA with Phe using Zn^2+^ as the crosslinking agent (Fig. 1a). Compared to single-network agarose hydrogels, the DN structure significantly enhances the structural density and mechanical properties, ensuring long-term stability in soil environments. Additionally, the pH-responsive characteristics of the CMA/CTA-Zn^2+^ and Phe-Zn^2+^ crosslinked network allowed to ameliorate the process of drug release, ensuring efficient control of bacterial wilt in acidic soils. These hydrogels, loaded with the broad-spectrum antibacterial agent zhongshengmycin (hereafter referred to as the pesticide)^6^, can release Zn^2+^ and the pesticide synergistically in acidic environments (Fig. 1a), enhancing the antibacterial activity. Moreover, Zn^2+^ and Phe act as inducers of plant immunity^40,41^, boosting the resistance to the pathogen while also serving as fertilizers to promote plant growth and ensure crop yield (Fig. 1b). In this study, we propose an innovative strategy for managing bacterial wilt that integrates precise disease control, enhanced plant immunity, and growth promotion into a multifunctional pH-responsive hydrogel system. This strategy not only ensures a better crop production but also alleviates the environmental issues caused by the overuse of chemical pesticides.

## Results

### Characterization of CMA, CTA, and CMA/CTA DN gels

**Fig. 2:**
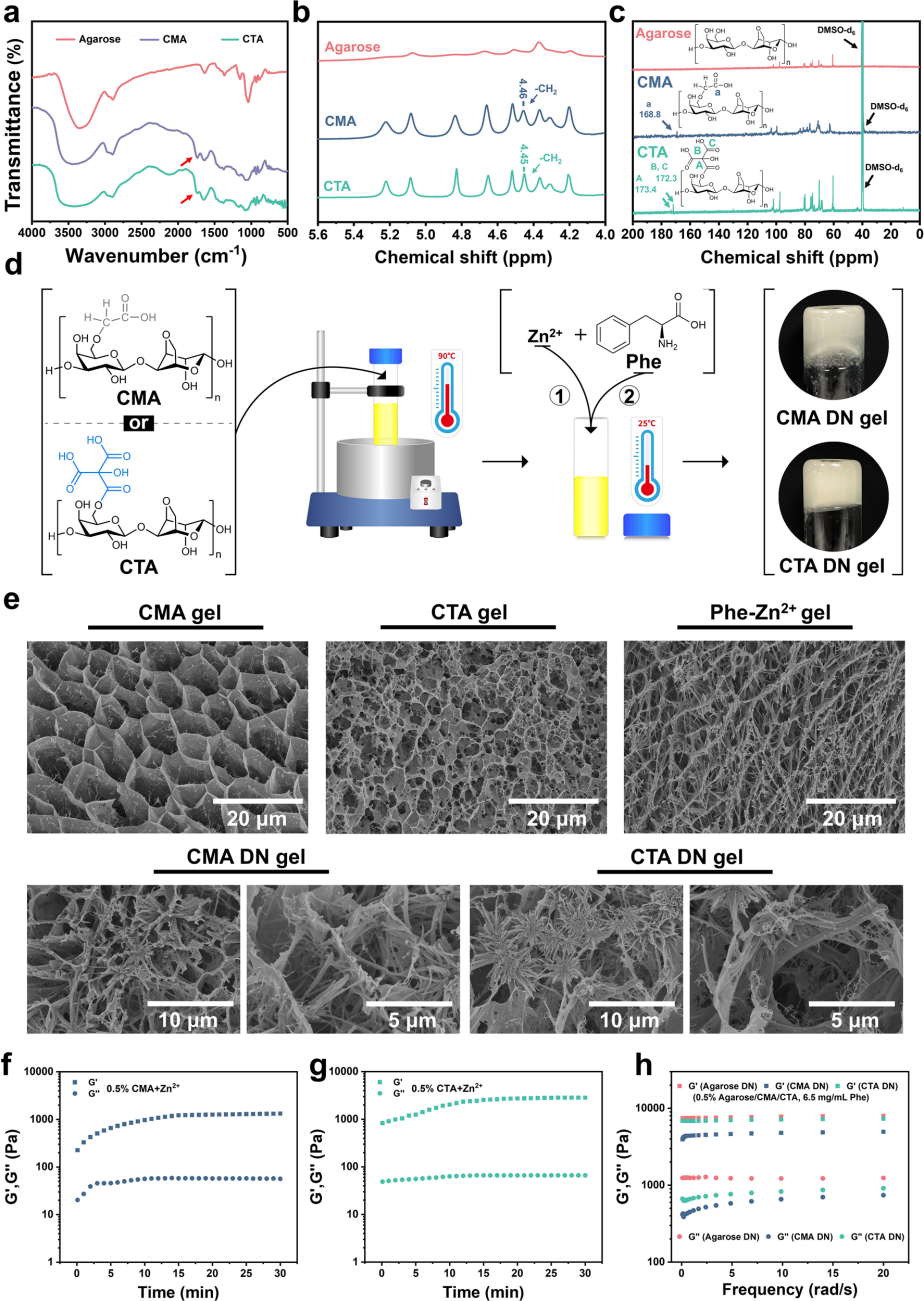
Physicochemical characterization of CMA/CTA and the corresponding DN gels. **a** FTIR spectra of agarose, CMA, and CTA. The red arrows indicate the carboxyl group peak. **b** ^1^H NMR and **c** ^13^C NMR spectra of agarose in DMSO-d_6_. **d** Schematic illustration of the preparation procedure for CMA or CTA DN gels. **e** Cryo-SEM images of CMA, CTA, Phe-Zn^2+^, CMA DN, and CTA DN gels. All observations were independently repeated 3 times to ensure the robustness of the results. **f**, **g** Rheological monitoring of gelation kinetics via time sweep analysis of storage modulus (G’) and loss modulus (G”) for **f** 0.5% CMA + Zn^2+^ and **g** 0.5% CTA + Zn^2+^. **h** Frequency sweep profiles of agarose-, CMA-, and CTA-based DN gels.

For the preparation of CMA and CTA, we adopted a simplified procedure to introduce carboxymethyl and citric acid groups onto agarose (Supplementary Fig. 1). The successful derivatization of agarose was confirmed by FTIR spectroscopy, zeta potential analysis, and NMR spectroscopy. As shown in Fig. 2a, the FTIR spectra of CMA and CTA exhibited a prominent peak around 1730 cm^−1^, which was absent in the spectrum of agarose, corresponding to the characteristic stretching vibration of C = O, indicating the successful introduction of carboxyl groups^33,34^. Additionally, FTIR analysis of CTA performed at different reaction times (Supplementary Fig. 2) revealed that the peak at 1730 cm^−1^ reached its maximum intensity after 24 h of reaction, suggesting a higher modification rate compared to the 6-h condition. Furthermore, the zeta potential was lower in modified-agarose (−10.8 mV for CMA and −7.7 mV for CTA) compared to agarose (1.6 mV) due to the introduction of carboxyl groups (Supplementary Fig. 3). The ^1^H NMR spectra showed a new resonance signal for CMA and CTA at 4.46 ppm and 4.45 ppm, respectively. (Fig. 2b), characteristic of the protons of the methylene group in carboxyl-modified agarose^33^. In the ^13^C NMR spectra, CMA exhibited a peak at 168.8 ppm, corresponding to the carbonyl carbon of the carboxylic acid group^42^, while CTA displayed peaks at 172.3 ppm and 173.4 ppm, attributable to the carboxyl and ester carbon atoms in citric acid (Fig. 2c)^34^. These results collectively confirmed the successful functionalization of agarose with carboxymethyl and citric acid groups.

After confirming the successful introduction of the carboxyl groups onto agarose, we prepared a solution of 0.5% CMA in water, which was subsequently mixed with different divalent metal ions (Ca^2+^, Cu^2+^, and Zn^2+^). These ions are essential for plant development and disease resistance. The results revealed that the gelation time of CMA in the presence of metal ions was significantly reduced to only 3 min (Supplementary Fig. 4a–c), while pure agarose and CMA did not form a gel. As Zn^2+^ is a highly relevant metal ion due to its well-documented role in enhancing plant immunity^41,43,44^, we selected Zn^2+^ for the subsequent studies. As shown in Supplementary Fig. 4c, a similar gelation acceleration was also observed in the CTA-Zn^2+^ system. The enhanced gelation time is likely due to the coordination between carboxyl groups and metal ions, which facilitates the formation of a crosslinked network. These findings also indirectly reaffirmed the successful incorporation of the carboxyl groups onto agarose. We then investigated the incorporation of Phe in addition to Zn^2+^. For this purpose, we employed a simple procedure where the CMA/CTA (0.5% w/v) solution was heated at 90 °C and subsequently cooled to room temperature before the addition of an aqueous solution of ZnCl_2_ (12 mM) and Phe (39 mM). After resting for 3 min, CMA DN gel and CTA DN gel were formed (Fig. 2d). To further elucidate the internal architecture of these hydrogels, cryogenic scanning electron microscopy (cryo-SEM) characterization was conducted. As shown in Fig. 2e, both CMA and CTA hydrogels exhibit a characteristic honeycomb-like porous structure, consistent with previous reports^45^. The CTA hydrogel shows a slightly smaller pore size than the CMA hydrogel, which may lead to differences in their mechanical properties. In contrast, the Phe-Zn^2+^ hydrogel displays a distinct rod-like morphology. Importantly, in both CMA DN and CTA DN hydrogels, rod-like features characteristic of the Phe-Zn^2+^ assemblies are uniformly distributed and embedded within the continuous agarose matrix, indicating the formation of a continuous and interpenetrating network architecture. This observation provides direct morphological evidence supporting the successful construction of a DN system.

To further investigate the gelation behavior, we used a rheometer to monitor the changes in the storage modulus (G’) and loss modulus (G”) over time at room temperature, reflecting the transition from liquid to solid state. We systematically evaluated the gelation behavior of agarose derivatives and Phe-Zn^2+^ complexes by varying the concentrations of agarose and Phe to identify the optimal composition for DN gel formation. Specifically, the mechanical strength of CMA and CTA increased with concentration, while Zn^2+^ shortened their gelation time and improved mechanical strength (Fig. 2f, g, Supplementary Figs. 5, 6a–c). In addition, the CTA-Zn^2+^ gel exhibited markedly higher mechanical strength than the CMA-Zn^2+^ gel, which may be attributed to the higher density of carboxylic groups in CTA, leading to stronger coordination with Zn^2+^, as well as to its smaller pore size (Fig. 2e)^46^.

Similarly, the strength of the hydrogel made of Phe and Zn^2+^ increased as Phe concentration rose (Supplementary Fig. 6d). Balancing mechanical performance and drug release characteristics (detailed in Supplementary Fig. 13), we ultimately selected 0.5% w/v CMA/CTA and 6.5 mg/mL (39 mM) Phe as the optimal formulation for DN gel formation with ZnCl_2_ (12 mM), which yielded CMA and CTA DN gels with excellent mechanical properties (Fig. 2f–h). Notably, the addition of Phe significantly enhanced the mechanical properties of the CMA/CTA-Zn²⁺ hydrogels, and even at a low concentration of 6.5 mg/mL, it can improve their mechanical strength by 5–10 times (Fig. 2f–h).

Overall, these findings demonstrate that Zn^2+^ effectively interacts with carboxyl groups, altering the intrinsic gelation mechanism of agarose gels, which primarily relies on hydrogen bonding^47^. This interaction not only accelerated the hydrogel formation but also significantly enhanced its mechanical properties. Furthermore, Phe allowed to strengthen the rigidity of the CMA/CTA-Zn^2+^gels, resulting in a compact DN structure (Supplementary Fig. 7). These structures offer enhanced compressive resistance and show significant potential for applications in the management of soil-borne plant diseases.

### Acid-responsive and drug release of CMA/CTA DN gels

**Fig. 3:**
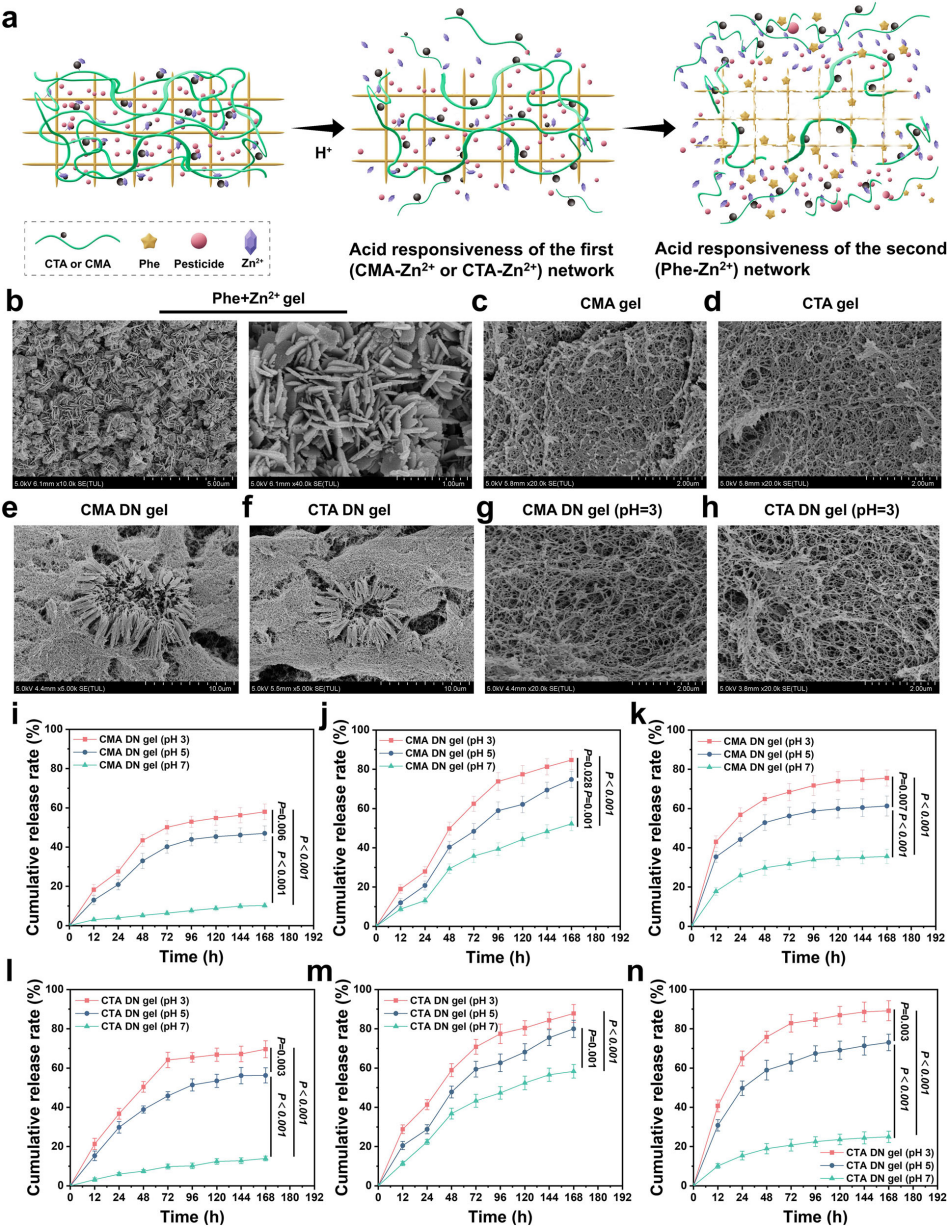
The acidic environment induces the disruption of the crosslinked structure of CMA/CTA DN gels, thereby enhancing the release of active substances, including the pesticide, Zn^2+^, and Phe. **a** Schematic representation of the dual pH-responsive release mechanism of active compounds from the CMA/CTA DN gels. **b**–**h** SEM images of the **b** Phe+Zn^2+^ gel, **c** CMA gel (*i.e*., without Phe), **d** CTA gel (*i.e*., without Phe), **e** CMA DN gel, **f** CTA DN gel, **g** CMA DN gel after exposure to an acid solution (pH = 3), and **h** CTA DN gel after exposure to an acid solution (pH = 3). All observations were independently repeated 3 times to ensure the robustness of the results. Release profiles of **i** Phe, **j** the pesticide, and **k** Zn^2+^ from the CMA DN gels after immersion in solutions at pH 3, 5, and 7 for 7 days. Release profiles of **l** Phe, **m** the pesticide, and **n** Zn^2+^ from the CTA DN gels under the same conditions. In the DN gels, the concentrations of all components were 0.5% w/v CMA/CTA, 6.5 mg/mL (39 mM) Phe, 12 mM ZnCl_2_ and 2 mg/mL pesticide. For panels **i**–**n**, all measurements were performed with 3 technical replicates and the experiments were independently repeated 3 times. Data are presented as mean ± SD (*n* = 3 independent experiments). Statistical differences were determined using one-way ANOVA with a post hoc test. The Phe-Zn^2+^ fibers are visible in the middle of the images (**e**, **f**).

Theoretically, the CMA/CTA DN gels possess sensitive acid-responsiveness, as both the CMA/CTA-Zn^2+^ gel and the Phe-Zn^2+^ gel inherently exhibit acid-responsive properties^37,48,49^. In an acidic environment, the protonation of the carboxyl groups on the CMA/CTA chains weakens the binding affinity between the carboxylic groups and Zn^2+^, leading to the decomplexation and degradation of the crosslinked hydrogel network (Fig. 3a). This favors the interaction between the acidic environment and Phe-Zn^2+^ network, disrupting the self-assembly between Phe and Zn^2+^ (Fig. 3a). The degradation of this secondary network significantly undermines the structural integrity of the DN gel, thereby accelerating drug release (Fig. 3a). To validate the hypothesis of acid-triggered delivery, we conducted acid-responsiveness experiments by exposing the Phe-Zn^2+^ gel to acidic solutions. The results showed that the Phe-Zn^2+^ gel rapidly dissolved in HCl solution (1 M) within 1 min, while it remained stable in a pH 7 solution (Supplementary Fig. 8a, b). However, when exposed to a solution at pH 1, only part of the gel gradually dissolved within 2 h, confirming the acid-responsiveness of the Phe-Zn^2+^ gel (Supplementary Fig. 8c). Next, we constructed DN gels based on Phe-Zn^2+^ and CMA/CTA-Zn^2+^, incorporating rhodamine B to visually investigate their acid-responsiveness. Compared to the pH 7 solution, the DN gel rapidly released rhodamine B in a pH 1 solution (Supplementary Fig. 9a). Surprisingly, the DN gel also maintained high acid-responsiveness at pH 3 and 5 and released more rhodamine B (Supplementary Fig. 9b). Indeed, the fluorescence intensity of the released solution under UV light irradiation was significantly higher than that in the pH 7 solution (Supplementary Fig. 9c).

To further support the above results, we utilized transmission electron microscopy (TEM) and SEM to observe the changes in the crosslinked network structure of the DN gel before and after acid treatment (Fig. 3b–h, Supplementary Fig. 10). The TEM images revealed that, compared to the DN hydrogel at pH 7, the amino acid fibers formed by the Phe-Zn^2+^ self-assembly seem to be no longer visible after treating the gel with a pH 3 solution (Supplementary Fig. 10), confirming that the acidic environment disrupts the DN gel structure. Furthermore, the SEM observations revealed the rod-like self-assembled structure of Phe-Zn^2+^ gel (Fig. 3b) and the dense crosslinked network within the CMA/CTA gel (Fig. 3c, d)^37,50^. Additionally, we clearly observed two distinct crosslinked network structures in the CMA/CTA DN gel, one of which is a stacked rod-like network structure similar to the Phe-Zn^2+^ self-assembled rods, although some local inhomogeneity is present (Fig. 3e, f). After acid treatment, the crosslinked network of the CMA/CTA DN gel exhibited a markedly enlarged pore size (Fig. 3c, d, g, h). More importantly, we observed that the Phe-Zn^2+^ self-assembled structure disappeared from the CMA/CTA DN gel (Fig. 3g, h), resembling the CMA/CTA-Zn^2+^ gel devoid of Phe-Zn^2+^ (Fig. 3c, d), further supporting our hypothesis (Fig. 3a).

To exploit the capacity of the CMA/CTA DN gels for acid-responsive drug release, we incorporated the pesticide into the DN gels. The pesticide-loaded CMA/CTA DN gels were immersed in pH 3, 5, and 7 solutions, and the concentrations of pesticide, Zn^2+^, and Phe in the released solutions were measured over time. We first established the calibration curves for the pesticide, Phe, and Zn^2+^ by HPLC or UV-Vis spectrophotometry (Supplementary Figs. 11, 12), which were used to assess their concentrations in the released solutions under different conditions. As shown in Fig. 3i–n and Supplementary Fig. S13, the concentrations of both CMA/CTA and Phe influenced the drug release rate of the DN gels, with higher concentrations resulting in slower release (Supplementary Fig. 13), likely due to the formation of a denser crosslinked network that impedes molecular diffusion. This further supports our previous conclusion that 0.5% CMA/CTA and 6.5 mg/mL Phe represent the optimal concentration for the DN gels, as they not only balance mechanical performance and material cost, but also result in significantly higher release rates of Phe, pesticide, and Zn^2+^ at pH 3 and 5 compared to pH 7. Specifically, at pH 3, the Phe release rate exceeded 50% for both CMA and CTA DN gels (Fig. 3i, l), while the release rates of pesticide and Zn^2+^ were both over 75% for both CMA and CTA DN gels (Fig. 3j, k, m, n). Moreover, the release rate of Phe and Zn^2+^ from the CTA DN gels was higher than that from the CMA DN gels, likely due to the higher number of carboxyl groups on citric acid, making CTA more sensitive to the acidic environments. At the same time, an acid-triggered drug release trend was also observed in the agarose DN gels, likely due to the acid-responsive behavior of the Phe-Zn^2+^ network (Supplementary Fig. 14). However, the cumulative release rates of all components in the agarose DN gels at pH 3 remained below 50%, which was significantly lower than those of the CMA/CTA DN gels under the same conditions. This further confirms the superior dual pH-responsiveness of the CMA/CTA DN gels for controlled drug release. In summary, our results clearly demonstrate that the CMA/CTA DN gels exhibit sensitive acid-responsiveness and can achieve controlled drug release under specific acidic conditions. This property provides great potential for application in tomato wilt control.

### pH-responsive antibacterial activity in vitro

**Fig. 4:**
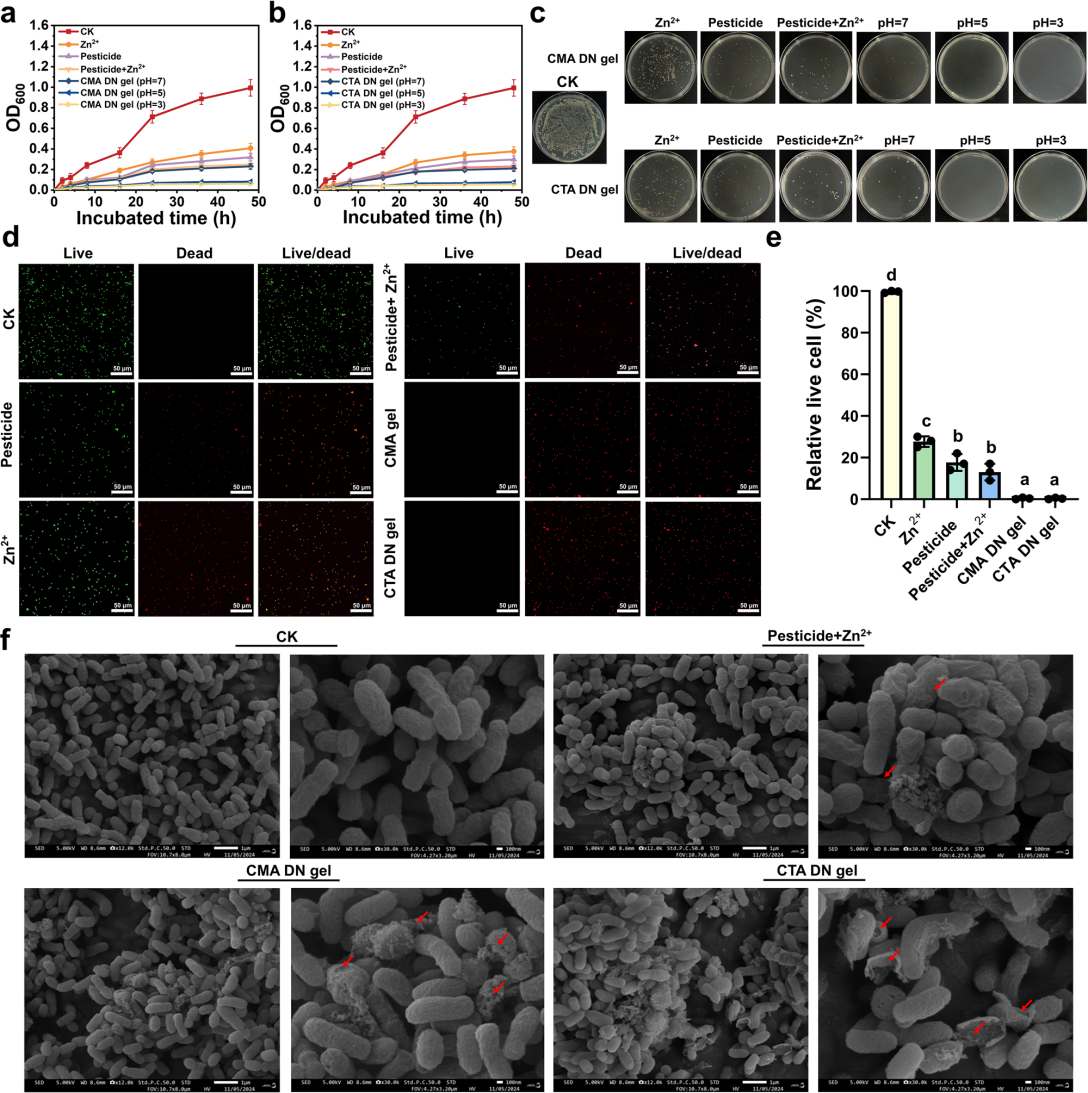
In vitro assessment of the pH-responsive antibacterial efficacy of the CMA/CTA DN gels against *R. solanacearum* under varying pH conditions (pH 3, 5, and 7). **a**, **b** Growth curves of *R. solanacearum* over a 48-h period following exposure to Zn^2+^, the pesticide, pesticide+Zn^2+^, the CMA DN gel, (**a**) and the CTA DN gel (**b**). For panels **a**, **b**, all measurements were performed with 6 technical replicates and 3 biological replicates for each group. Data are presented as mean ± SD (*n* = 3 independent experiments). **c** Inhibition of single-colony growth of *R. solanacearum* by Zn^2+^, the pesticide, pesticide+Zn^2+^, the CMA DN gel, and the CTA DN gel. **d** Live/dead staining analysis to evaluate bacterial viability in the treated and control groups. All observations were independently repeated 3 times to ensure the robustness of the results. **e** Quantitative analysis of the relative live cell percentage determined by the ratio of green fluorescent spots to the total number of green and red fluorescent spots in the live/dead stained cells. All measurements were performed with 3 technical replicates and 3 biological replicates for each group. Data are presented as mean ± SD (*n* = 3 independent experiments). The mean values presented in each bar, accompanied by different letters (**a**–**d**, *P* < 0.05), indicate significant differences based on one-way ANOVA with post hoc test (Relative live cell (%): *F*_*5, 12*_ = 632.304, *p* < 0.001). **f** SEM images illustrating the morphological alterations of *R. solanacearum* cells after treatment with the CMA/CTA DN gels’ released solutions, with red arrows indicating damaged bacterial cells. Prior to testing, all released solutions were adjusted to pH 7 to ensure comparability. All observations were independently repeated 3 times to ensure the robustness of the results.

To assess the antibacterial performance of the CMA/CTA DN gels, the minimum inhibitory concentrations of Zn^2+^ and the pesticide were first determined by evaluating their respective antibacterial activities across a range of concentrations. As shown in Supplementary Fig. 15, Zn^2+^ concentrations exceeding 20 μg/mL and pesticide concentrations above 40 μg/mL effectively inhibited the proliferation of *R. solanacearum* within 48 h (Supplementary Fig. 15a, b). Plate assays further confirmed that, at the same concentrations, Zn^2+^ and the pesticide completely suppressed bacterial colony growth (Supplementary Fig. 15e), with an inhibition rate of 100% (Supplementary Fig. 15c, d). These findings allowed us to establish that Zn^2+^ and the pesticide exhibit significant inhibitory effects on *R. solanacearum* even at low concentrations^51,52^. We then evaluated the pH-responsive antibacterial performance of the CMA/CTA DN gels by testing the antibacterial activity of the released solutions after immersion in solutions at pH 3, 5, and 7 for 7 days. In these experiments, distilled water was used as the negative control (CK), while Zn^2+^, the pesticide, and the mixture of pesticide+Zn^2+^ served as positive controls, with concentrations similar to those released by the CMA/CTA DN gels at pH 7. As shown in Fig. 4a–c, under acidic conditions (pH 3 and pH 5), the CMA/CTA DN gel-treated groups completely inhibited the proliferation of *R. solanacearum* (Fig. 4a, b) and significantly suppressed single-colony growth (Fig. 4c, Supplementary Fig. 16). Statistical analysis of the inhibition rates revealed that the CMA/CTA DN gels achieved 100% inhibition at pH 3 and pH 5 (Supplementary Fig. 16), substantially outperforming all control groups. This effect can be attributed to the higher concentrations of Zn^2+^ and pesticide in the released solutions under acidic conditions compared to a neutral environment and the control. However, in the control agarose-Phe-Zn^2+^ DN gels, there was no significant difference in the inhibitory effect on the growth of *R. solanacearum* across the different pH-treated groups (Supplementary Fig. 17).

To visualize the inhibitory effect of the CMA/CTA DN gels on *R. solanacearum*, we performed fluorescence staining to assess the bacterial cell viability. Confocal microscopy images showed that the bacteria treated with distilled water exhibited green fluorescence (live cells stained with SYTO9 dye) and red fluorescence (dead cells stained with propidium iodide) (Fig. 4d). In contrast, the treatments with Zn^2+^, the pesticide, or pesticide+Zn^2+^ resulted in substantial red fluorescence signals, with the pesticide+Zn^2+^ group displaying more pronounced red fluorescence than the single-component groups, further confirming the combined antibacterial effect of Zn^2+^ and the pesticide. Notably, in the CMA/CTA DN gel-treated group, no green fluorescence was observed, and all bacteria exhibited red fluorescence, indicating complete bacterial eradication. Quantitative analysis of the fluorescence images corroborated these findings, showing that the proportion of live cells in the CMA/CTA DN gel-treated group was significantly lower than in any control group (Fig. 4e). Additionally, SEM revealed that treatment with pesticide+Zn^2+^ caused structural damage to *R. solanacearum* cells, including some surface rupture and collapse (Fig. 4f). The CMA/CTA DN gel-treated group exhibited even more severe cellular damage, with numerous abnormal, aggregated cell structures observed. In summary, these results demonstrate that the CMA/CTA DN gels possess excellent pH-responsive antibacterial activity, effectively inhibiting the growth and proliferation of *R. solanacearum* through the enhanced release of Zn^2+^ and the pesticide under acidic conditions. These findings highlight the potential of the CMA/CTA DN gels for controlling *R. solanacearum* in acidic environments.

### Pot experiments and root bacterial infection visualization

**Fig. 5:**
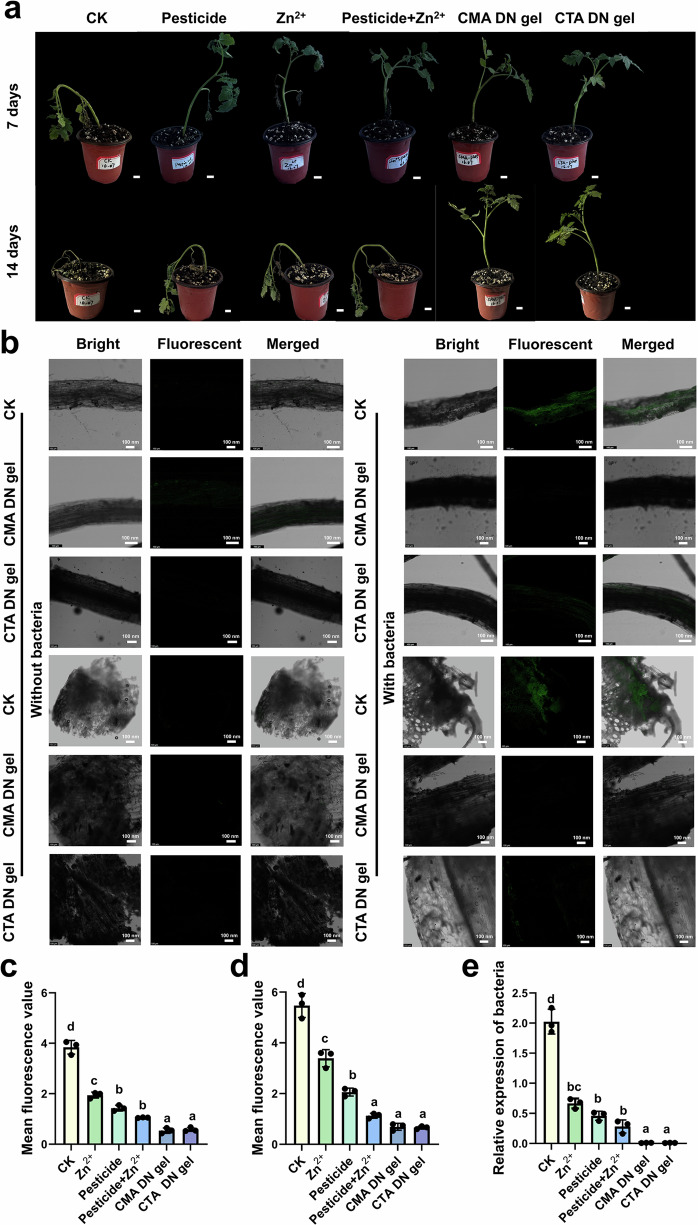
In vivo assessment of the protective efficacy of the CMA/CTA DN gels against *R. solanacearum* infection in tomato plants. **a** Representative photographs of tomato plants exhibiting symptoms of bacterial wilt after treatment with the CMA/CTA DN gels. **b** Cross-sectional and longitudinal confocal laser scanning microscopy images of tomato roots infected with *R. solanacearum*-GFP, showing bacterial distribution following the treatment with the DN gels (the white scale bars indicate 1 cm). All observations were independently repeated 3 times to ensure the robustness of the results. **c**, **d** Quantitative analysis of fluorescence intensity in **c** cross-sectional and **d** longitudinal root images to assess *R. solanacearum*-GFP load after hydrogel treatment. For panels (**c**, **d**), all measurements were performed with 3 technical replicates and 3 biological replicates for each group. Data are presented as mean ± SD (*n* = 3 independent experiments). **e** qPCR analysis of *R. solanacearum*-GFP abundance in tomato roots post-treatment with the CMA and CTA DN gels. CK denotes control plants treated with deionized water. All measurements were performed with 3 technical replicates and 3 biological replicates for each group. Data are presented as mean ± SD (*n* = 3 independent experiments). The mean values presented in each bar, accompanied by different letters in panels (**c**–**e**) (*P* < 0.05), indicate significant differences based on one-way ANOVA with post hoc test (Mean fluorescence value (panel **c**): *F*_*5, 12*_ = 230.146, *p* < 0.001, Mean fluorescence value (panel d): *F*_*5, 12*_ = 164.365, *p* < 0.001, Relative ex*p*ression of bacterial: *F*_*5, 12*_ = 154.061, *p* < 0.001).

Bacterial wilt is a devastating disease characterized by the rapid death of infected plants within just 3 to 7 days^53^. As previously noted, acidic soil conditions (pH < 5) are a critical factor in the rapid onset and severe field outbreaks of this disease^11^. Therefore, an optimal strategy for effective disease control lies in exploiting the relationship between *R. solanacearum* and soil pH. By targeting the optimal pH range for bacterial growth, therapeutic agents can be applied to inhibit bacterial proliferation, thereby preventing plant infection^20^. In this context, the most efficient approach involves the use of smart drug carriers that can sense soil pH changes and release antibacterial agents precisely under optimal conditions. Our aforementioned data demonstrate that CMA/CTA DN gels exhibit excellent acid-responsive antibacterial activity in vitro. These gels effectively release the pesticide and Zn^2+^ in solutions at pH 3 and 5, completely inhibiting bacterial growth. To evaluate the practical efficacy of the CMA/CTA DN gels in controlling bacterial wilt, pot experiments were conducted. The first step involved investigating the absorption of hydrogel-released agents by the plant roots and their translocation within the plant. For this purpose, 5(6)-carboxyfluorescein was used as a substitute for the pesticide and loaded into the DN gels, which were subsequently applied to tomato plants grown in mildly acidic soil (pH 5). After seven days of treatment, the root and the stem sections near the roots were analyzed using confocal microscopy. As shown in Supplementary Figs. 18, 19, no fluorescence signals were observed in the root and stem segments of the water-treated control group. In contrast, a strong green fluorescence was detected in both roots and stems treated with the dye solution (positive control group), indicating that the dye permeated the soil and was absorbed by the plants. Similarly, fluorescence signals were observed in the root and stem tissues of the CMA/CTA DN gel-treated groups. This confirms that the DN gels released the dye in acidic soil, which was subsequently absorbed by the plant roots and transported to the stem. These findings provide direct evidence supporting the potential role of the CMA/CTA DN gels in controlling bacterial wilt. This is particularly relevant given the disease’s pathogenic mechanism, in which the bacteria invade through the roots, colonize the vascular tissues of the root and lower stem, and ultimately cause plant death^54^. Hence, the ability of the hydrogel-released agents to be absorbed and translocated to the lower stem is critical for the effective prevention of bacterial wilt, in addition to their antibacterial action in soil^20^. Tomato plants were then treated with the pesticide-loaded CMA/CTA DN gels and inoculated with *R. solanacearum* in the soil. The disease incidence was assessed at 7- and 14-day post-inoculation (dpi). As shown in Fig. 5a, the typical symptoms of bacterial wilt, such as leaf and stem wilting, were evident in the water-treated control group at 7 dpi. The Zn^2+^-treated and pesticide-treated alone groups also displayed wilting of the basal leaves. However, no symptoms of bacterial wilt were observed in the pesticide+Zn^2+^ group and the CMA/CTA DN gel-treated groups at this stage. At 14 dpi, the plants in the water-treated group were completely wilted, and those in the Zn^2+^ and pesticide-treated groups showed similar symptoms. The pesticide+Zn^2+^ group also exhibited severe bacterial wilt symptoms. In contrast, the plants in the CMA/CTA DN gel-treated group showed no observable symptoms, with only a few displaying slight wilting of basal leaves. The disease severity index further validated these findings, with the CMA/CTA DN gel-treated-groups showing significantly lower disease index (CMA DN gel: 12.5 and CTA DN gel: 8.3, Supplementary Table 1) compared to the water-treated control (100) and other positive-control groups (66.7–87.5). These findings confirm that, although Zn^2+^ and the pesticide can each moderately delay disease onset, and their combination shows an enhanced effect, neither treatment is able to prevent disease occurrence, as evident from the clear wilting symptoms observed at 14 dpi. In contrast, the CMA/CTA DN gels demonstrate greater potential as a disease management strategy by effectively suppressing bacterial infection in tomatoes and preventing disease development over an extended period. This superior efficacy is likely attributed to the unique drug release mechanism of the gels. Under acidic conditions, the gels rapidly release a high amount of active agents to target *R. solanacearum* in the soil, significantly reducing bacterial populations. Their sustained release mechanism ensures prolonged delivery of the agents, preventing reinfection by residual bacteria and acting on plants during early infection stages to inhibit the disease development. In contrast, direct application of Zn^2+^ or pesticides provides only a transient contact with the bacteria, with most of the agents lost due to irrigation and rainwater infiltration, thereby reducing their efficacy. This assumption was subsequently confirmed by the field experiments (*vide infra*). Therefore, the CMA/CTA DN gels not only enable precise, pH-responsive drug release for efficient bacterial control but also minimize pesticide loss, thus enhancing drug efficiency while reducing environmental pollution. These properties make the CMA/CTA DN gels a highly effective and environmentally friendly solution for bacterial wilt management.

In addition, we employed a genetically modified *R. solanacearum* labeled with GFP (*R. solanacearum*-GFP) to infect the plants, providing a direct visualization of bacterial invasion into the plant roots and assessing the ability of the CMA/CTA DN gels to prevent infections. Initially, we performed in vitro experiments by exposing *R. solanacearum*-GFP to the drug-release solutions derived from the CMA/CTA DN gels at pH 5 to investigate their inhibitory effects on the bacteria. As shown in Supplementary Fig. 20, the control group exhibited a large number of uniformly distributed *R. solanacearum* cells, accompanied by strong green fluorescence signals generated by GFP. However, no bacterial cells or GFP fluorescence signals were observed in the CMA/CTA DN gel-treated groups, indicating that the gels completely inhibited the growth and proliferation of *R. solanacearum*-GFP. Subsequently, *R. solanacearum*-GFP was used to inoculate tomato plants pre-treated with the CMA/CTA DN gels. The control groups included the plants treated with water, Zn^2+^, the pesticide, and pesticide+Zn^2+^. After 14 dpi, the root samples were collected to evaluate the extent of bacterial infection through GFP fluorescence signals. As expected, no signals were detected in the roots of the healthy plants treated with water (Fig. 5b). However, strong GFP fluorescence signals were observed in both cross-sections and longitudinal sections of roots inoculated with *R. solanacearum*-GFP. While the plants treated with Zn^2+^, the pesticide, or pesticide+Zn^2+^ exhibited reduced GFP signals compared to the untreated control (Supplementary Fig. 21), fluorescence was still apparent, suggesting that Zn^2+^ and the pesticide provided only limited protection against the bacterial infection. Notably, in the plants treated with the CMA/CTA DN gels, GFP fluorescence signals were remarkably diminished and observed only in a few isolated cases (Fig. 5b). This strongly suggests that the CMA/CTA DN gels effectively blocked *R. solanacearum* infection in plant roots. To further confirm these results, the fluorescence intensity (Fig. 5c, d) was quantified from the images, and the *R. solanacearum*-GFP load in the root tissues was assessed by qPCR analysis across the different treatment groups (Fig. 5e). The results demonstrated that, in comparison to the control groups, the CMA/CTA DN gel-treated groups exhibited a distinctly reduced GFP fluorescence intensity (Fig. 5c, d) and an almost undetectable level of *R. solanacearum* RNA expression in the roots (Fig. 5e). These findings demonstrate that the CMA/CTA DN gels effectively inhibit *R. solanacearum* infection in plant roots, significantly reducing the incidence of bacterial wilt disease.

### Biological safety and growth-promoting effects of the CMA/CTA DN gels

**Fig. 6:**
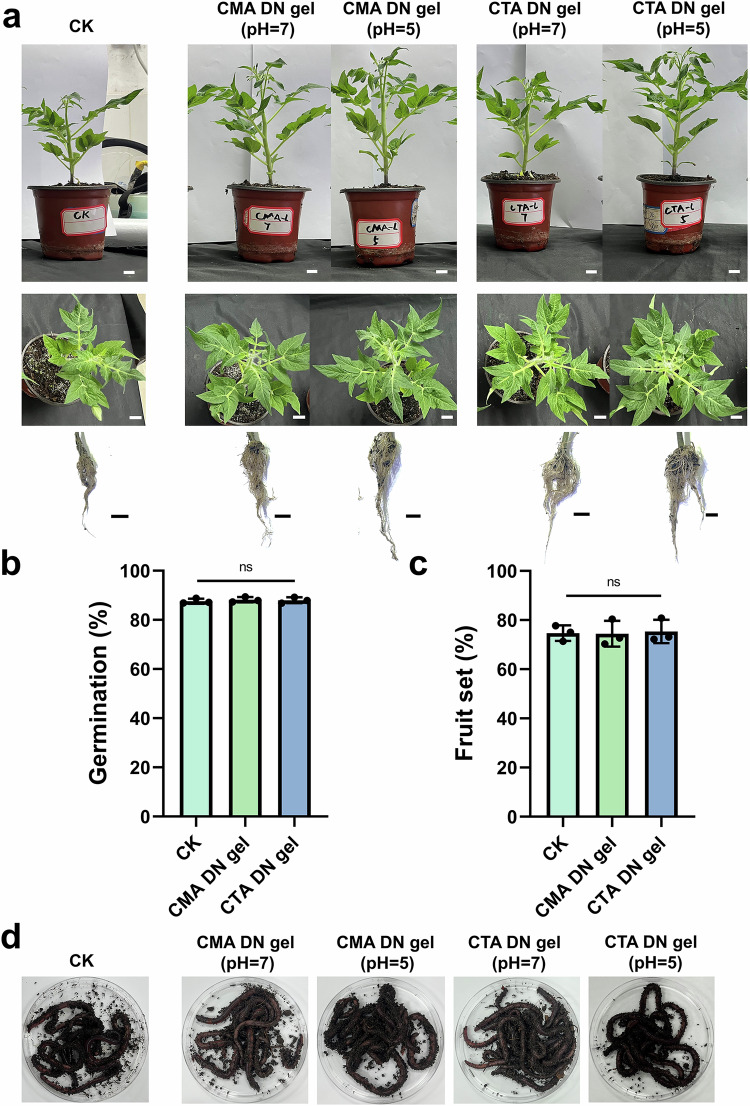
Biosafety evaluation of the CMA and CTA DN gels. **a** Morphological assessment of tomato plants, including plant height, width, and root length, after a 3-week exposure to the solutions released from the CMA and CTA DN gels under acidic (pH 5) and neutral (pH 7) conditions. **b** Statistical analysis of pollen germination rates in each treatment group following treatment of tomato plants with the CMA and CTA DN gels under acidic conditions (pH 5). All measurements were performed with 4 technical replicates and 3 biological replicates for each group. Data are presented as mean ± SD (*n* = 3 independent experiments). **c** Statistical analysis of the fruit set rate of the first inflorescence in each treated group following the treatment of tomato plants with the CMA and CTA DN gels under acidic conditions (pH 5). All measurements were performed with 3 technical replicates and 3 biological replicates for each group. Data are presented as mean ± SD (*n* = 3 independent experiments). **d** Representative images of earthworms after 7 days of exposure to soils adjusted to different pH levels (pH 5 and 7) and supplemented with the CMA or CTA DN gels. Prior to testing, all released solutions were adjusted to pH 7 to ensure comparability. The white and black scale bars in panel a indicate 1 cm.

Zn^2+^ and Phe are important nutrients for plant growth and development, and fertilizers based on Zn^2+^ and Phe have been widely used in agricultural cultivation^55,56^. In the drug release experiments (Fig. 3i–n), we demonstrated that the CMA/CTA DN gels can release a large amount of Zn^2+^ and Phe in acidic environments. Thus, the CMA/CTA DN gels could function as fertilizer delivery carriers by releasing Zn^2+^ and Phe, thereby enhancing plant growth. To validate our hypothesis, we investigated the impact of the CMA/CTA DN gels on tomato growth. In order to avoid the influence of varying soil pH on plant development and ensure consistency in our results, we opted to irrigate the plants with solutions released from the CMA/CTA DN gels at different pH levels (pH 5 and 7), with all solutions adjusted to pH 7 before use. The plant height, plant width, fresh weight, dry weight, and root length were measured as key growth indicators. As shown in Supplementary Fig. 22, the Zn^2+^ content in the roots, stems, and leaves of the tomatoes treated with the CMA/CTA DN gels was significantly higher than that of the water control group, and the content in acidic environments (pH 5) was significantly higher than in neutral environments (pH 7). After three weeks of treatment, it was clear that the plants treated with the CMA/CTA DN gels grew significantly better than the control groups, with increase in plant height, plant width, root length, and better growth in the acidic release solution (Fig. 6a). The growth metrics indicated that the fresh weight, dry weight, plant height, plant width, and root length of the plants treated with the CMA/CTA DN gels were significantly higher than the control group (Supplementary Fig. 23), with higher values under acidic conditions compared to neutral conditions. This indicates that the CMA/CTA DN gels can promote plant growth, providing an advantageous solution for integrated plant disease control and offering strategies for sustainable agricultural development. Specifically, we evaluated the pollen germination rates and the fruit set to determine whether the CMA/CTA DN gels affect the reproductive process of tomato plants. The results showed that all treated groups exhibited high pollen viability, with germination rates exceeding 80% (Fig. 6b), while the fruit set rate of the first truss in all groups was above 70% (Fig. 6c), with no significant difference compared to the control. These findings indicate that the CMA/CTA DN gels do not adversely affect the reproductive performance of tomato plants.

Our CMA/CTA DN gels are mainly applied through the roots. Although the components of the hydrogels are biodegradable, they may remain in the soil for an extended period, and their biological safety has yet to be fully determined. Therefore, verifying the biological impact of the CMA/CTA DN gels is crucial in view of their future applications. Seed germination assays are widely recognized as a standard and reliable method for assessing the biological safety of pesticides^57^. Therefore, in addition to testing tomato seeds, we also selected maize and rice, two of the world’s most widely cultivated cereal crops, as experimental subjects, to highlight the broad applicability of the CMA/CTA DN gels. The solutions released from the CMA/CTA DN gels under various pH conditions exhibited no adverse effects on seed germination of the three species, with germination rates approaching 100% across all treatments (Supplementary Figs. 24a, c, d, 25a–c and 26a–c). Notably, under acidic conditions, the CMA/CTA DN gels significantly enhanced seedling growth, with the seedling lengths markedly exceeding those of both control (water) and neutral pH treatments (Supplementary Figs. 24b, e, f, 25a, d, e and 26a, d, e). This growth-promoting effect may be attributed to the synergistic role of Zn^2+^ and Phe in stimulating seed germination and early development. In summary, the CMA/CTA DN gels demonstrate a high biological safety and do not pose a threat to crops when applied in soils.

On the other hand, earthworms are commonly regarded as representative organisms in soil ecotoxicology due to their sensitivity to environmental changes. Thus, assessing the survival of earthworms following exposure to the CMA/CTA DN gels provides additional valuable insights into the ecological safety of the treatments within soil environments^58^. Therefore, we mixed the CMA/CTA DN gels with soils adjusted to different pH levels (pH 5 and 7), and co-cultured earthworms in these treated soils. After 7 days of incubation, the earthworms in both CMA/CTA DN gel-treated and control groups maintained high vitality (Fig. 6d) with survival rates exceeding 85% (Supplementary Fig. 27). These findings indicate that the CMA/CTA DN gels possess high biosafety to beneficial soil organisms.

### Field experiment

Assessing the efficacy in the field is a crucial step in evaluating the practical effectiveness of new materials or pesticides^59^. Indeed, by conducting experiments in real agricultural environments, a comprehensive assessment of the efficacy of new antibacterial agents against target pests, diseases, and weeds can be obtained, as well as their impact on crop yield and quality. Compared to laboratory experiments, field experiments offer a more accurate representation of pesticide performance under complex environmental conditions and allow for the evaluation of their practical applicability, thus providing reliable evidence for large-scale implementation.

**Fig. 7:**
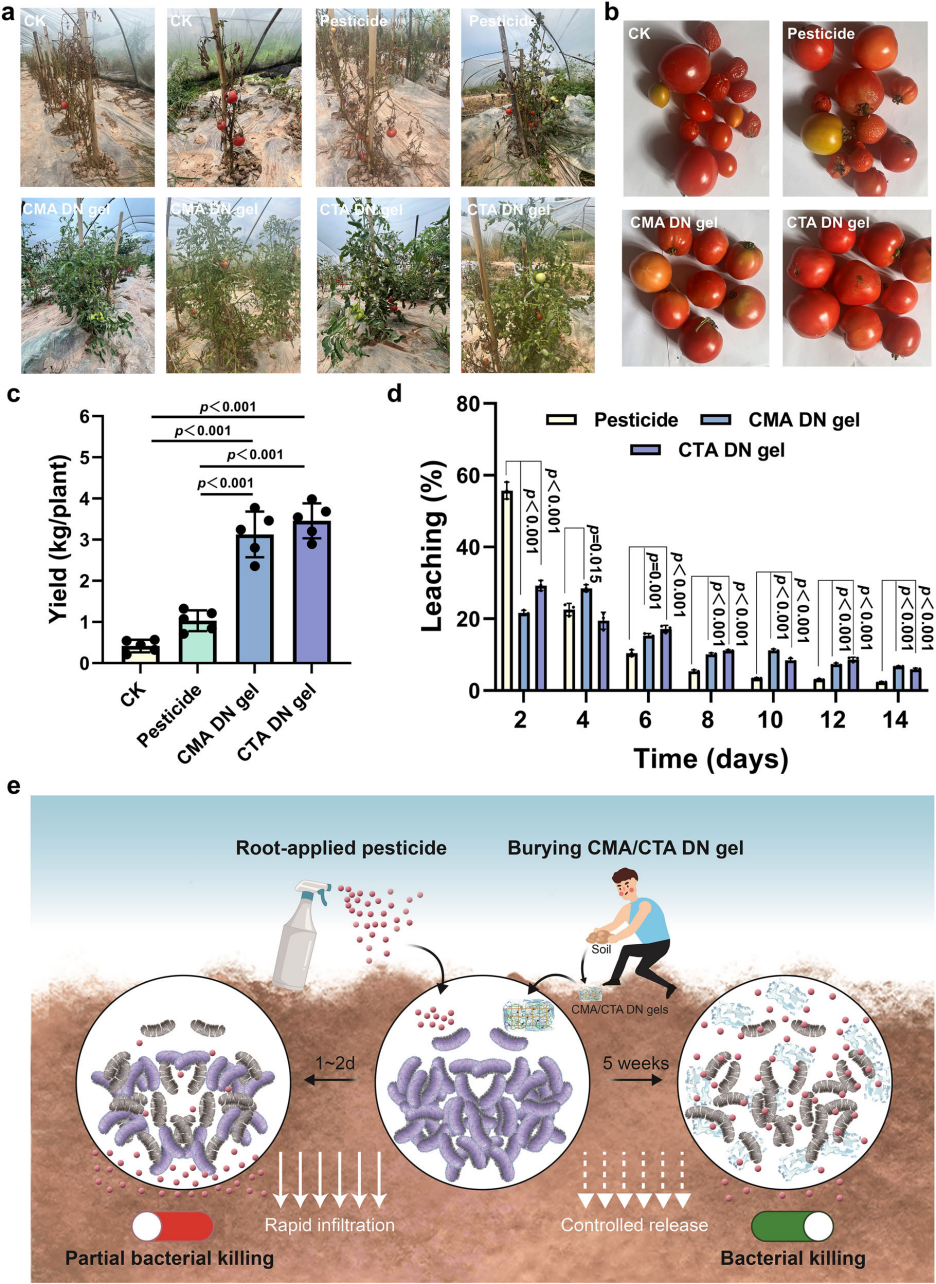
Field evaluation of the integrated efficacy of the CMA/CTA DN gels in managing bacterial wilt in tomato plants. **a** Representative images showing the disease progression in tomato plants from different treated groups, five weeks after field application of the CMA and CTA DN gels. **b** Representative photographs of harvested tomato fruits from each treated group. **c** Statistical analysis of tomato yield across treated groups to assess agronomic performance. All measurements were performed with 30 technical replicates and 3 biological replicates for each group. Data are presented as mean ± SD (*n* = 3 independent experiments). **d** Soil leaching assay comparing the mobility and retention of the pesticide in acidic soil following the treatment with a free pesticide solution *vs* the CMA/CTA DN gels. All measurements were performed with 3 technical replicates and 3 biological replicates for each group. Data are presented as mean ± SD (*n* = 3 independent experiments). **e** A schematic illustration of pesticide leaching behavior in soil after direct root irrigation with a pesticide solution and after localized, controlled, and sustained delivery from the DN gels. Statistical differences were determined using one-way ANOVA with a post hoc test.

Bacterial wilt is a soil-borne disease capable of surviving in the soil for 3 to 10 years or even longer, and it can infect host plants through multiple transmission routes^3^. Its incidence increases significantly under conditions of continuous cropping over several years^3^. Therefore, we conducted field experiments in 2024 and 2025 in tomato fields that had been continuously cultivated and were known for frequent disease outbreaks, allowing infection to occur naturally^60^. This approach was chosen to avoid the potential interference associated with artificial inoculation and to ensure the authenticity and reliability of the experimental data. For this purpose, we selected a tomato field that had been continuously cultivated with tomatoes for five years and was severely affected by bacterial wilt. Using a randomized sampling method, three different planting areas within the field were chosen for the experiment. Based on local climate data and field surveys, July was identified as the peak period for bacterial wilt^61^. Hence, the DN gels were applied at the end of June, prior to the onset of the disease, to maximize preventive efficacy. Water and the pesticide were used as control groups. The CMA/CTA DN gels or the pesticide were applied only once during the experiment, with no additional pesticides or fertilizers applied, except for routine irrigation. Throughout the experiment, the climatic temperature was recorded, with an average of 33.6 ± 3.8 °C (Supplementary Tables 2, 3), which falls within the optimal range for the development of bacterial wilt^62^. As shown in Supplementary Figs. 28–36, two weeks after the treatment, the symptoms of bacterial wilt were observed in some of the plants in the CK and pesticide groups (Supplementary Figs. 28, 33)^63^, while the majority of the plants in the CMA/CTA DN gel-treated groups remained healthy, with only a few showing a slight wilting process. The data revealed that the disease incidence after two weeks was 29.3% and 19.0% (Supplementary Table 4) in the CK and pesticide groups, respectively, which was significantly higher than that of the CMA DN gels (10.1%) and CTA DN gels (8.2%). These findings indicate that, compared to the pesticide alone, the CMA/CTA DN gels exhibited superior inhibitory effects during the early stages of bacterial wilt. Assessing the disease severity index (Supplementary Table 5) confirmed this conclusion, as the CK and pesticide groups showed significantly higher disease severity index than the CMA/CTA DN gel-treated groups. Photographic analyses of the plant symptoms across the different treated groups further substantiate that the CMA/CTA DN gels are capable of significantly mitigating the severity of bacterial wilt and decelerating the disease progression (Supplementary Figs. 28, 33). During the third and fourth weeks of the experiment, disease progression was evident (Supplementary Figs. 29, 30, and 34–36), particularly in the CK and pesticide-treated groups, which showed a clear increase in incidence (Supplementary Table 4) and severity (Supplementary Table 5). In contrast, the CMA DN gel and CTA DN gel groups exhibited much slower disease development, with substantially lower incidence and severity. In addition, after five weeks (Fig. 7a, Supplementary Figs. 31, 32), the disease incidence in the CK and pesticide groups further increased to 81.2% and 63.2% (Supplementary Table 4), respectively, with a severity index of 75.0% and 54.1% (Supplementary Table 5). However, the CMA DN gel and CTA DN gel-treated groups maintained significantly lower disease incidences of 35.9% and 28.2%, with severity index of 25.2% and 19.9%, respectively. These findings demonstrate that the CMA/CTA DN gels possess strong potential to suppress the disease development and effectively reduce plant mortality during both the onset and progression stages of bacterial wilt. Moreover, the plant growth in the CMA/CTA DN gel-treated groups was notably better than that in the CK and pesticide groups (Supplementary Table 6), producing fruits with improved quality (Fig. 7b) and significantly higher yields (Fig. 7c). Although the yields were lower than typical production levels ( ≈ 4.5 kg/plant)^64^, this was primarily due to the single gel application and the absence of additional pesticides or fertilizers during the experiment.

During the experiment, the soil pH was continuously monitored. As shown in Supplementary Fig. 37, the soil maintained an acidic profile throughout the disease progression period, likely resulting from prolonged agricultural activities^65^. This acidic environment favored the pH-responsive release behavior of the CMA/CTA DN gels, thereby enhancing their effectiveness in the suppression of the disease. Notably, the soil pH in the CMA/CTA DN gel-treated plots was slightly higher than that observed in the CK and pesticide-treated groups. This difference may be related to the disease incidence, as elevated bacterial activity associated with higher disease severity in the CK and pesticide groups likely led to the production of acidic metabolites, which could interact with tomato root exudates and further lowering soil pH^11,13^. Furthermore, the CMA/CTA DN gels themselves exhibit mild alkalinity, similar to soil conditioners, which can partially neutralize soil acidity, thereby helping to suppress the disease development^66^. This weak alkaline nature (pH around 8.0–8.5) arises from the Phe-Zn^2+^ network within the hydrogel that is formed at pH 8.5. Nonetheless, throughout this experiment, we observed significant differences in disease control efficiency between the pesticide and the CMA/CTA DN gel-treated groups. We hypothesize that these differences are due to the pH-responsiveness of the gels and their sustained-release properties. To verify this hypothesis, acidic soil samples from the field experiment were collected and subjected to leaching experiments under laboratory conditions to simulate the infiltration and release dynamics of conventional pesticides and CMA/CTA DN gels in soil environments (Supplementary Fig. 38). The pesticide-treated soil exhibited high concentrations of pesticide within the first three days, followed by a marked decline, suggesting rapid leaching and significant loss of efficacy (Fig. 7d, Supplementary Fig. 38). In contrast, the CMA/CTA DN gel-treated soil not only demonstrated similarly high initial concentrations, but also maintained substantial pesticide levels over an extended period (Fig. 7d). When combined with the drug release profiles (Fig. 3i–n), these data clearly support the hypothesis that the CMA/CTA DN gels enable pH-triggered, sustained pesticide release in acidic soil conditions. The persistent acidic soil environment and the inherent sustained-release properties of the gels allow a prolonged pesticide release, resulting in efficient and continuous control of *R. solanacearum* in the soil, ultimately suppressing bacterial wilt effectively, as illustrated in Fig. 7e. Simultaneously, soil-based degradation experiments further confirmed these observations (Supplementary Fig. 39), demonstrating that the CMA DN gels and CTA DN gels remain observable by the naked eye in soil for approximately one month. A clear, gradual degradation of the hydrogels was monitored, and this process facilitates the sustained release of the encapsulated pesticide. Moreover, the slow degradation also guarantees prolonged retention of the active agent at the site of disease occurrence, supporting extended bioactivity. Overall, these findings highlight the potential of the CMA/CTA DN gels as a multifunctional platform for disease management, offering both effective control of bacterial wilt and improved crop performance. This strategy represents a promising advancement for practical implementation and large-scale adoption in sustainable agricultural practices.

### Metagenomic assessment of the CMA/CTA DN gels on soil microbiomes

**Fig. 8:**
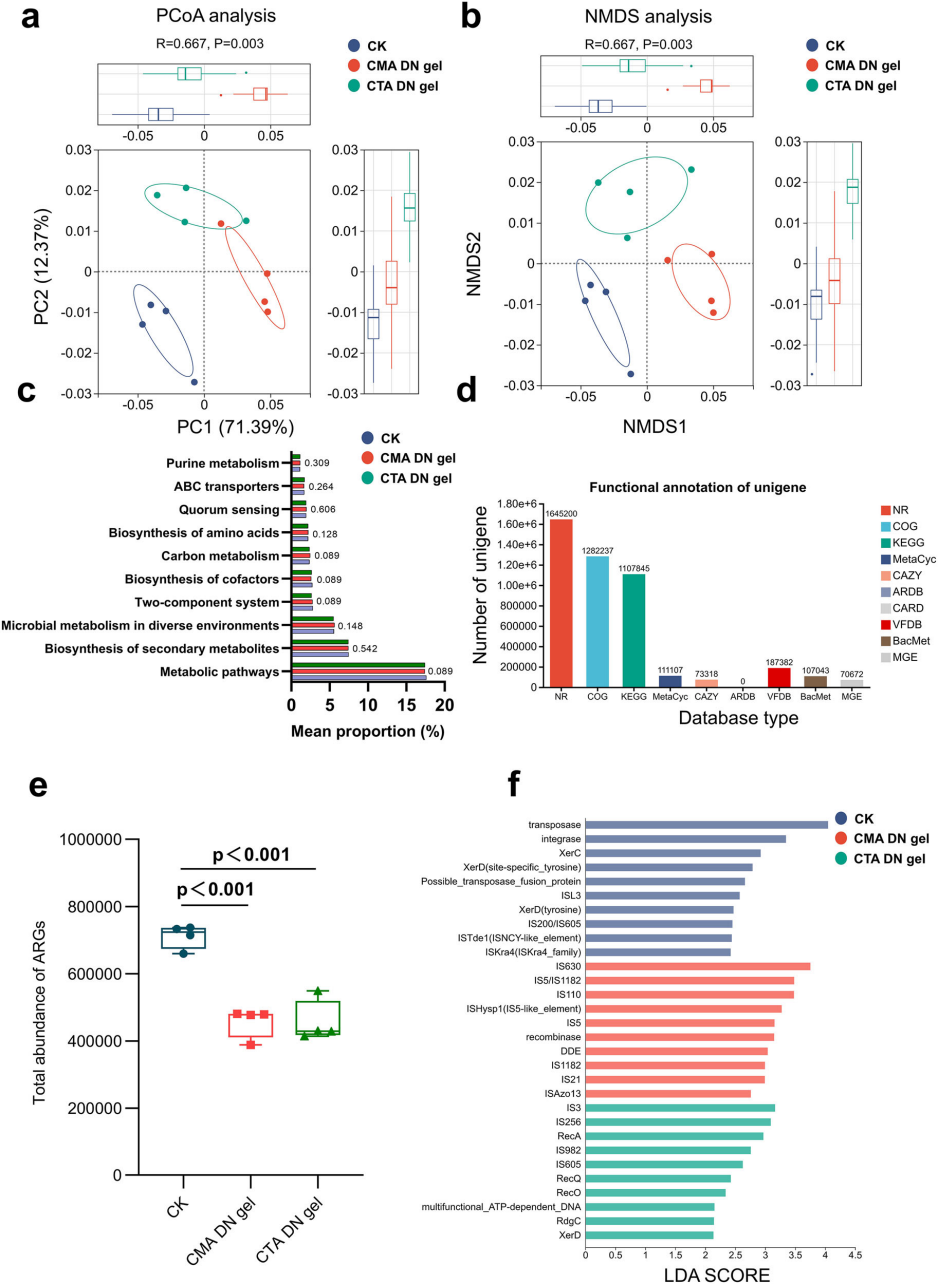
Metagenomic analysis of soil samples collected 7 months after the treatment with the CMA and CTA DN gels. **a** Principal coordinates analysis (PCoA). Box plots: whiskers represent minima (0th percentile) and maxima (100th percentile); boxes span lower (25th) and upper (75th) quartiles; center lines show medians (50th percentile). CK: min=0.0042, max = −0.0693, median = −0.0344, 25th = −0.0233, 75th = −0.0417; CMA: min=0.0631, max=0.0221, median=0.0466, 25th = 0.0477, 75th = 0.0375; CTA: min=0.0241, max = −0.0460, median = −0.0140, 25th = −0.0022, 75th = −0.0197. All measurements were performed with 4 technical replicates. Data are presented as the mean of independent samples. **b** Non-metric multidimensional scaling (NMDS). Box plots: whiskers represent minima (0th percentile) and maxima (100th percentile); boxes span lower (25th) and upper (75th) quartiles; center lines show medians (50th percentile). CK: min = −0.0694, max = −0.0004, median = −0.0367, 25th = −0.0435, 75th = −0.0263; CMA: min = 0.0272, max = 0.0624, median = 0.0488, 25th = 0.0404, 75th = 0.0492; CTA: min = −0.0489, max = 0.0273, median = −0.0138, 25th = −0.0203, 75th = −0.0013. All measurements were performed with 4 technical replicates. Data are presented as the mean of independent samples. **c** Statistical significance testing based on KEGG profiling. **d** Summary of CARD annotation. **e** Comparison of total ARG abundance. Box plots: whiskers represent minima (0th percentile) and maxima (100th percentile); boxes span lower (25th) and upper (75th) quartiles; center lines show medians (50th percentile). CK: min = 659700, max = 736934, median = 723931, 25th = 687207, 75th = 734845; CMA: min = 387882, max = 480762, median = 477962, 25th = 432567, 75th = 479439; CTA: min = 413418, max = 548742, median = 428597, 25th = 420557, 75th = 461608. All measurements were performed with 4 technical replicates. Data are presented as the mean of independent samples. **f** LEfSe analysis (LDA stands for Linear Discriminant Analysis). For soil metagenomic sequencing, four independent samples were analyzed for each group to ensure the reliability of the results. Statistical differences were determined using one-way ANOVA with a post hoc test.

To systematically evaluate the long-term ecological impact of the CMA/CTA DN gels under field conditions, we performed a metagenomic sequencing on soil samples collected approximately seven months after hydrogel applications. During this period, no additional crops were planted, and no agricultural practices were conducted, minimizing external disturbances and allowing a more direct assessment of microbial responses. Beta-diversity analysis based on Bray-Curtis distance revealed a clear and statistically significant separation between CK and CMA/CTA-treated soils (Analysis of Similarities (ANOSIM) *R* = 0.667, *P* = 0.003; Non-metric Multidimensional Scaling (NMDS) stress = 0.038; Figs. 8a, b), suggesting a treatment-driven shift in the community composition. Despite this separation, sequencing depth, gene richness, and assembly quality remained comparable across all groups (Supplementary Tables 7, 8). The dominant phyla were consistently preserved, and the overall taxonomic profile showed no signs of imbalance. Together, these patterns point to community restructuring rather than ecological disruption. A similar trend was observed at the functional level. Kyoto Encyclopedia of Genes and Genomes (KEGG) profiling showed that core metabolic pathways remained highly stable among the treatments, with metabolic pathways ( ~ 17.4–17.6%) and biosynthesis of secondary metabolites ( ~ 7.4%) exhibiting minimal variation (Fig. 8c, Supplementary Data 1). Although several pathways reached nominal significance (*P* < 0.05), these differences were not retained after False Discovery Rate (FDR) correction (*q* ≈ 0.089) (Fig. 8c, Supplementary Data 1), suggesting that the observed variations were not deleterious/dramatic/influent. Thus, this composition shift did not translate into functional impairment of key metabolic processes. Comprehensive antibiotic resistance database (CARD) annotation identified antibiotic resistance genes (ARG)-related genes across all samples (84250 annotated unigenes), predominantly belonging to commonly occurring efflux pump families (e.g., patA and emrB; Fig. 8d, Supplementary Data 2 (ARO abundance)). Linear discriminant analysis Effect Size (LEfSe) analysis further detected limited enrichment of two markers (Erm(31) and AxyY, *P* = 0.023) in the CMA group (Supplementary Table 9). At the community level, however, total ARG abundance was lower in CMA- and CTA-treated soils than in the control (Fig. 8e), and major resistance categories, including β-lactams, glycopeptides, and multidrug resistance, did not show coordinated enrichment. This pattern was further supported by the analysis of mobile genetic elements (MGEs) (Fig. 8f). Transposase, integrase, and recombinase genes were detected across all groups; their abundance remained statistically comparable. In addition, no coupled increase between ARGs and MGEs was observed, suggesting limited potential for enhanced horizontal gene transfer under these conditions. Taken together, the CMA and CTA DN gels induced measurable shifts in microbial community composition without disrupting core ecosystem functions. The absence of coordinated ARG expansion and MGE enrichment further suggests that the system does not promote resistance proliferation under the tested conditions. This favorable profile is likely related to the intrinsic properties of the materials. Agarose and Phe are natural polymers and amino acids, respectively, and are generally considered biocompatible and environmentally benign. Their use therefore minimizes potential ecological risks in agricultural systems. The loaded antibiotic, zhongshengmycin, is a microbial secondary metabolite produced by *Streptomyces violaceusniger* and has been widely applied in China for the control of bacterial diseases such as bacterial wilt^67^. It has a low propensity to induce environmental resistance or ecological harm^6^. To date, there is no clear evidence linking its application to soil microbial imbalance or major ecological disturbance. In contrast, conventional antibiotics used in agriculture, including aminoglycosides, tetracyclines, and quinolones, have been widely reported to disrupt soil microbial communities^68^. These compounds can enrich target-specific ARGs and promote co-selection of resistance determinants, thereby accelerating the spread of multidrug resistance^68^. For example, oxytetracycline can increase the abundance of tetracycline resistance genes (e.g., *tetA*, *tetL*, *tetM*, *tetQ*, and *tetW*) by 10- to 1000-fold even at low exposure levels (2–70 µg/kg), highlighting its strong selective pressure on the soil resistome^69^. Such effects were not observed in the present study following the application of the zhongshengmycin-loaded CMA and CTA DN gels. Zn^2+^ is an essential micronutrient for plant growth and immunity^55^, but excessive application may lead to phytotoxicity or environmental risks. In this study, no ecological destabilization was observed after seven months. This is likely due to the controlled Zn^2+^ dosage and the pH-responsive release behavior of the DN hydrogels, which helps prevent excessive zinc accumulation. It should be noted, however, that the current assessment is based on a single application. Longer-term monitoring and repeated application studies of the CMA and CTA DN gels will be necessary to fully evaluate potential cumulative effects.

### CMA/CTA DN gels boost tomato resistance via defense pathways

**Fig. 9:**
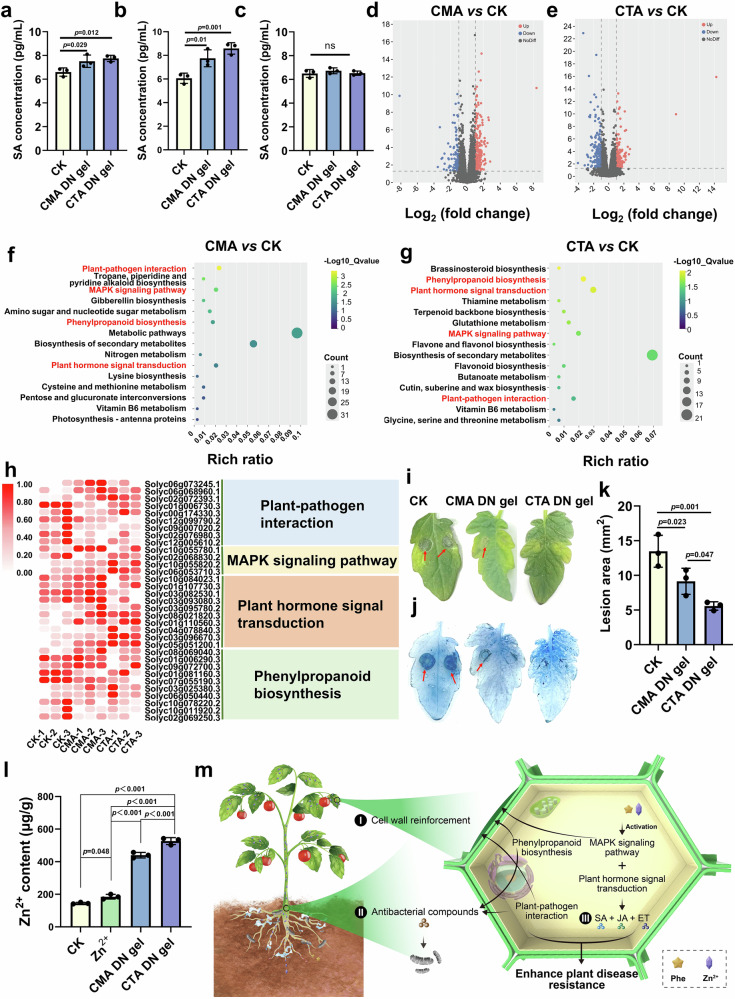
CMA/CTA DN gels induce systemic resistance in tomato plants. **a**–**c** Quantitative analysis of SA content in tomato leaves on days 3 (**a**), 5 (**b**), and 7 (**c**) following root application of the pesticide-free CMA or CTA DN gels. For panels **a**–**c**, all measurements were performed with 4 technical replicates and 3 biological replicates for each group. Data are presented as mean ± SD (*n* = 3 independent experiments). **d**, **e** Volcano plots showing differentially expressed genes in leaves from plants treated with **d** the CMA or **e** CTA DN gels compared to the control group (NoDiff stands for no difference). **f**, **g** KEGG pathway enrichment analysis of differentially expressed genes between **f** the CMA DN gel-treated group and the control (water treatment), and **g** the CTA DN gel-treated group and the control (the pathways associated with the plant immune responses for which the differentially expressed genes were predominantly enriched are highlighted in red). **h** Heatmap visualization of differentially expressed genes in leaves from plants treated with water (CK), the CMA DN gel, or CTA DN gel. Solyc is the abbreviation of the scientific name of the tomato (*Solanum lycopersicum*). **i** Representative images of tomato leaf disease symptoms (highlighted by red arrows) observed 8 days post-inoculation with *R. solanacearum*. **j** Trypan blue staining of infected leaves, with blue regions indicating dead cells caused by *R. solanacearum* infection. **k** Quantitative analysis of lesion areas among the treated groups. All measurements were performed with 12 technical replicates and 3 biological replicates for each group. Data are presented as mean ± SD (*n* = 3 independent experiments). **l** Zn^2+^ content in whole plants was measured 5 days after the treatment with water, a Zn^2+^ solution, the CMA DN gel, or CTA DN gel. All measurements were performed with 3 technical replicates and 3 biological replicates for each group. Data are presented as mean ± SD (*n* = 3 independent experiments). **m** Schematic representation of the CMA/CTA DN gel-mediated systemic resistance activation in tomato plants. JA and ET stand for jasmonic acid and ethylene, respectively. Statistical differences were determined using one-way ANOVA with a post hoc test.

In our previous research, we found that Zn^2+^ not only has antimicrobial properties but also activates the plant defense pathways, such as the salicylic acid (SA) pathway, to enhance the plant disease resistance^41,44^. Additionally, Phe was shown to be associated with the SA pathway^70^. In our pot experiments, the CMA/CTA DN gels demonstrated superior efficacy in controlling the bacterial wilt compared to the combined application of a conventional pesticide and Zn^2+^. This enhanced performance is likely attributable not only to the pH-responsiveness and sustained drug-release characteristics of the gels (Figs. 3i–n, 7e), which improve the bioavailability and stability of the pesticide against *R. solanacearum*, but also to the biological activity of the released Zn^2+^ and Phe. We hypothesize that these components may contribute to the activation of the plant disease-resistance signaling pathways, thereby augmenting the innate immune response of the host plant. To further elucidate the role of the CMA/CTA DN gels in plant defense, we quantified the SA levels in the treated plants at different time points (Fig. 9a–c). The analysis revealed that the plants treated with the CMA/CTA DN gels (without the encapsulated pesticide) exhibited significantly elevated SA concentrations compared to the control group (water) on days 3 (Fig. 9a) and 5 (Fig. 9b) post-application. However, no significant difference was observed on day 7 (Fig. 9c). This may be related to the release profile of Zn^2+^ and Phe (Fig. 3i, k, l, n), as the hydrogel releases most of these components during the first four days. Another reason is that plant hormone responses are transient, typically increasing within a few days after stimulation (e.g., by pesticides or nutrients) and then quickly returning to baseline levels^71^. These findings indicate that, beyond serving as pH-responsive delivery matrices for antimicrobial agents, the CMA/CTA DN gels may actively induce a plant defense response. This immunomodulatory effect is likely mediated by the sustained release of Zn^2+^ and Phe, which are known to participate in SA-associated signaling pathways^41,44,72^. Consequently, the gels may contribute to enhanced systemic resistance against phytopathogens such as *R. solanacearum*.

To gain deeper insights into the molecular mechanisms underlying the CMA/CTA DN gel-induced systemic resistance in plants, the tomato roots were treated with the pesticide-free CMA/CTA DN gels for 5 days, after which the leaf tissues were harvested for transcriptomic analysis via RNA sequencing. The comparative analysis revealed substantial changes in gene expression profiles relative to the control group (CK). Specifically, the treatment with the CMA DN gel resulted in the upregulation of 239 genes and downregulation of 103 genes (Fig. 9d), whereas the treatment with the CTA DN gel led to the upregulation of 139 genes and downregulation of 165 genes (Fig. 9e). These results demonstrate that both DN gels exert a pronounced impact on plant transcriptional regulation, suggesting their potential to modulate the key pathways involved in systemic immune responses. Furthermore, KEGG pathway enrichment analysis revealed that the differentially expressed genes were predominantly enriched in pathways associated with the plant immune responses, including phenylpropanoid biosynthesis, mitogen-activated protein kinase (MAPK) signaling, plant-pathogen interaction, and plant hormone signal transduction (Fig. 9f–h). The phenylpropanoid biosynthesis pathway is known to produce a range of secondary metabolites, such as lignin and flavonoids, which not only reinforce the plant cell walls to form a physical barrier against the pathogen ingress, but also serve as natural antimicrobial compounds that inhibit the pathogen proliferation^73,74^. The activation of the MAPK signaling cascade contributes to multiple immune responses, including the generation of reactive oxygen species (ROS), the transcriptional activation of defense-related genes, and the initiation of programmed cell death-mechanisms that are pivotal in limiting pathogen spread^75,76^. The plant-pathogen interaction pathway encompasses two fundamental immune strategies: i) the pattern-triggered immunity, which is initiated upon recognition of pathogen-associated molecular patterns, and ii) the effector-triggered immunity, activated by the detection of pathogen-derived effector proteins^77^. Both strategies orchestrate downstream defense events, including ion flux alterations, ROS bursts, cell wall fortification, and hypersensitive response, collectively enhancing the resistance against pathogen invasion^77^. Moreover, the plant hormone signaling pathways, particularly those involving SA, jasmonic acid, and ethylene, are essential regulators of the defense gene expression and immune activation^78,79^. These hormones modulate distinct but interconnected defense networks, contributing to tailored immune responses depending on the nature of the invading pathogen. Taken together, the RNA sequencing data suggest that the CMA/CTA DN gels robustly activate multiple immune-related signaling pathways, thereby promoting systemic acquired resistance and enhancing the overall capacity of the plant to counteract pathogenic infections.

To validate the reliability of the RNA-seq data, eight representative differentially expressed genes were selected for qPCR analysis (Supplementary Fig. 40). Among them, four genes, calcium-binding protein, *CBP3* (Solyc06g068960.1), ethylene receptor, *ETR4* (Solyc06g053710.3), ethylene-responsive transcription factor, *ERF1* (Solyc05g051200.1), and endochitinase 3-like (Solyc10g055820.2), were consistently upregulated (Supplementary Fig. 40a), whereas four genes, calcium-dependent protein kinase, *CDPK17-like* (Solyc12g099790.2), cysteine protease, *CYP* (Solyc02g076980.3), β-glucosidase 11 (Solyc01g081160.3), and cinnamyl alcohol dehydrogenase, *CAD6-like* (Solyc02g069250.3), were downregulated (Supplementary Fig. 40b). The qPCR results showed strong agreement with the RNA-seq data, thereby confirming the robustness and reliability of the transcriptomic dataset (Supplementary Fig. 40c, d). Given this validation, we next focused on key signaling pathways associated with plant immunity. Calcium signaling represents an early and central component of plant defense responses. Upon pathogen perception, transient cytosolic Ca^2+^ elevations are decoded by diverse Ca^2+^ sensors, including calmodulin (CaM)/calmodulin-like proteins (CMLs), calcineurin B-like proteins (CBLs), and calcium-dependent protein kinases (CDPKs), which subsequently regulate downstream defense processes^80^. Heatmap analysis revealed that the hydrogel treatment induced coordinated yet differential transcriptional changes in calcium signaling-related genes. Specifically, calmodulin-like protein 3 (Solyc06g073245.1) and *CBP3* (Solyc06g068960.1) were upregulated, and two leucine-rich repeat (LRR) receptor-like kinase-related genes (Solyc02g072393.1 and Solyc02g068830.2) also showed relatively high expression levels (Fig. 9h). In contrast, *CDPK20-like* (Solyc01g006730.3) and *CDPK17-like* (Solyc12g099790.2) were generally downregulated. This coexistence of upregulated and downregulated components suggests a selective reprogramming of the Ca^2+^ signaling network rather than a uniform activation or suppression, which is consistent with the dynamic and functionally specialized roles of different Ca^2+^ sensors in plant immunity^81^. In parallel, ethylene signaling, an essential regulator of plant immune responses, was also significantly affected. Heatmap analysis indicated that several ethylene signaling- and defense-related genes were upregulated following hydrogel treatment. Notably, *ETR4* (Solyc06g053710.3), *ERF1* (Solyc05g051200.1), endochitinase 4-like (Solyc10g055780.1), and endochitinase 3-like (Solyc10g055820.2) exhibited pronounced induction (Fig. 9h). Importantly, the magnitude of induction was generally higher in the CTA DN gel-treated group compared to the CMA DN gel-treated group, suggesting a stronger activation of the ethylene-associated defense pathway. Previous studies have demonstrated that ethylene signaling enhances tomato resistance to bacterial wilt by activating transcription factors and sustaining pathogenesis-related gene expression^82^. Collectively, these results indicate that the enhanced disease control efficacy of the CTA DN gel relative to the CMA DN gel may be, at least in part, attributed to its stronger stimulation of ethylene-mediated defense responses.

The activation of the systemic resistance is a key strategy by which plants enhance their overall capacity to defend against pathogenic microorganisms. The RNA sequencing analysis confirmed that the CMA/CTA DN gels significantly induced systemic resistance in tomato plants. Notably, despite being applied exclusively to the root zone, the DN gels conferred enhanced disease resistance in distal tissues, including the leaves. To validate this, the tomato roots were treated with the pesticide-free CMA/CTA DN gels, followed by foliar inoculation with *R. solanacearum* 5 days later. As shown in Fig. 9i, j, at 8 dpi, the leaves from the control group (treated with water) exhibited prominent necrotic lesions typical of bacterial infection. In contrast, the plants treated with the CMA DN gel or CTA DN gel developed only small necrotic spots, which were significantly reduced in size relative to the control (Fig. 9k), further substantiating the systemic resistance induced by the gels. Interestingly, the leaves from the CTA DN gel-treated group displayed slightly smaller lesions than those treated with the CMA DN gel. This difference may stem from the polycarboxylate structure of CTA, which exhibits higher acid sensitivity, thereby facilitating the release of higher quantities of active components (Phe and Zn^2+^) under mild acidic conditions (Fig. 3i, k, l, n). To explore this hypothesis, we quantified the Zn^2+^ content in tomato tissues following the treatment with either the CMA DN or CTA DN gel. The results indicated that the accumulation of Zn^2+^ was markedly higher in both DN gel-treated groups compared to the control groups (Zn^2+^ solution or water) (Fig. 9l). Moreover, the Zn^2+^ level was significantly greater in the CTA DN gel-treated group than in the CMA group, corroborating our assumption that the enhanced Zn^2+^ release may underlie the superior resistance observed in CTA-treated plants.

In summary, our findings demonstrate that the DN hydrogels composed of CMA and CTA allow a controlled release of Zn^2+^ and Phe, which collectively contribute to the activation of systemic resistance in plants, thereby enhancing their defense capacity against *R. solanacearum* infection (Fig. 9m). Importantly, the RNA sequencing analysis revealed that the upregulated signaling pathways are not restricted to bacterial-specific immune responses, but are broadly implicated in defense mechanisms against diverse classes of pathogens, including viruses and fungi. These results suggest that the systemic resistance elicited by the CMA/CTA DN gels may offer broad-spectrum protection, extending beyond bacterial diseases.

Overall, bacterial wilt remains a major challenge in global agriculture due to the lack of effective control strategies. Although some studies developed pH-responsive metal-organic framework-, nanoparticle and hydrogel-based drug delivery systems with promising therapeutic potential against bacterial wilt, several critical aspects remain underexplored^18,20,21^. For example, current research has not addressed the influence of soil environmental factors, such as pH and metal ion concentrations, on the aggregation behavior of nanoparticles, which could markedly affect their stability and disease control efficacy under field conditions. Furthermore, despite the encouraging results observed under laboratory settings, there is a lack of field-based efficacy evaluations under complex agricultural environments, making it difficult to fully assess their practical applicability in real-world farming systems.

In this study, we have successfully developed a multifunctional hydrogel using a straightforward approach, employing Zn^2+^ as a crosslinking agent to facilitate the assembly of CMA or CTA polymer with Phe. The hydrogels exhibit a dual pH-responsive behavior, allowing them to specifically respond to the acidic conditions (pH <5) characteristic of bacterial wilt. This environmental cue triggers the controlled and sustained release of the incorporated pesticide, Zn^2+^, and Phe, thereby providing precise control over bacterial wilt while simultaneously activating systemic resistance in the plants, which enhances both their growth and production yield. Remarkably, a single application of these hydrogels in agricultural production enabled the sustained bacterial wilt suppression for over a month, significantly reducing the disease incidence and ensuring stable crop yields. Our findings offer an innovative solution for the precise management of bacterial wilt, with considerable potential to enhance the pesticide efficacy, reduce the production costs (*e.g*., labor), and mitigate the environmental impact associated with an improper pesticide application^83^. To fully evaluate the agricultural applicability of the CMA/CTA DN gels, further extensive field trials across diverse regions and climatic conditions would be necessary to comprehensively assess their practical suitability. In addition, a thorough evaluation of the efficacy of the CMA and CTA DN gels against other plant diseases, such as viral infections, would help to demonstrate their broad-spectrum potential and further facilitate their practical implementation in agricultural production.

When comparing the efficacy of the two types of hydrogels (CMA and CTA DN gels), we observed that the CTA DN gel was better suited for agricultural applications. Both hydrogels demonstrated robust antibacterial activity under controlled laboratory conditions. However, the CTA DN gel exhibited superior pH-responsive controlled-release performance, enhanced mechanical properties (Supplementary Table 10), and a stronger ability to induce systemic resistance in plants. Notably, it is noteworthy that the degradation of the CTA DN gel may release citric acid, which could potentially promote plant growth^84^. Additionally, from a material synthesis perspective, CTA offers a simpler and more environmentally sustainable synthesis process compared to CMA, eliminating the need for toxic or environmentally harmful reagents. This would not only reduce the production costs, but also aligns more closely with the principles of sustainable agriculture.

## Method

### Chemicals and biological reagents

Agarose, zincon monosodium salt, and rhodamine B (97%) were purchased from Sigma-Aldrich. Chloroacetic acid and sodium hydroxide were obtained from Fisher Chemical. Citric acid was purchased from VWR chemicals. Phe was obtained from NeoMPS PolyPeptide Group. Zinc chloride was purchased from Fluka chemicals. Isopropyl alcohol was obtained from Honeywell. 12% Zhongshengmycin powder was kindly provided by Kaili Bioproducts Co., Ltd., Fujian, China.

The following kits were utilized according to the manufacturers’ instructions: RNA Extraction (LS1040, Promega Biotechnology), Reverse Transcription (RR037A, Shanghai Titan Technology), SYBR Green PCR (208252, QuantiNova™, Chongqing Shuguang Biotechnology), and salicylic acid ELISA (48 T, Tianjin Ruichuang Biotechnology Co., Ltd.).

### Synthesis of CMA and CTA

CMA was prepared according to a reported procedure^33^. Agarose (5 g) was suspended in 50 mL of isopropyl alcohol under continuous stirring at 80 °C for 30 min. The pH of the agarose solution was adjusted to 10 using 1.33 M NaOH solution, and the mixture was stirred for an additional 30 min. Subsequently, chloroacetic acid (2.5 g) was added, and the pH was maintained at 10 using 1.33 M NaOH. The reaction was allowed to proceed for 6 h. The reaction mixture was then precipitated by adding 100 mL of ethanol. The precipitate was collected by filtration, washed three times with ethanol, and dissolved in water. The pH of the aqueous solution was adjusted to 2 using hydrochloric acid (1 M), followed by dialysis against Milli-Q^®^ water for 3 days. Finally, the dialyzed solution was freeze-dried to obtain CMA as a powder (4.4 g, yield: 88%).

CTA was prepared by modifying the method of Jiang et al.^34^. Citric acid (4 g) was dissolved in 75 mL of Milli-Q^®^ water, and the pH was adjusted to 4 with NaOH (10 M). The solution was then diluted to 80 mL with Milli-Q^®^ water. This solution was mixed with agarose (20 g) and left to stand for 24 h at room temperature. The mixture was then dried at 35 °C for 24 h, ground, and heated at 120 °C for 24 h. After cooling, the mixture was filtered and washed with Milli-Q^®^ water to remove excess citric acid and dried in an oven at 40 °C for 48 h, yielding CTA as a powder (17.2 g, yield: 86%).

### Preparation of the CMA/CTA gels and CMA/CTA DN gels

#### CMA/CTA gel

A solution of CMA or CTA (0.5 g) in 100 mL of Milli-Q^®^ water was heated at 90 °C until fully dissolved. After cooling the solution to room temperature, an aqueous solution of ZnCl_2_ (50 μL, 0.24 M) was added to 1 mL of the prepared CMA/CTA solution (0.5% w/v) to reach a final Zn^2+^ concentration of 12 mM. The mixture was stirred thoroughly to ensure homogeneity and then allowed to stand undisturbed until the gel formation occurred.

#### Phe-Zn^2+^ gel

Phe (130 mg) was dissolved in 10 mL of Milli-Q^®^ water, and the pH was adjusted to 8.5 using 32% ammonia solution. 1 mL of prepared amino acid solution (78 mM) was then mixed with an aqueous solution of ZnCl_2_ (50 μL, 1.6 M) to reach a final Zn^2+^ concentration of 80 mM. The mixture was left to rest, allowing a white gel to form. To assess the acid-responsiveness of the Phe-Zn^2+^ gel, an aqueous solution of rhodamine B (50 μL, 1 mg/mL) was added to the hydrogel formulation. The hydrogel was then soaked in a HCl (1 M) solution or in aqueous solutions with different pH values (3, 5, and 7), observing the color changes and fluorescence intensity using a UV lamp (365 nm) of the released solutions.

#### CMA/CTA DN gels

A CMA or CTA (1% w/v) solution (500 μL) prepared following the above procedure was pre-cooled to room temperature and first mixed with an aqueous solution of ZnCl_2_ (50 μL, 0.24 M). An equal volume (500 μL) of an aqueous solution of Phe (78 mM) was then promptly added, followed by thorough mixing. The resulting mixture was allowed to stand undisturbed for 3 min to form the CMA or CTA DN gels, thereby yielding the CMA or CTA DN gel within 3 min. For the zhongshengmycin-loaded CMA or CTA DN gels, zhongshengmycin (100 μL, 40 mg/mL) was first added to the CMA/CTA solution after it had been cooled to room temperature. This solution was then mixed with ZnCl_2_ and Phe, following the procedure described above.

### Characterization of the CMA/CTA and DN gels

Fourier transform infrared (FTIR) spectroscopy was performed using a PerkinElmer Frontier™ spectrometer to acquire spectra within the 4000–400 cm^−1^ range, utilizing 64 scans as per the instrument’s standard setup. Surface charge measurements of CMA/CTA were conducted with a Zetasizer (Malvern Panalytical). For NMR spectroscopy analysis, CMA and CTA were dissolved in DMSO-d_6_, followed by the recording of their ^1^H and ^13^C NMR spectra using a Bruker Avance III 500 MHz spectrometer. The DN structure of the CMA DN gels and CTA DN gels was examined using cryo-SEM (Regulus8220, Japan). The internal microstructure of the CMA/CTA DN gels, after immersion in solutions at pH 7 and pH 3 for 7 days, was visualized using transmission electron microscopy (TEM, Hitachi High Technologies Corporation, Japan) and SEM (SU8020, Hitachi). The rheological properties were assessed through time sweep (0.5% constant shearing stress, 1 rad/s angular frequency) and frequency sweep (at a constant oscillatory stress of 1 Pa) experiments conducted with a rheometer (Anton Paar MCR 702 e).

### pH-responsive drug release experiments

The zhongshengmycin (2 mg/mL)-loaded CMA/CTA DN gels were immersed in 10 mL of solutions with pH values of 3, 5, and 7, respectively. After 0, 12, 24, 48, 96, 120, 144, and 168 h of immersion, 100 μL of the release solution was collected to determine the concentrations of the pesticide, Zn^2+^, and Phe. The concentrations of the pesticide and Phe were calculated using high-performance liquid chromatography (HPLC, Nucleosil 100-5 Waters C18 reverse-phase HPLC column and Waters Alliance e2695 separation module). The amount of Zn^2+^ was assessed using zincon monosodium salt as a chromogenic reagent. The blue colored Zn^2+^-zincon complex was analyzed by UV-Vis spectrophotometry (Thermo Scientific, Varioskan Lux) at 618 nm.

### In vitro antibacterial activity assay

Both *R. solanacearum* and *R. solanacearum* carrying green fluorescent protein (*R. solanacearum*-GFP) were obtained from Prof. Wang’s laboratory at the College of Resources and Environment, Southwest University, China. The in vitro antibacterial activity of the CMA/CTA DN gel was evaluated through bacterial growth curve measurements and colony counting experiments.

First, the antibacterial effects of Zn^2+^ and the pesticide were assessed. Different concentrations of Zn^2+^ (5, 10, 20, 40 μg/mL) and the pesticide (10, 20, 40, 80 μg/mL) were added to Luria-Bertani (LB) medium. For each group, 198 μL of medium was distributed into 96-well plates, with 2 μL of *R. solanacearum* suspension (10^9^ CFU) added to each well. The plates were sealed and incubated at 30 °C, and the optical density at 600 nm (OD_600_) was measured at 0, 2, 4, 12, 24, and 48 h.

The DN gels (with a pesticide concentration of 2 mg/mL) were soaked in solutions of different pH (3, 5, and 7) for 168 h. The release solution was collected, and the pH was adjusted to 7 using an aqueous solution of NaOH. A volume of 20 μL of the release solution was added to 178 μL of LB medium, transferred to 96-well plates, and mixed with 2 μL of *R. solanacearum* or *R. solanacearum*-GFP (10^9^ CFU). The cultures were incubated at 30 °C, and OD_600_ values were recorded at 0, 2, 4, 12, 24, and 48 h. Zn^2+^, the pesticide, and pesticide+Zn^2+^ mixture were used as positive controls, while untreated LB medium served as the negative control. All experiments were performed with at least three replicates and repeated three times. Additionally, for *R. solanacearum*-GFP treated with the DN gels, confocal microscopy (Leica, stellaris5) was performed 24 h after treatment to observe fluorescence signals (excitation at 488 nm, emission at 498–570 nm) to assess bacterial proliferation.

For colony counting experiments, the grouping and treatment concentrations were the same as described above. Zn^2+^, the pesticide, pesticide+Zn^2+^ mixture, and 20 mL of the DN gel release solutions were incorporated into 180 mL of the agar-containing LB medium to prepare drug-containing media. After the medium solidified, 50 μL of *R. solanacearum* (10^4^ CFU) was spread on the surface and incubated at 30 °C for three days. The number of colonies was counted, and the inhibition rate was calculated using Eq. (1)^85^.

1 $${{{\rm{Inhibition}}}}\,{{{\rm{rate}}}}(\%)=\frac{{{{\rm{N}}}}-{{{\rm{n}}}}}{{{{\rm{N}}}}}\times 100\%$$

In this equation, N represents the number of bacterial colonies observed in the control group, while n denotes the number of bacterial colonies recorded for the group exposed to the tested materials.

### Live/dead assay and bacterial morphology observation

The bacterial viability was evaluated using the live/dead staining method^86^, and the bacterial structure was observed by SEM^20^. Specifically, freshly cultured bacteria (10^8^ CFU/mL) were mixed with the CMA/CTA DN gel release solution and incubated at 30 °C for 4 h. Afterward, the bacterial suspension was centrifuged at 1789 × *g* for 5 min to collect the bacterial precipitate and then resuspended in distilled water.

#### Live/dead cell assay

1 mL of bacterial suspension was stained with 1 μL of 10 mM propidium iodide solution and 1 μL of 3 mM SYTO9 solution at room temperature in the dark for 30 min. Subsequently, 10 μL of the stained suspension was dropped onto a glass slide and examined using a confocal laser scanning microscope. The fluorescence settings were as follows: excitation at 488 nm with emission at 498–560 nm for the green channel (live bacteria), and excitation at 561 nm with emission at 571–630 nm for the red channel (dead bacteria).

#### Bacterial cell structure observation

Bacteria were fixed in 2.5% glutaraldehyde and then dehydrated sequentially using ethanol solutions with concentrations of 30%, 50%, 70%, 90%, and 100%. Following dehydration, the samples were sputter-coated with gold and examined for morphological changes using SEM.

### In vivo antibacterial activity assay

The potted plant experiment was conducted to evaluate the effectiveness of the CMA/CTA DN gels in controlling bacterial wilt. Healthy 5-week-old tomato plants (*Lycopersicon esculentum*, cultivar DRK0568), grown in soil with a pH of 5.0 ± 0.3, were selected and treated with fresh bacterial suspensions of *R. solanacearum* or *R. solanacearum*-GFP (5 mL, OD_600_ = 0.2) by root irrigation. Subsequently, 2 mL of the CMA/CTA DN gel was buried 2 cm below the soil surface, and the plants were incubated in a growth chamber (30 °C, 12 h light, 12 h dark, 60% humidity) for observation. The control groups were treated with distilled water, a Zn^2+^ solution (15 μg/mL), a pesticide solution (40 μg/mL), and a mixture of pesticide+Zn^2+^solution (Zn^2+^: 15 μg/mL, pesticide: 40 μg/mL). The disease symptoms were observed at day 7 and 14 after root irrigation, and the disease index was calculated using Table 1 and Eq. (2). The root and near-root stem sections of the plants treated with *R. solanacearum*-GFP were observed using a confocal microscope to assess bacterial infection (excitation at 488 nm, emission at 498–570 nm) in the tomato plants. The resulting fluorescence images were quantitatively analyzed using ImageJ (version 6.0).

2 $${{{\rm{D}}}}{{{\rm{isease}}}}\,{{{\rm{index}}}}=\frac{\sum N1\times G}{N\times {G}_{max }}{{{\rm{x}}}}100$$

**Table 1:** Grading criteria for *R. solanacearum* infection in tomato

Level	Degree of disease
0	no wilt
1	0–25% of leaves wilted
2	26–50% of leaves wilted
3	51–75% of leaves wilted
4	76–100% of leaves wilted

The total number of leaves at each grade, denoted as N1, is determined by counting the leaves found at each designated grade level (Table 1). The variable G represents the number of leaves at a specific grade, while N stands for the total number of leaves across all levels. G_max_ refers to the maximum value representing the highest grade level.

### Quantitative real-time PCR (qPCR)

To evaluate the extent of *R. solanacearum* infection in the roots under different treatments, tomato roots from each treated group were collected. After a thorough washing, the load of *R. solanacearum* was quantified using qPCR. Total RNA from the samples was extracted using Trizol Plus reagent (Takara, Cat. No. 9108/9109), and cDNA synthesis was performed with a reverse transcription kit (Takara, RR037A). qPCR detection was carried out using a thermal cycler (CFX Touch Real-time PCR, BioRad) and SYBR Prime qPCR kit (Bioground, BG0014). Actin, a highly conserved housekeeping gene, was used as the internal reference for normalization. Relative changes in gene transcription levels were calculated using the 2^−^^ΔΔCT^ method^30^, with each sample analyzed in triplicate. Supplementary Table 11 provides the primer sequences used for qPCR analysis.

### ELISA testing

The SA content in each treated group was measured using an SA ELISA kit. Following the manufacturer’s instructions, leaf samples (0.2 g) were first ground in PBS buffer and then centrifuged to collect the supernatant. Next, the sample was mixed with horseradish peroxidase-labeled detection antibody (100 μL) and incubated at 37 °C for 60 min in the water bath. After incubation, the reaction solution was discarded, and the wells were washed five times with wash buffer. Substrate solutions (A, B) were then added, and the plate was incubated at 37 °C in the dark for 15 min. Finally, the reaction was stopped by adding the stop solution, and the absorbance (OD_450_) of each well was measured using a microplate reader (Thermoscientific, Varioskan LUX).

### Field experiments

The field experiments were conducted from June to August in both 2024 and 2025, at the Runyu Agricultural Planting Base in Shuanghe Street, Rongchang District, Chongqing, China (coordinates: 29.3151011, 105.5686133). The average daily temperature during the field experiment was 33.6 ± 3.8 °C (Supplementary Table 2 and Supplementary Table 3, data sourced from the China Meteorological Administration). The base features a fixed 5-acre plot for tomato cultivation, which has been continuously farmed for over five years and experiences recurring bacterial wilt annually. Five randomly selected greenhouses were used for the field trial, with the following treatment groups: water control, pesticide, CMA DN gel, and CTA DN gel, each containing 30–40 tomato plants. In the experiment, 8-week-old tomatoes (flowering stage) were treated with 10 mL of the gel (pesticide concentration: 2 mg/mL) at approximately 4 cm (near the tomato roots) below the soil surface. The pesticide group received 500 mL of pesticide (concentration: 40 μg/mL) solution. Irrigation was carried out twice a week for each group via drip irrigation. Only one application of the hydrogel or the pesticide was performed throughout the entire experiment, with no additional pesticides or fertilizers used. Data collection began once bacterial wilt symptoms appeared in the control group, and data were recorded weekly, totaling four measurements. At the end of the experiment, tomato plant height and width, and fruit yield were measured. Additionally, during the experiment, soil samples were randomly collected from the control and symptomatic treated groups, and pH testing was conducted to assess the soil acidity associated with the bacterial wilt occurrence.

Soil samples were collected on January 20, 2026, from field plots established in 2025 and subsequently submitted to Majorbio (Shanghai, China) for metagenomic sequencing (NCBI Bioproject accession number: PRJNA1445876) on the DNBSEQ-T7 platform. After completion of the 2025 field trial, the plots were maintained in a fallow state, with no crop cultivation or agronomic interventions conducted prior to sampling.

### RNA sequencing and analysis

The CMA and CTA DN gels were embedded into the soil surrounding the roots of tomato plants, and 10 mL of water was applied to the soil every 24 h. Plants treated with water alone served as the negative control. After 5 days of treatment, the leaves from the same position on each plant were collected and sent to Beijing Qingke Biotechnology Co., Ltd. (Beijing, China) for transcriptome sequencing using the Illumina HiSeq™ 2500 platform (NCBI Bioproject accession number: PRJNA1271282). Clean reads obtained from sequencing were aligned to the tomato reference genome (SL3.0.51) using RSEM software (version 1.3.1), and the expression levels of each unigene were calculated in terms of FPKM (Fragments Per Kilobase of transcript per Million mapped reads). Differentially expressed genes between each hydrogel-treated group and the control group were identified using NOIseq software (version 1.20.0). The criteria for differentially expressed gene selection were set as |log_2_(fold change)| ≥ 2 and false discovery rate (FDR) ≤ 0.05.

In addition, four upregulated genes and four downregulated genes identified from the transcriptome were selected for qPCR analysis, with primers listed in Supplementary Table 11.

### Statistical analysis

Experimental data were obtained in triplicate and are presented as mean values accompanied by their respective standard deviations. Statistical analyses for comparison among multiple groups were performed using one-way ANOVA, followed by Duncan’s, LSD’s or Tukey HSD post hoc test, with SPSS (version 26.0) software. *P* < *0.05* was considered to indicate statistical significance. Figure generation was performed using Origin 2021, GraphPad Prism 9.0.0, and Microsoft Excel 2016. The schematic diagrams in the manuscript were created using Illustrator and BioRender.

### Reporting summary

Further information on research design is available in the Nature Portfolio Reporting Summary linked to this article.

## Supplementary information

Supplementary Information

Description of Additional Supplementary Files

Supplementary Data 1

Supplementary Data 2

Reporting Summary

Transparent Peer Review file

## Source data

Source Data

## Data Availability

The soil metagenomic and tomato RNA sequencing data generated in this study have been deposited in the NCBI database under accession code PRJNA1445876 https://www.ncbi.nlm.nih.gov/search/all/?term=PRJNA1445876+, PRJNA1271282 https://www.ncbi.nlm.nih.gov/search/all/?term=PRJNA1271282. All other analyses and data generated in this study are provided in the Supplementary Information/Source Data file. Source data is available for Figs. 3i–n, 4a, b, e, 5c–e, 6b, c, 7c, d, 8e and 9a–c, k, l, Supplementary Figs. 3, 13, 14, 15a–d, 16, 17a, c, 22, 23, 24c–f, 25b–e, 26b–e, 27, 37b and 40 and Supplementary Tables 1, 4, 5, 6 and 10 in the associated source data file. The source data are provided with this article. Source data are provided with this paper.
