# Supplementary material for: A pH-responsive double network hydrogel for control of tomato bacterial wilt
Source: Nat Commun. 2026 Jun 12;17:5219. doi: 10.1038/s41467-026-73922-3 (PMC13263331; doi:10.1038/s41467-026-73922-3)
Supplement: Supplementary file 1 — Supplementary Information [file 41467_2026_73922_MOESM1_ESM.docx]

**A pH-Responsive Double Network Hydrogel for Control of Tomato Bacterial Wilt**

Shunyu Xiang^1,2^, Meijun Chen^2^, Shaorui Tian^2^, Haoran Peng^2^, Hulian Sun^2^, Mengji Cao^2^, Cécilia Ménard-Moyon^1,3^*, Xianchao Sun^2^*, Alberto Bianco^1^*

^1^CNRS, Immunology, Immunopathology and Therapeutic Chemistry, UPR 3572, University of Strasbourg, ISIS, 67000 Strasbourg, France

^2^College of Plant Protection, National Citrus Engineering Research Center, Citrus Research Institute, Southwest University, Chongqing 400715, China

^3^University of Strasbourg Institute of Advanced Study (USIAS), 67000 Strasbourg, France

Supplementary INFORMATON

**Calibration curve of the pesticide, Zn^2+^, and Phe**

To quantitatively assess the concentration of pesticide, Zn^2+^, and Phe released from the CMA/CTA DN gels, HPLC and UV-Vis spectrophotometry (Cary 5000, Agilent Technologies) were employed. Calibration curves were constructed by plotting the HPLC peak areas (pesticide and Phe) or the absorbance (Zn^2+^) values against known concentrations, with linear regression analysis performed using Microsoft Excel to calculate the coefficient of correlation (R^2^). For Zn^2+^ quantification, a colorimetric assay based on zincon monosodium salt was utilized. Specifically, a 1.6 mM zincon solution was prepared in water, followed by the addition of Zn^2+^ solutions at varying concentrations. The absorbance of the resulting blue-colored Zn^2+^-zincon complex was measured at 618 nm using a UV-Vis spectrophotometer.

**Simulating plant drug absorption using a dye**

To assess the uptake efficiency of compounds released from the hydrogels by plant roots and their subsequent translocation within the plant, 5(6)-carboxyfluorescein, a fluorescent tracer commonly used as a surrogate for small-molecule pesticides,^1^ was incorporated into the CMA/CTA DN gels at a concentration of 100 μg/mL using the same loading procedure as for the pesticide. The hydrogels were applied to the root zone of test plants, with supplemental watering administered directly at the root interface on days 0 and 3 following the treatment. On day 7, the root and stem tissues were collected, sectioned, and analyzed by confocal laser scanning microscopy to visualize and evaluate the presence and spatial distribution of green fluorescence.

**Plant growth**

To investigate the plant growth-promoting effects of the CMA/CTA DN gels, a series of growth assays were conducted using *Solanum lycopersicum cv. Micro-Tom* as the model plant. The solutions released from the gels were collected under various pH conditions and subsequently adjusted to a neutral pH (7.0) to eliminate possible pH-related interference. The solutions were applied via root irrigation on days 0, 5, 10, and 15. The treatment period was extended until the plants reached 7 weeks of age. Key growth parameters, including plant height, leaf width, root length, fresh weight, and dry weight, were recorded to assess developmental outcomes. Additionally, Zn^2+^ accumulation in roots, stems, and leaves was quantified using inductively coupled plasma mass spectrometry (ICP-MS, Agilent 7800). Plants irrigated with deionized water served as the control group to establish baseline physiological benchmarks.

**Seed germination**

To comprehensively assess the impact of the CMA/CTA DN gels on seed germination and evaluate their biosafety for potential agricultural application, germination assays were conducted using seeds from tomato (*Solanum lycopersicum*), rice (*Oryza sativa*), and maize (*Zea mays*) representing both horticultural and staple crop species. Viable, uniformly sized seeds were first soaked in deionized water for 24 h to initiate imbibition, then evenly distributed onto filter paper pre-saturated with the solutions released from the hydrogel. The treated seeds were incubated in a controlled environment chamber under dark conditions at 28 °C and 75% relative humidity for 7 days. Following the incubation, germination rates were recorded and the length of the emerging radicles was measured to evaluate early seedling vigor.

**Ecological safety assessment of the CMA/CTA DN gels on earthworms**

To evaluate the ecological safety of the CMA/CTA gels toward soil-dwelling organisms, *Enchytraeus crypticus*, a standard bioindicator species widely used in soil ecotoxicology, was selected for acute toxicity testing. The experimental protocol was adapted with modifications from the method established by Gomes *et al.*^2^ Specifically, the hydrogels were homogenously incorporated into natural soil at a concentration of 2 g/kg (dry weight basis). After a thorough mixing, twenty healthy and similarly sized adult *E. crypticus* individuals were introduced into each treatment group. The amended soil was then transferred into individual test containers and maintained under controlled environmental conditions (20 °C, constant temperature) for a 7-day exposure period. Throughout the experiment, worm activity and general health status were monitored at regular intervals. At the end of the exposure period, the number of surviving individuals in each treatment group was recorded.

**Pollen germination assay and fruit set analysis**

2 mL of the CMA/CTA DN gel was applied to 4-week-old tomato plants. At the onset of flowering, three flowers from the first inflorescence of each plant were collected for analysis. Pollen grains were carefully transferred onto clean glass slides, followed by the uniform addition of pollen germination medium (obtained from a commercial pollen germination kit). The prepared slides were placed in Petri dishes containing moistened filter paper to maintain a high-humidity environment and incubated at 25℃ for 2 h. Pollen germination was then examined under a light microscope (Nikon DS-Ri2, Japan), and the germination rate was quantified according to Equation (1). For fruit set assessment, the number of successfully developed fruits on the first inflorescence was recorded, and the fruit set rate was calculated based on Equation (2).

Pollen germination rate (%)=$\frac{N1}{N2}$ x100 (1)

*N*1 represents the total number of germinated pollen grains. *N*2 represents the total number of pollen grains.

Fruit set rate (%)=$\frac{N3}{N4}$ x100% (2)

*N*3 represents the number of fruits set. *N*4 represents the total number of flowers.

***R. solanacearum* infection of tomato leaves**

Five days after the CMA or CTA DN gel was applied to the root zone of tomato plants, *R. solanacearum* was inoculated into the tomato leaves using a syringe. A volume of 150 μL of the bacterial suspension (OD_600_= 0.1) was injected at each designated site on the leaves. At 7 dpi, the plants were evaluated for bacterial lesions on the leaves, and the lesion areas were quantified. Additionally, the affected leaves were collected and subjected to Trypan Blue staining to visualize and assess the extent of bacterial infection.^3^ After the staining procedure, dark blue regions indicated the presence of dead plant cells, serving as a marker of tissue damage and infection severity.


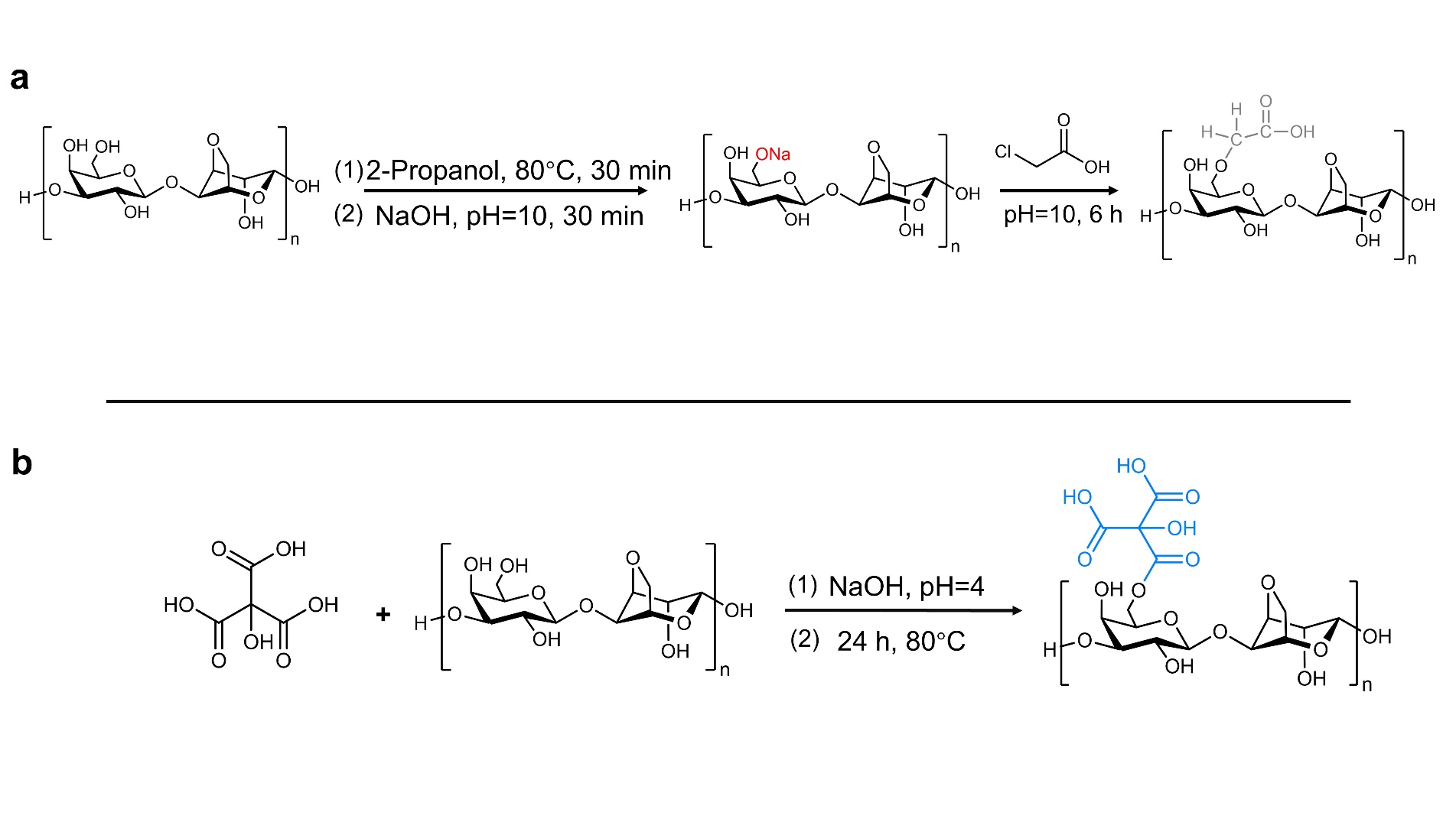


**Supplementary Figure 1:** (a) Synthesis of carboxymethylated agarose and (b) citrate agarose.


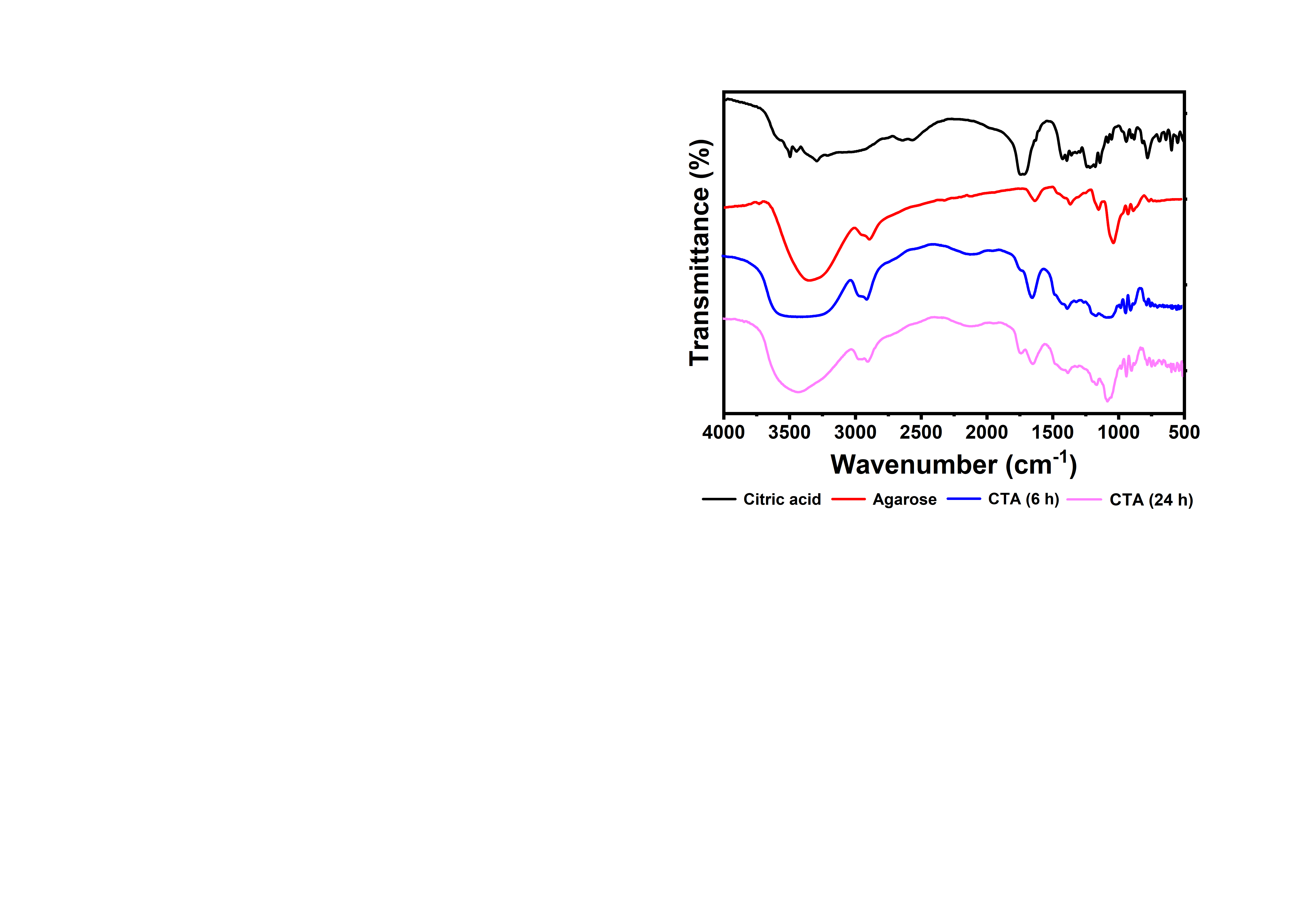


**Supplementary Figure 2:** FTIR spectra of citric acid, agarose, and CTA at different reaction times.

**Supplementary Figure 3:** Zeta potential of agarose, CMA, and CTA. All measurements were performed with 3 technical replicates for each group and the experiment was independently repeated 3 times. Data are presented as mean ± SD (n = 3 independent experiments).


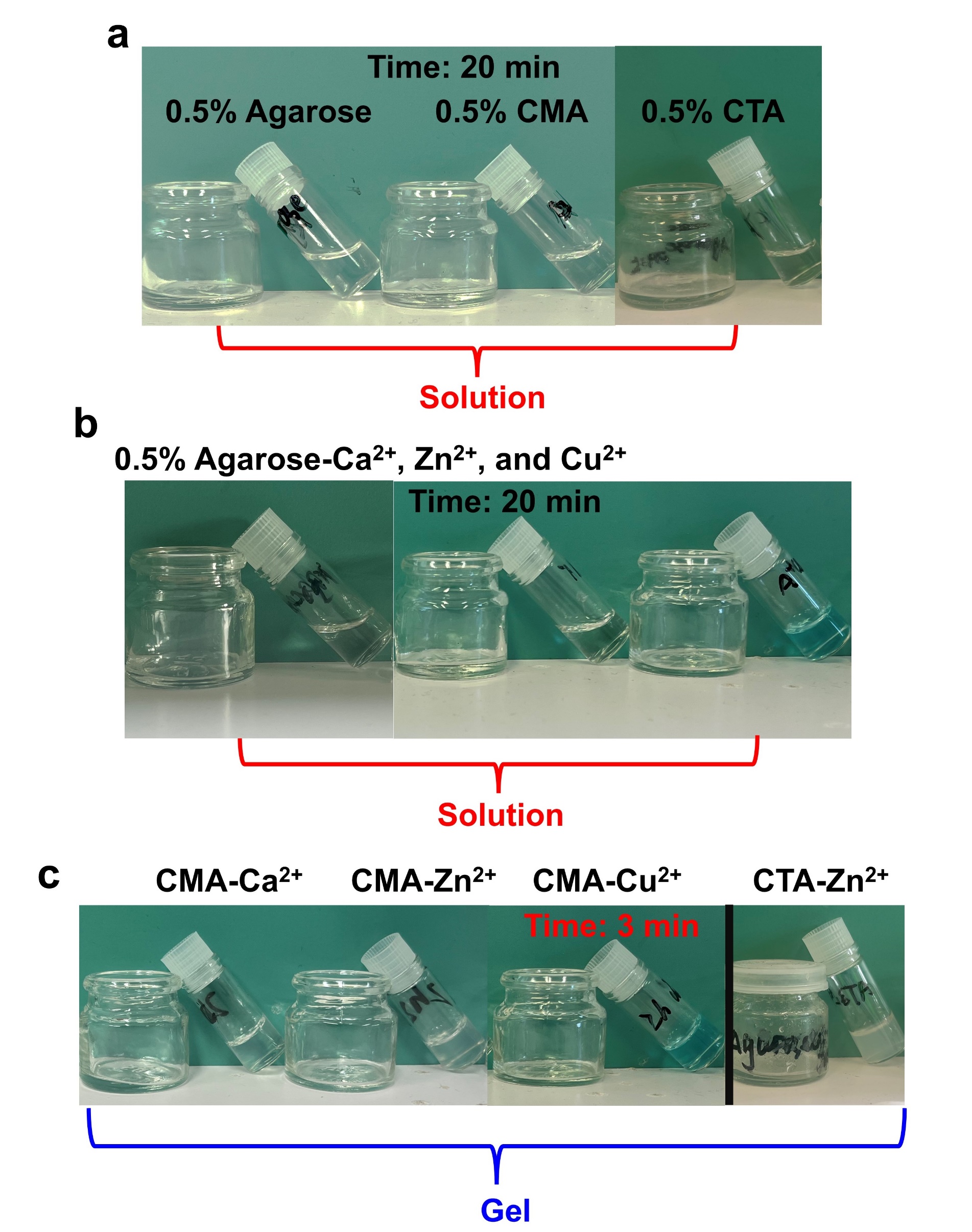


**Supplementary Figure 4:** (a) Photographs of 0.5% agarose, 0.5% CMA, and 0.5% CTA solution after standing at room temperature for 20 min. (b) Photographs of 0.5% agarose mixed with Ca^2+^, Zn^2+^, and Cu^2+^ after standing at room temperature for 20 min. (c) Photographs of 0.5% CMA-Ca^2+^, CMA-Zn^2+^, CMA-Cu^2+^, and 0.5% CTA-Zn^2+^ hydrogels after standing at room temperature for 3 min. In panel (a) and (b) the gelation time was longer than 20 min for all samples. Indeed, at the 20-min time point, most samples still appeared as solutions or were only in a weakly gelled state.


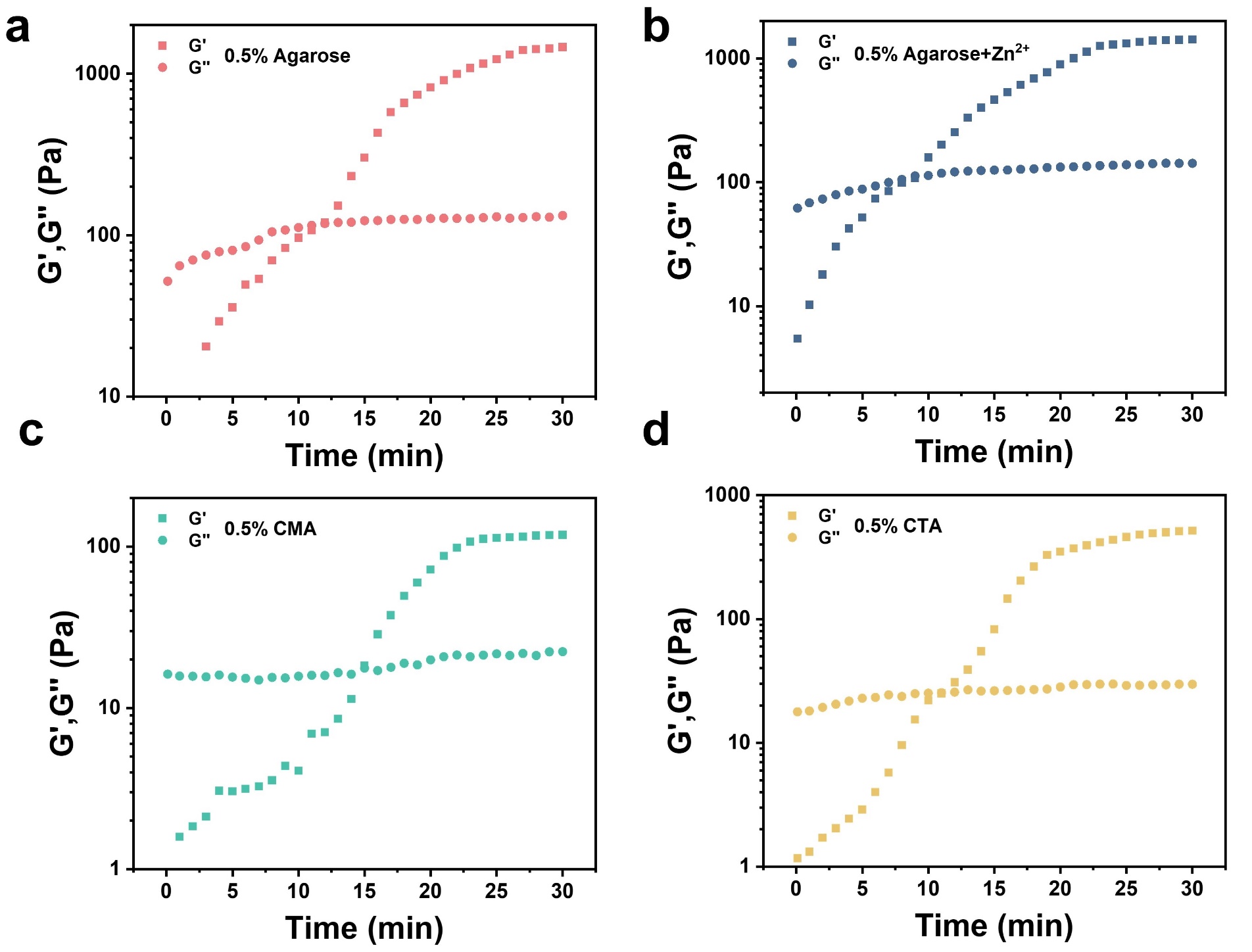


**Supplementary Figure 5:** Time-dependent progression of storage modulus (G') and loss modulus (G") for the following samples: (a) 0.5% agarose, (b) 0.5% agarose with Zn^2+^, (c) 0.5% CMA, and (d) 0.5% CTA.

**Detailed explanation of the data in Figure S5 and Figure S6:**

The gelation time for agarose, CMA, and CTA was approximately 10-15 min (**Supplementary Figure 5a**, **5c** and **5d**). However, the addition of Zn^2+^ led to immediate gel-like behavior (G' > G'') in CMA-Zn^2+^ and CTA-Zn^2+^ gels as the measurements started (**Figure 2f** and **2g**), whereas agarose-Zn^2+^ showed little reduction of the gelation time (**Supplementary Figure 5b**). Moreover, the frequency sweep tests were conducted to evaluate the impact of Zn^2+^ and Phe on the mechanical properties of the CMA/CTA gels (**Supplementary Figure 6**). The results indicated that the mechanical properties of agarose, CMA, and CTA increased significantly with increasing concentrations of agarose, CMA, and CTA, respectively (**Supplementary Figure 6a-c**). Notably, Zn^2+^ greatly enhanced the mechanical strength (G' increased by several folds) of the CMA/CTA gels at all tested concentrations, but had little effect on agarose. These results confirm that Zn^2+^ can be chelated by the carboxyl groups in CMA and CTA, thereby increasing the crosslinked network density and enhancing mechanical strength. Similarly, Phe-Zn^2+^ exhibited a concentration-dependent enhancement in the mechanical properties (**Supplementary Figure 6d**).


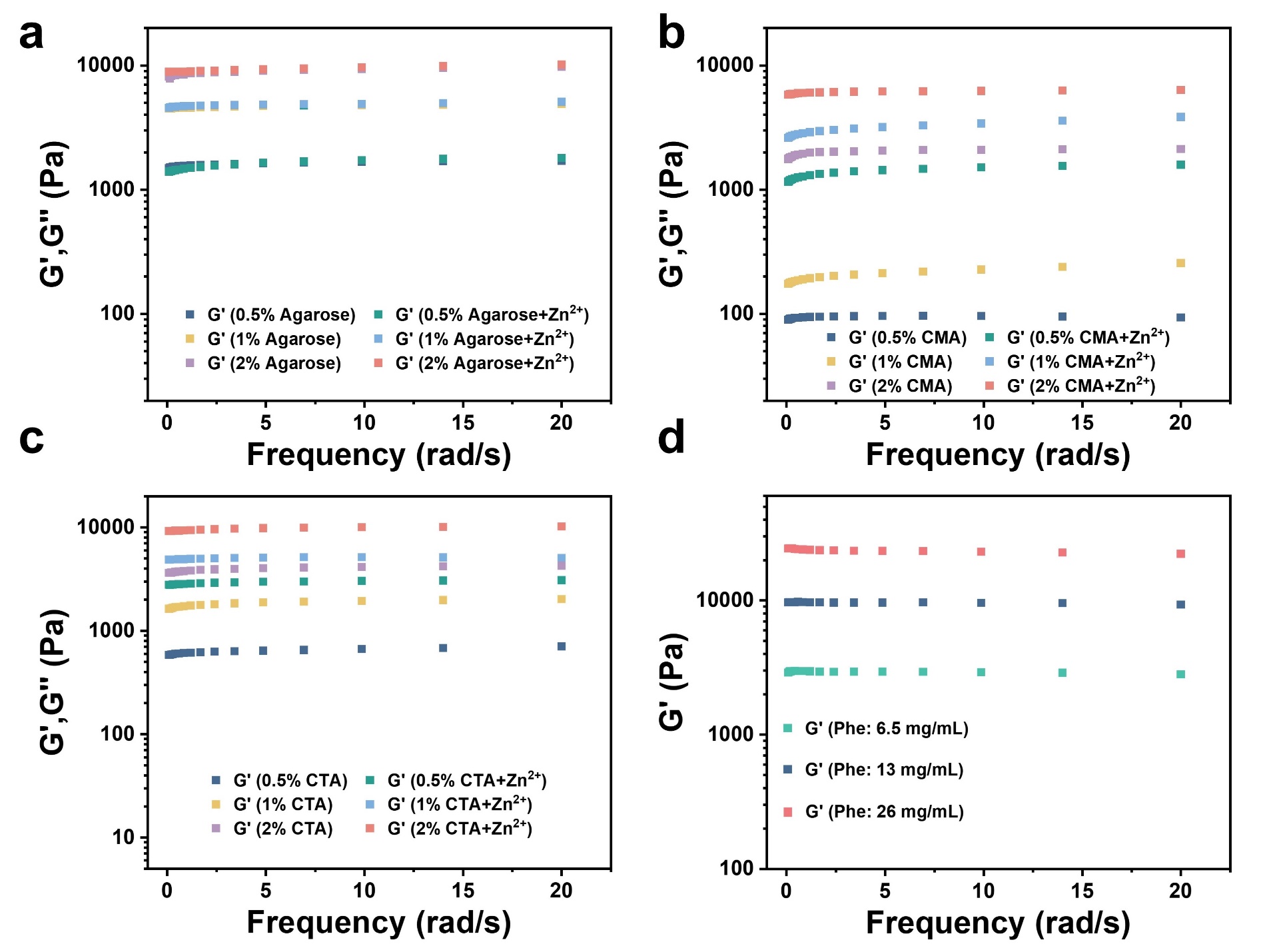


**Supplementary Figure 6:** Frequency sweep analysis of (a) agarose, (b) CMA, and (c) CTA hydrogels at different concentrations with and without Zn^2+^, and (d) Phe-Zn^2+^ hydrogel at varying Phe concentrations.


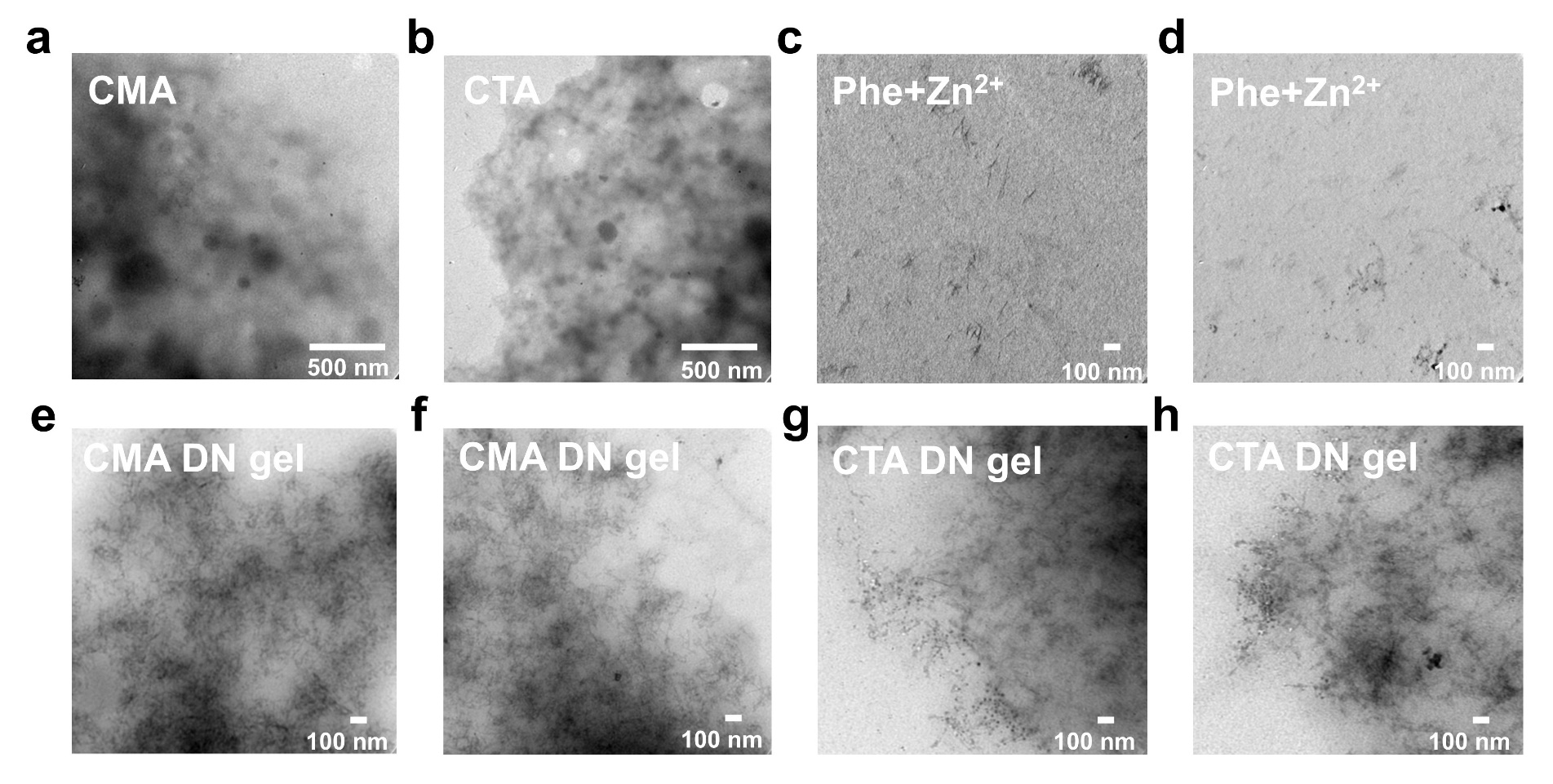


**Supplementary Figure 7:** TEM images of (a) CMA, (b) CTA, (c, d) Phe-Zn^2+^ gel, (e, f) CMA DN gel, and (g, h) CTA DN gel. All observations were independently repeated 3 times to ensure the robustness of the results.


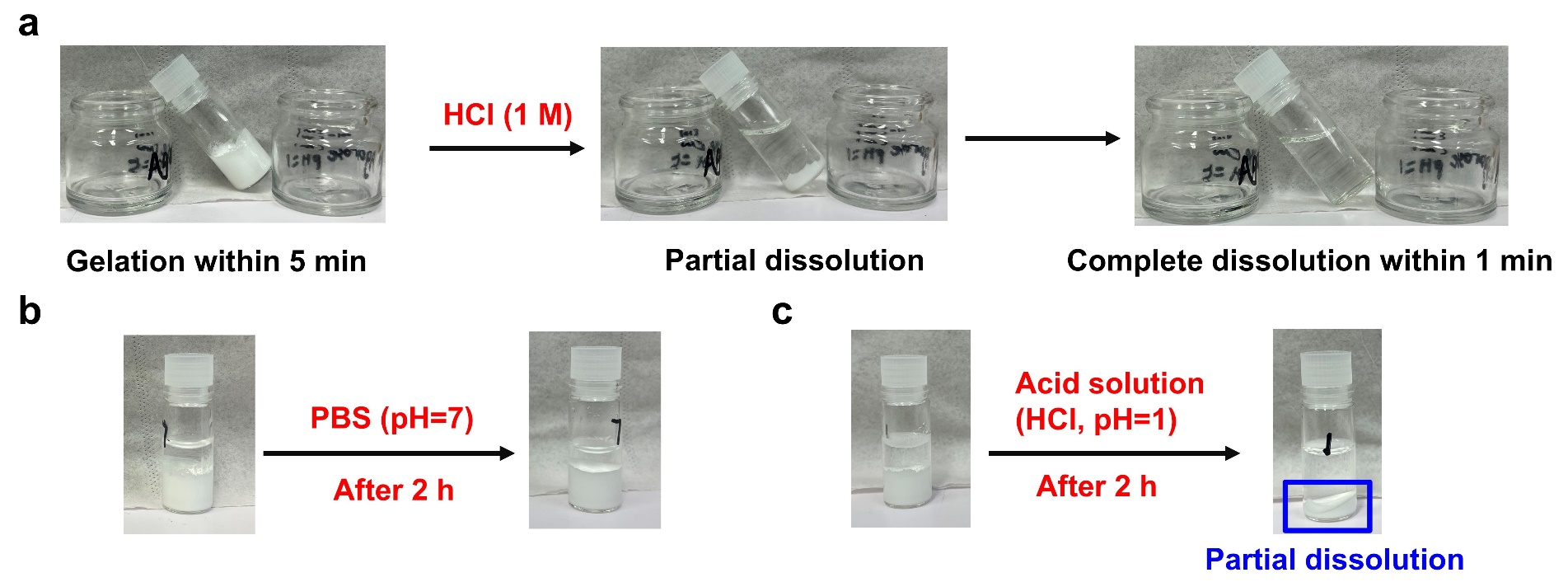


**Supplementary Figure 8:** (a) Dissolution state of Phe-Zn^2+^ gel in HCl solution (1 M), (b) neutral solution, and (c) pH 1 solution.


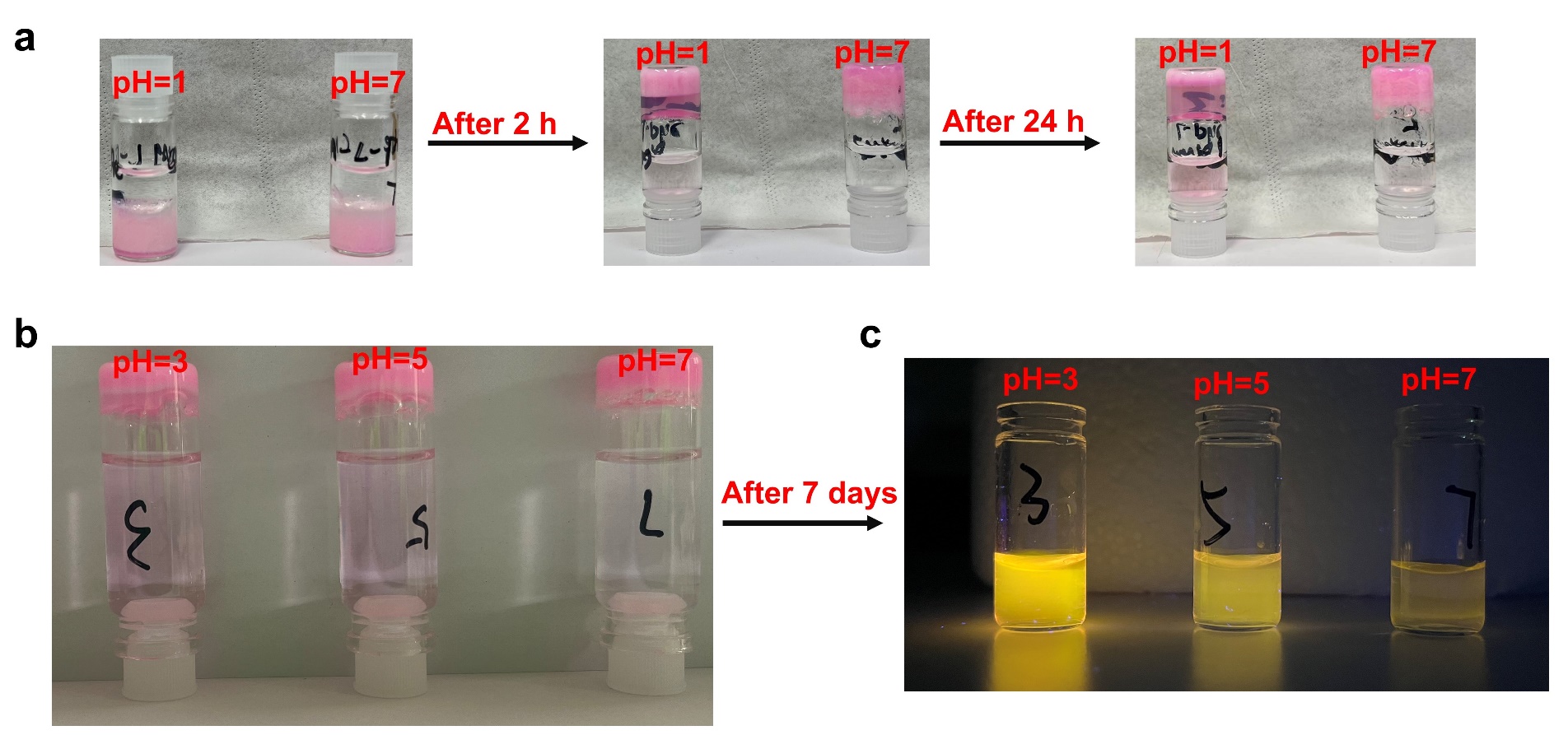


**Supplementary Figure 9:** (a) Representative photos showing the color of the solutions released from the CMA DN gel loaded with rhodamine B after 24-h immersion in solutions at pH  1 and pH  7. (b) Representative photos showing the color of the solutions released from the CMA DN gel loaded with rhodamine B after 7 days of immersion in solutions at pH  3, 5, and 7. A deeper color indicates a higher amount of rhodamine B released from the CMA DN gel. (c) Fluorescence photos (under UV light, 365 nm) of the solutions released from the CMA DN gel loaded with rhodamine B after 7 days of immersion in solutions at pH 3, 5, and 7.


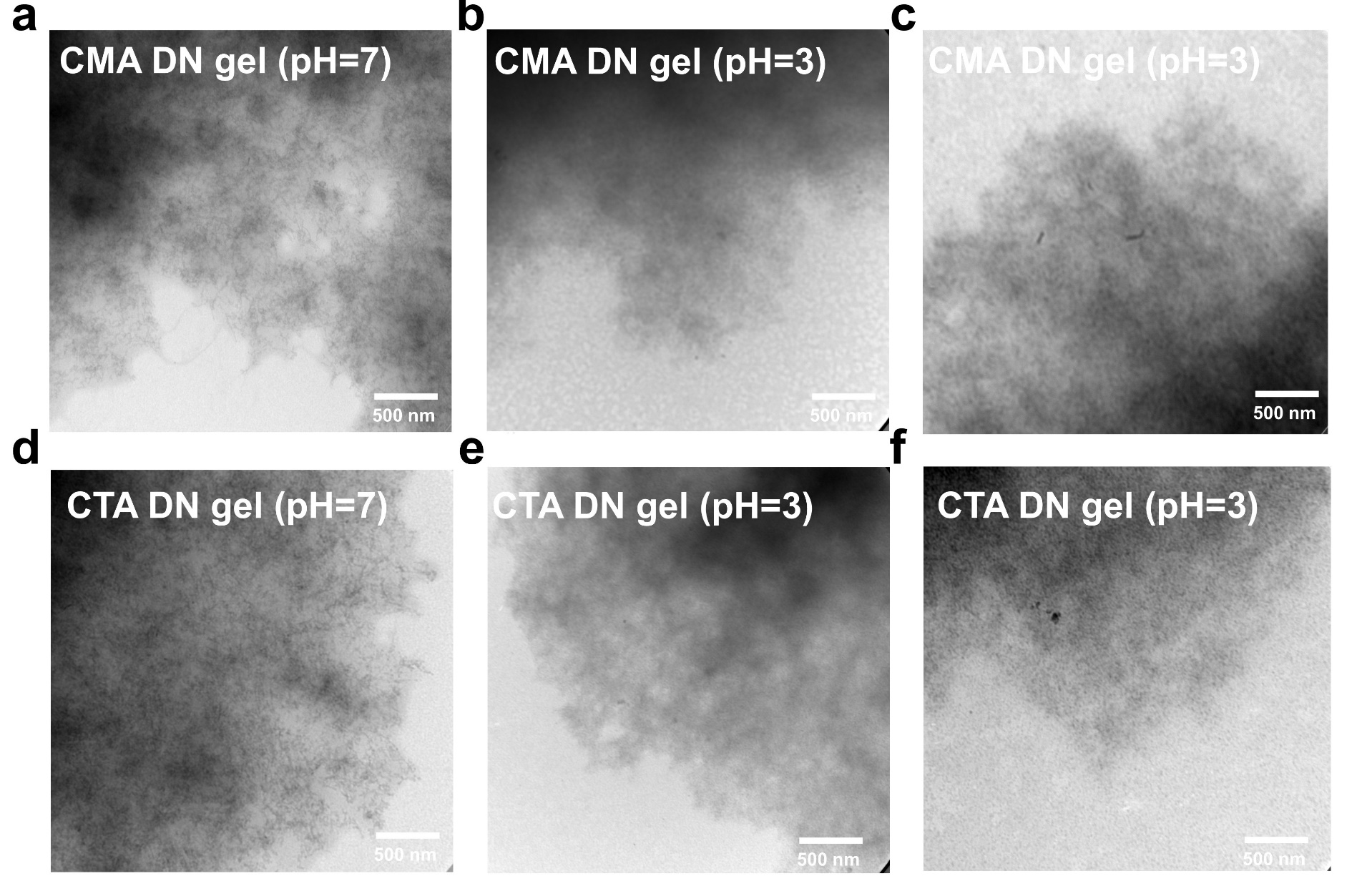


**Supplementary Figure 10:** TEM images of (a, b, c) 0.5% CMA DN gel and (d, e, f) 0.5% CTA DN gel after being immersed in (a, d) pH 7 and (b, c, e, f) pH 3 solutions for 7 days. All observations were independently repeated 3 times to ensure the robustness of the results.


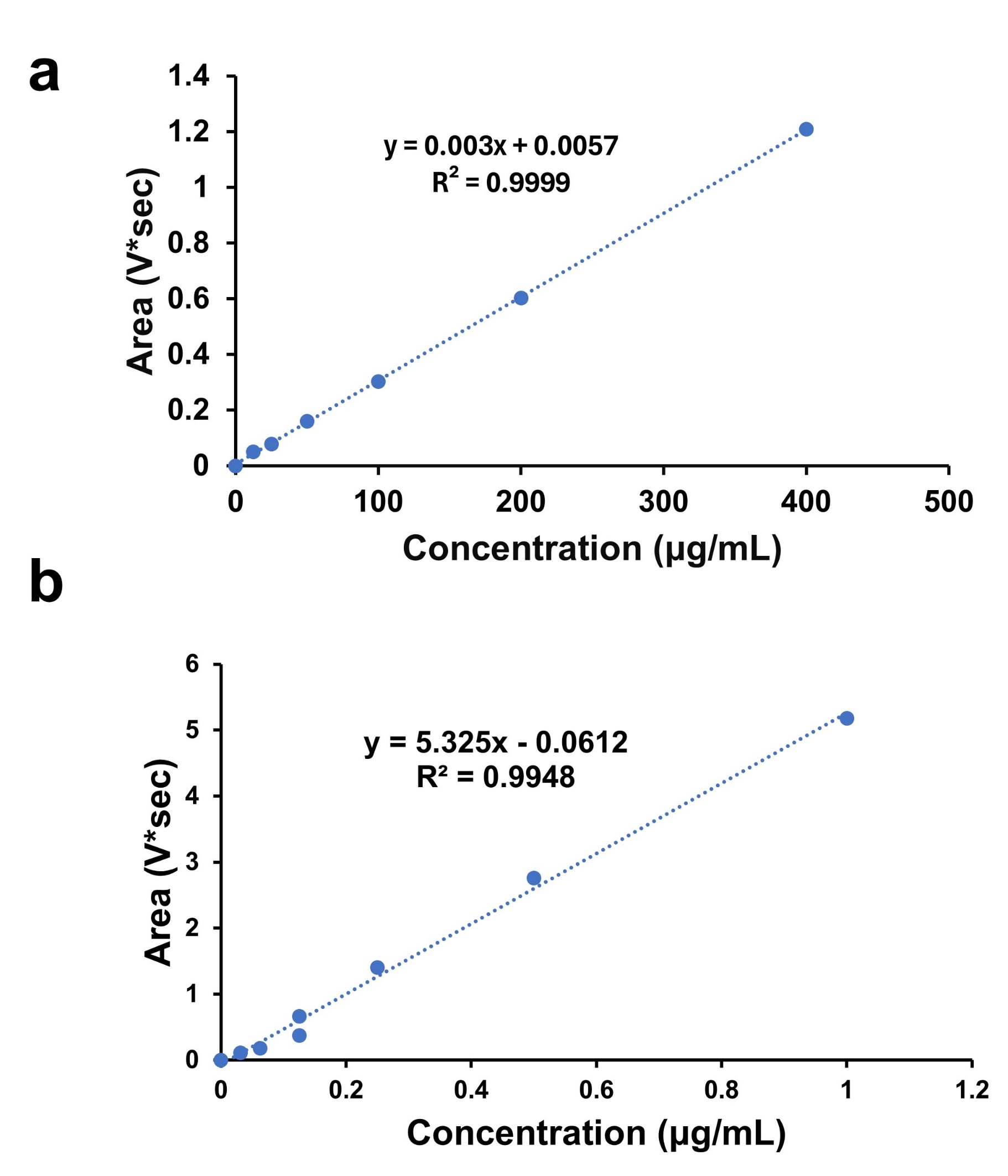


**Supplementary Figure 11:** Calibration curve of (a) zhonshengmycin (t_r_ = 7.1 min at λ = 220 nm) and (b) Phe (t_r_ = 5.6 min at λ = 220 nm) obtained by HPLC.


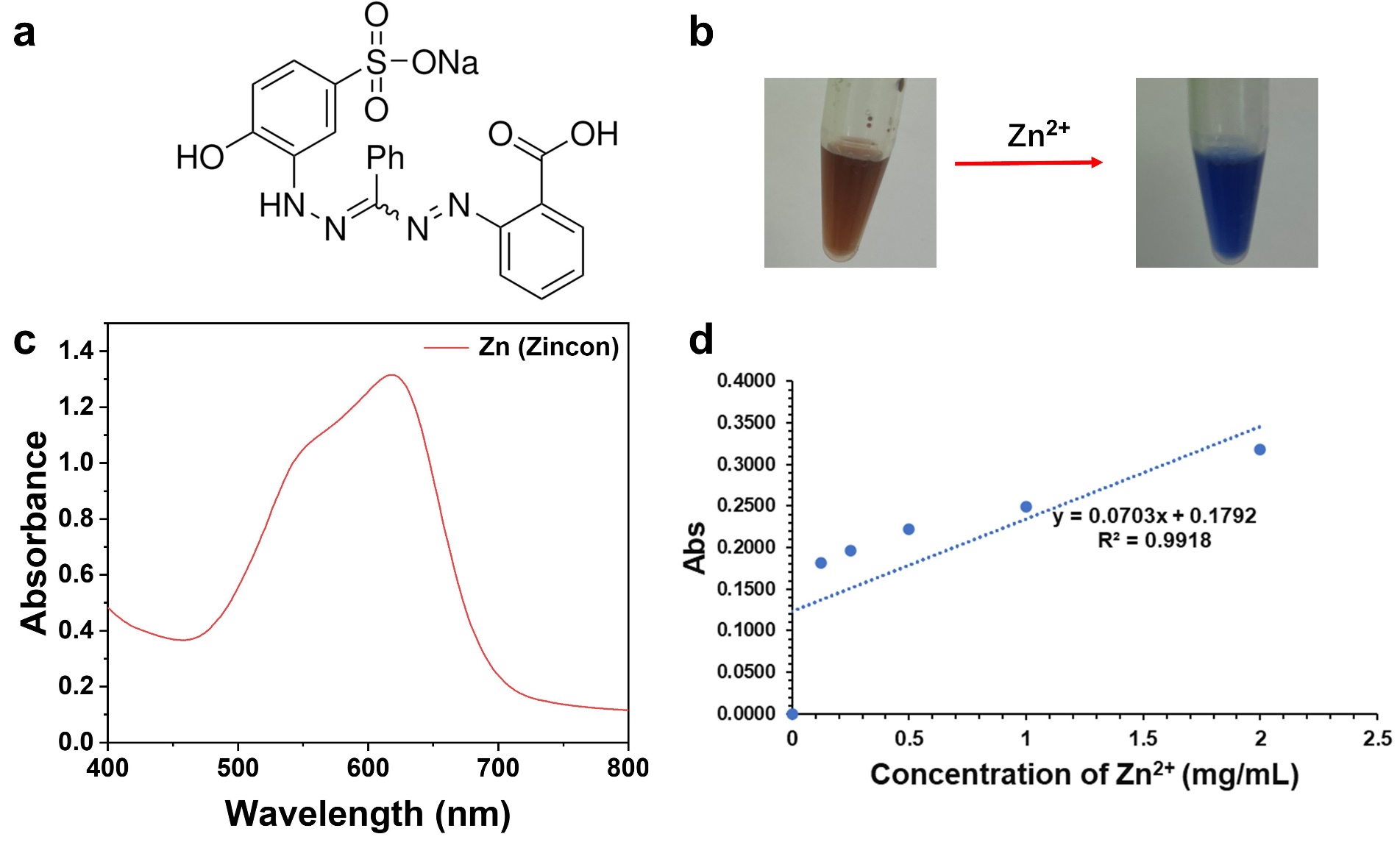


**Supplementary Figure 12:** (a) Chemical structure of Zincon monosodium salt. (b) Visual color change upon complex formation between Zincon monosodium salt and Zn^2+^ in aqueous solution. (c) UV-Vis absorption spectrum of the Zn^2+^-Zincon complex (Zn (Zincon)), confirming characteristic spectral features of the coordination interaction. (d) Calibration curve for Zn^2+^ quantification based on absorbance of the Zn (Zincon) complex, as determined by UV-Vis spectrophotometry.


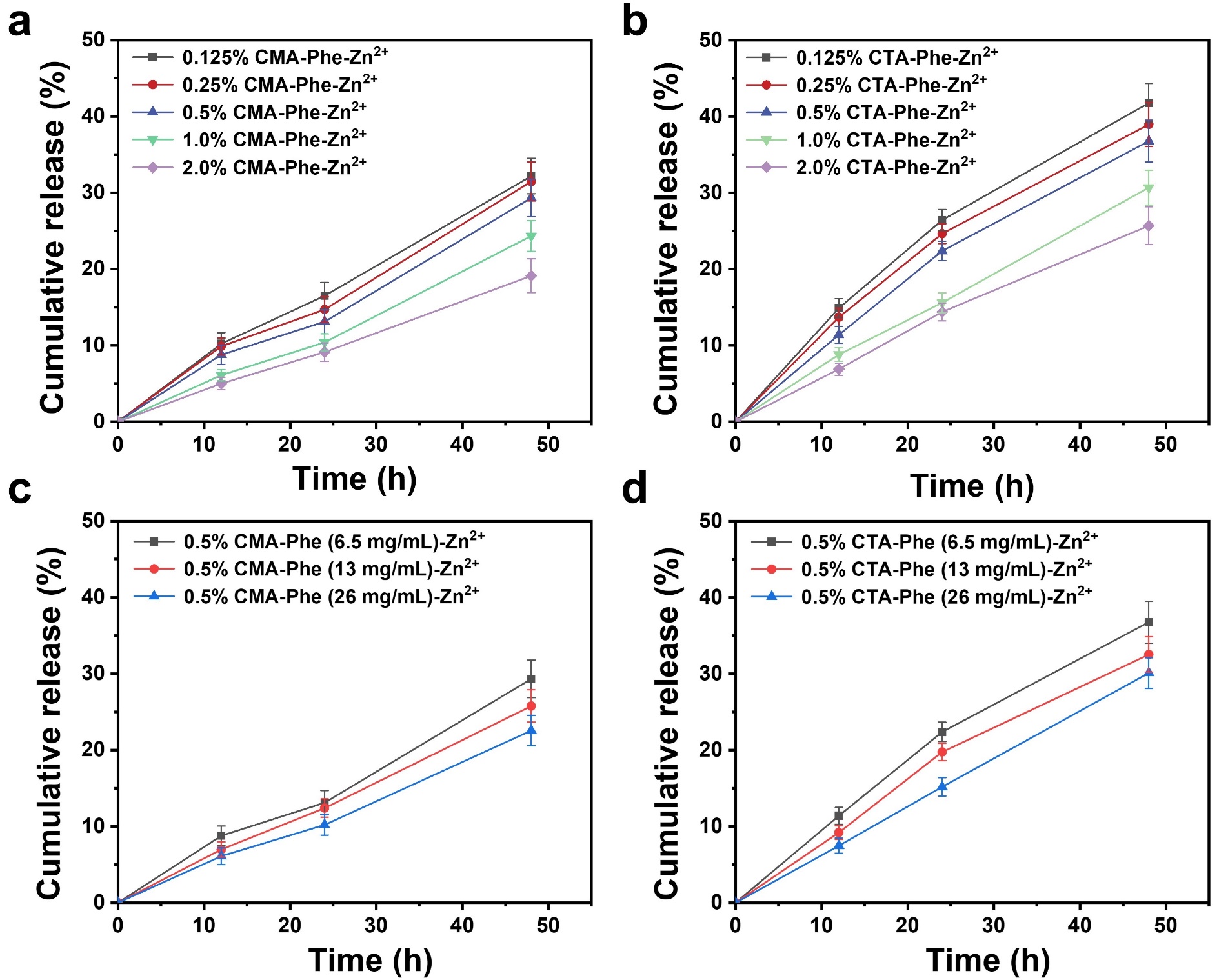


**Supplementary Figure 13:** Pesticide released from (a) the CMA or (b) CTA DN gels with different concentrations of CMA, CTA, and Phe at pH 7. The concentration of Phe was 6.5 mg/mL. All measurements were performed with 3 technical replicates for each group and the experiments were independently repeated 3 times. Data are presented as mean ± SD (n = 3 independent experiments).


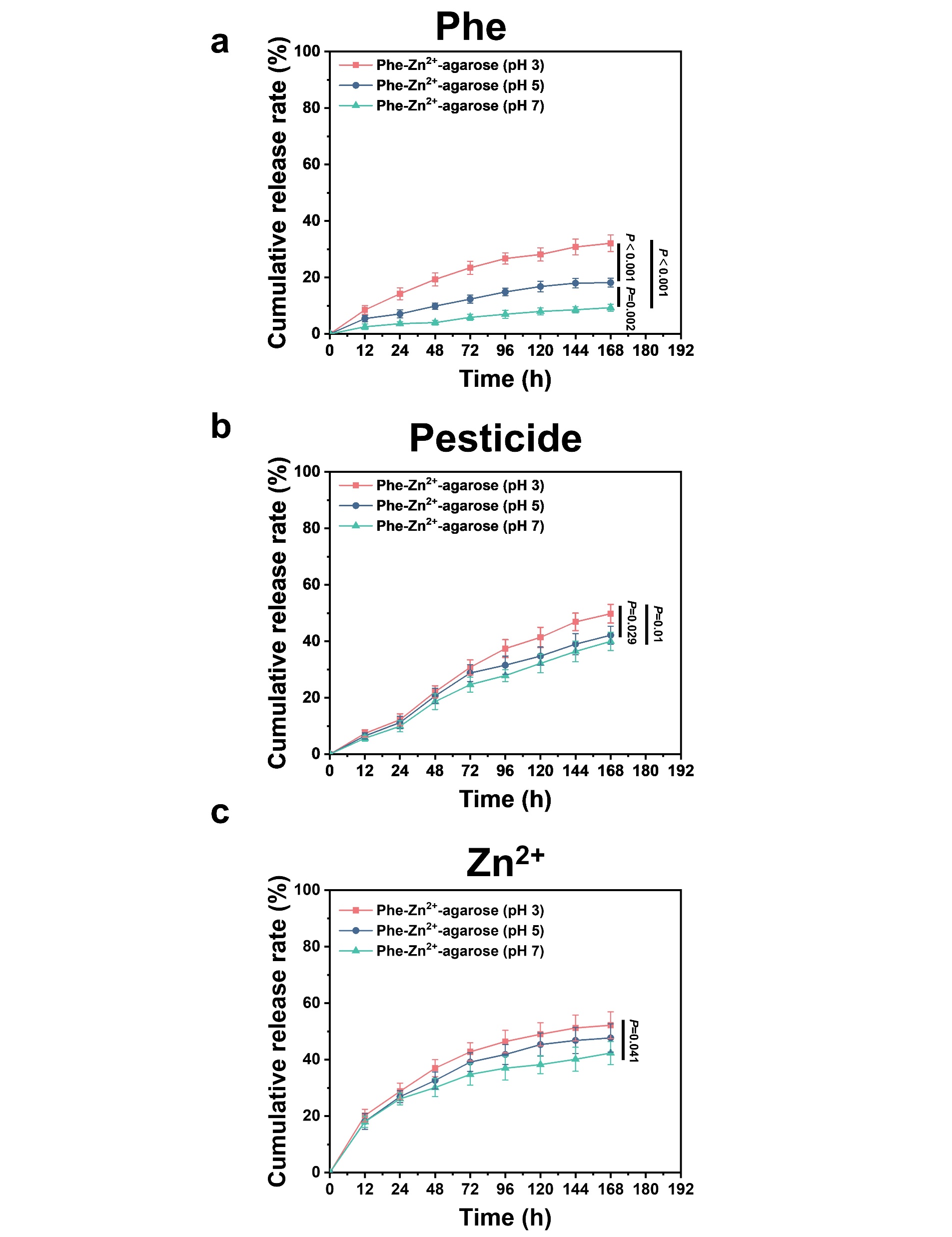


**Supplementary Figure 14:** Cumulative release profiles of (a) Phe, (b) pesticide, and (c) Zn^2+^ from Phe-Zn^2+^-agarose gels immersed in solutions of pH 3, 5, and 7. Statistical differences were determined using one-way ANOVA with post hoc test. All measurements were performed with 3 technical replicates for each group and the experiments were independently repeated 3 times. Data are presented as mean ± SD (n = 3 independent experiments).


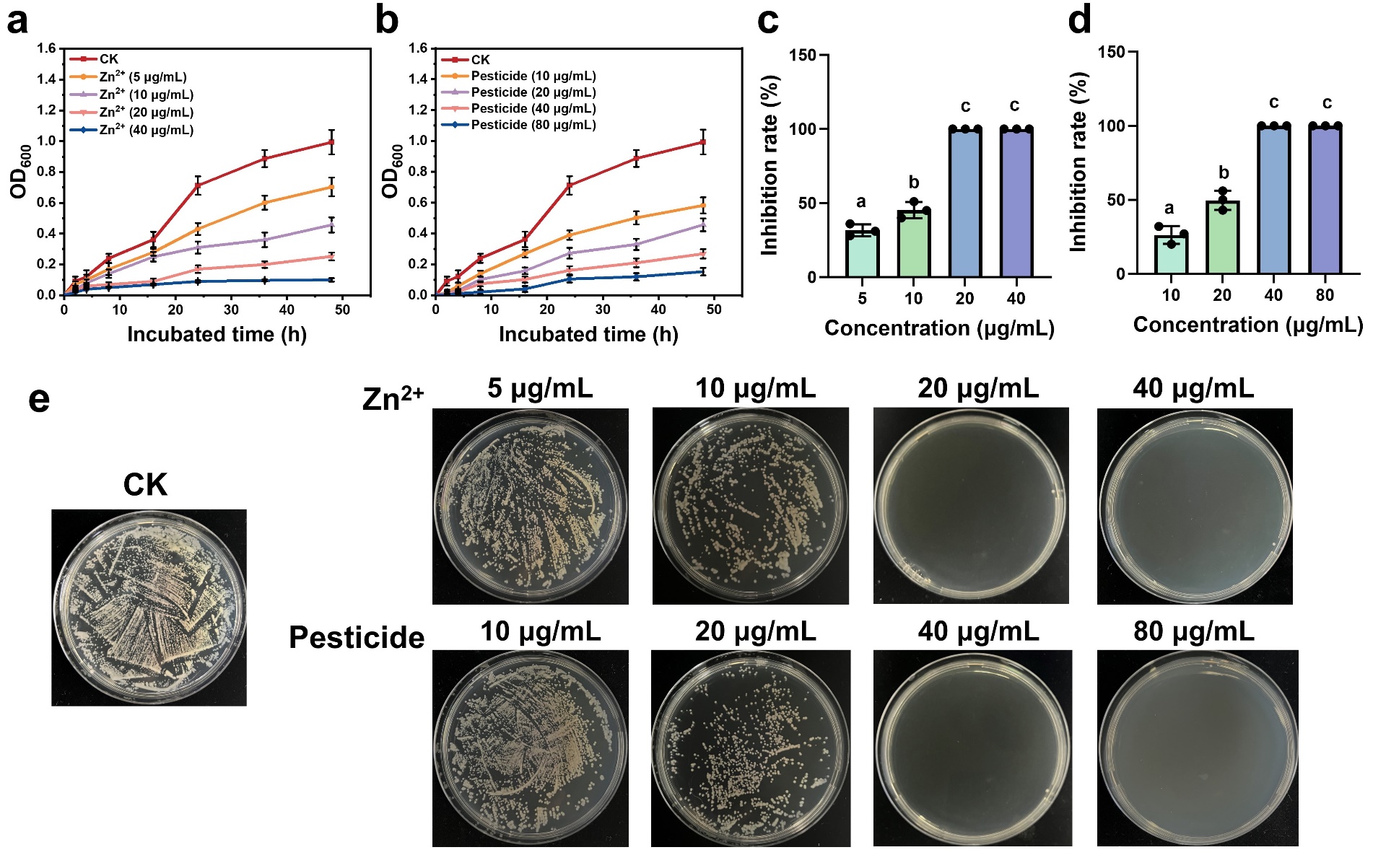


**Supplementary Figure 15:** Growth curves of *R. solanacearum* exposed to varying concentrations of (a) Zn^2+^ and (b) the pesticide, as monitored by the optical density at 600 nm (OD_600_) over time. Quantitative analysis of the inhibitory effects of (c) Zn^2+^ and (d) the pesticide on *R. solanacearum* growth at different concentrations. (e) Concentration-dependent inhibition of single-colony formation of *R. solanacearum* by Zn^2+^ and the pesticide. CK means the negative control (distilled water). The mean values presented in each bar, accompanied by different letters (a, b, c, *P* < 0.05), indicate significant differences based on one-way ANOVA with post hoc test (inhibition rate (panel c): *F_3, 8_*= 332.198, *p*< 0.001, inhibition rate (panel d): *F_3, 8_*= 209.299, *p*< 0.001). All measurements were performed with 6 technical replicates and 3 biological replicates for each group. Data are presented as mean ± SD (n = 3 independent experiments).


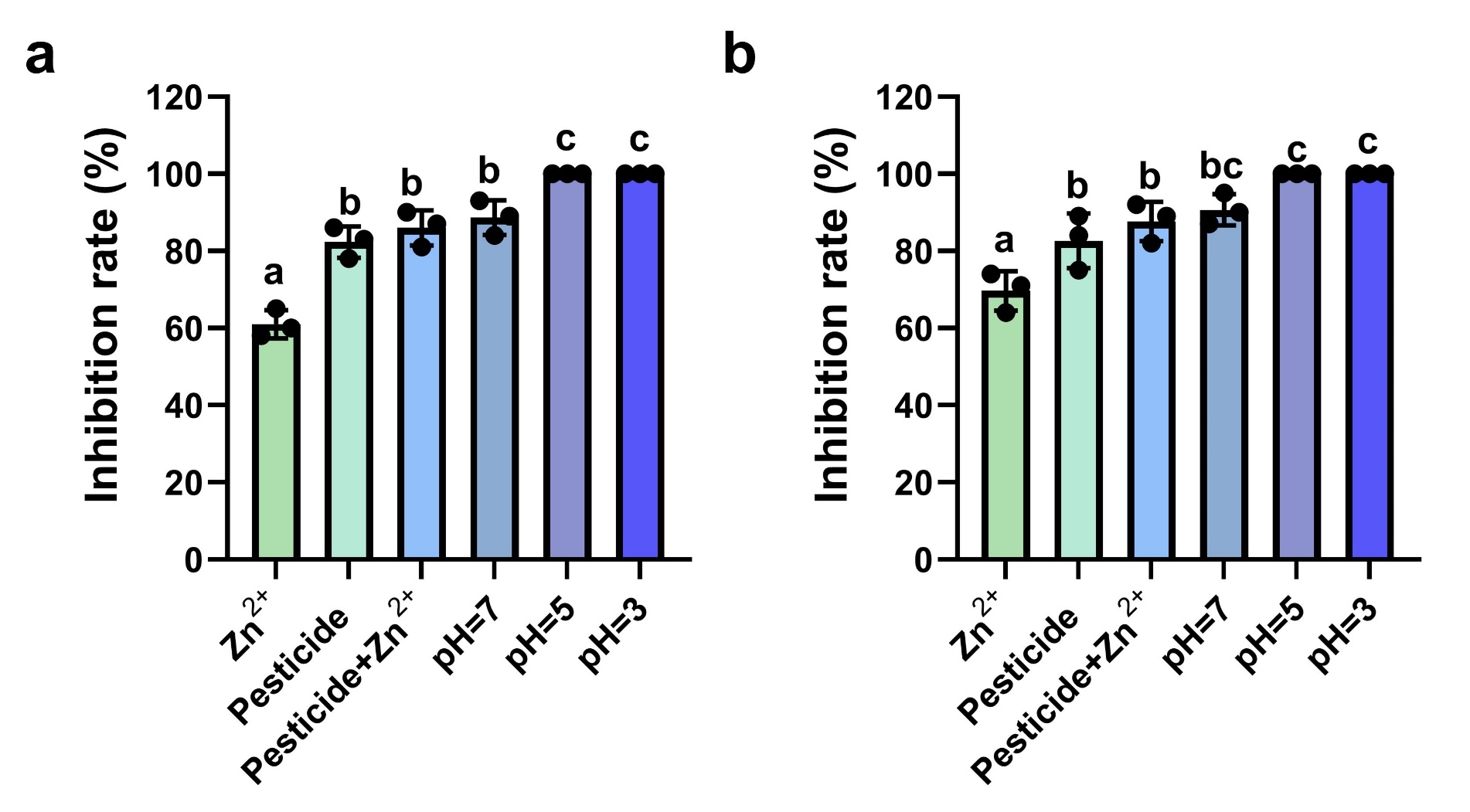


**Supplementary Figure 16:** Antibacterial efficacy of the released solutions derived from (a) the CMA and (b) CTA DN gels against *R. solanacearum* under various initial pH conditions. Prior to testing, all released solutions were adjusted to pH 7 to ensure comparability. The mean values presented in each bar, accompanied by different letters (a, b, c, *P* < 0.05), indicate significant differences between the groups based on one-way ANOVA with post hoc test (inhibition rate (panel a): *F_5, 12_*= 52.823, *p*< 0.001, inhibition rate (panel b): *F_5, 12_*= 19.868, *p*< 0.001). All measurements were performed with 6 technical replicates and 3 biological replicates for each group. Data are presented as mean ± SD (n = 3 independent experiments).


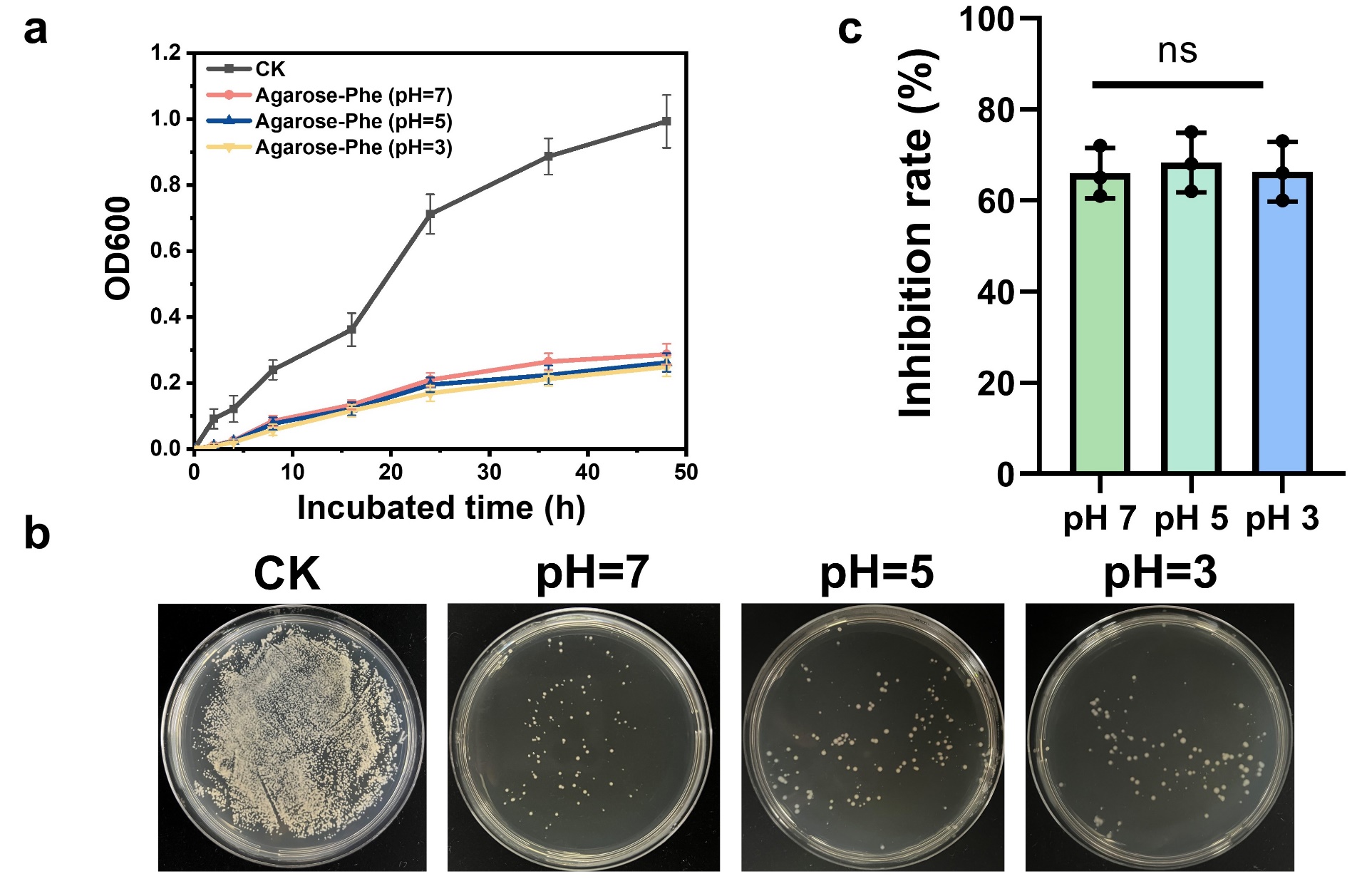


**Supplementary Figure 17:** Antibacterial activity of released solutions from agarose-Phe-Zn^2+^ gels prepared under varying pH conditions (pH 3, 5, and 7) against *R. solanacearum*. (a) Bacterial growth curves monitored over a 48-h period following the treatment with each released solution. (b) Inhibitory effects on the formation of single bacterial colonies. (c) Quantitative analysis of inhibition rates. Prior to testing, all released solutions were adjusted to pH 7 to ensure comparability. CK means the negative control (distilled water). ns indicates no significant difference (*P* ≥ 0.05), as determined by one-way ANOVA with post hoc test. The vertical bars indicate standard deviations. All measurements were performed with 6 technical replicates and 3 biological replicates for each group. Data are presented as mean ± SD (n = 3 independent experiments).


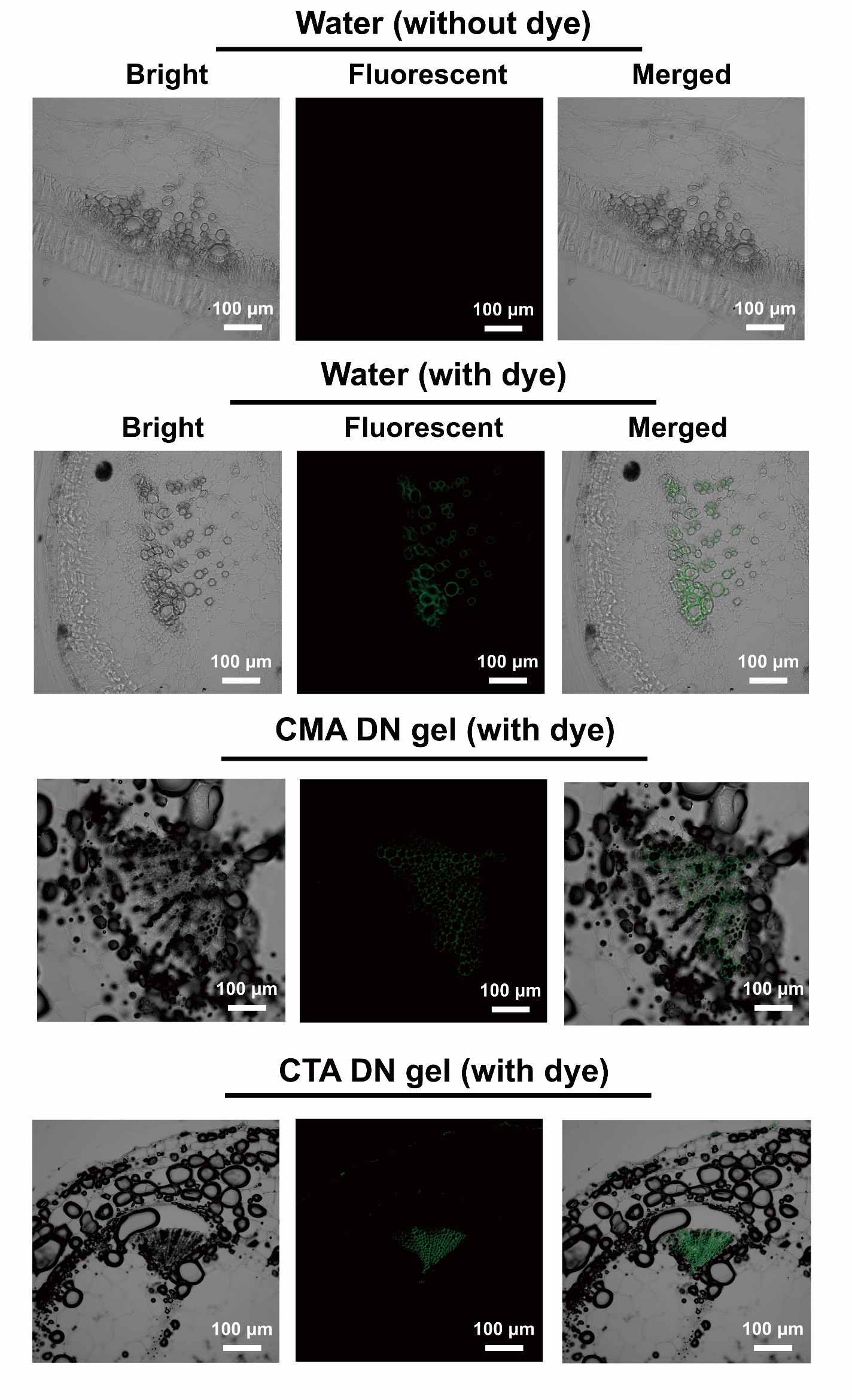


**Supplementary Figure 18:** Confocal laser scanning microscopy images of tomato stems following treatment with the CMA and CTA DN gels loaded with 5(6)‑carboxyfluorescein. All observations were independently repeated 3 times to ensure the robustness of the results.


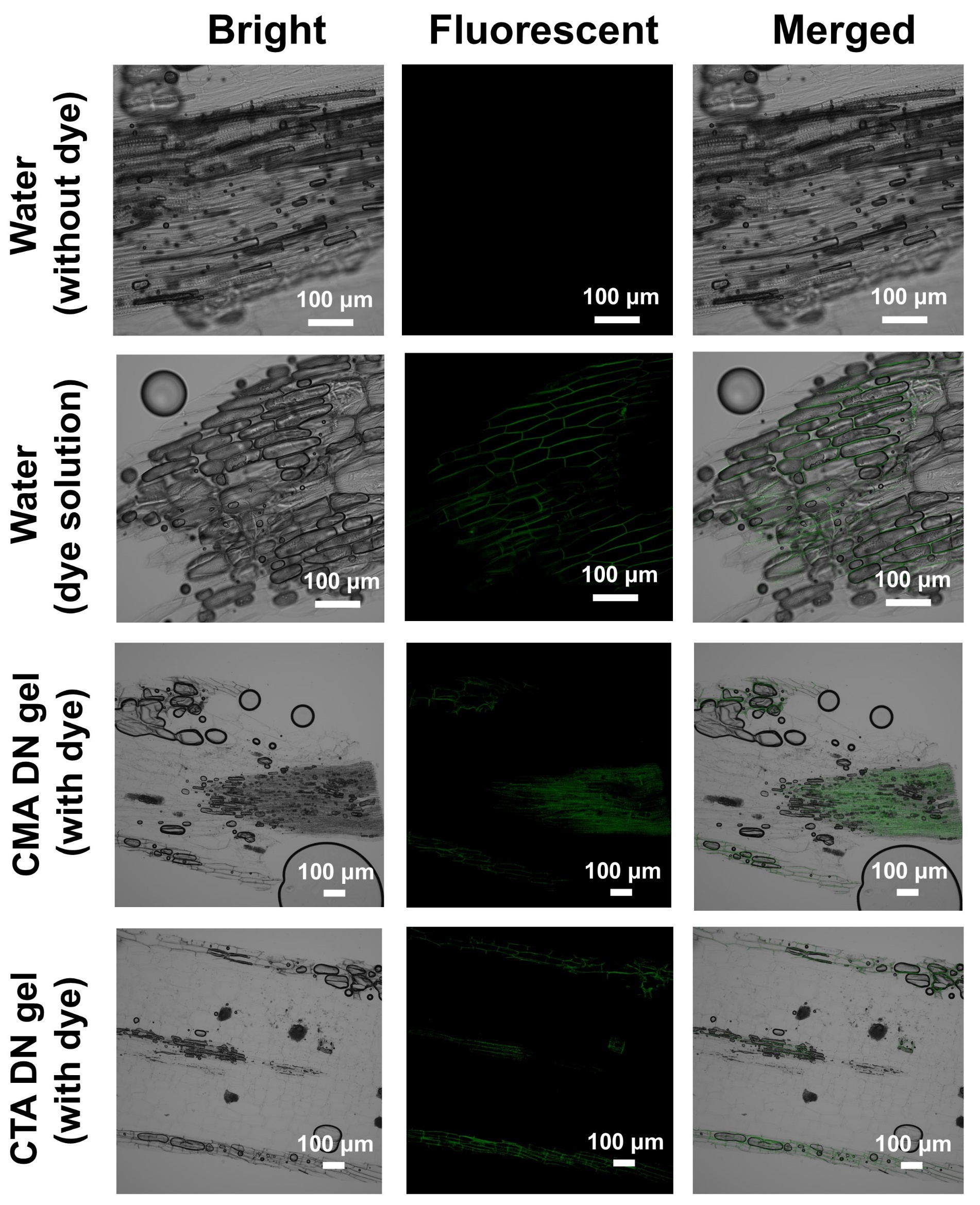


**Supplementary Figure 19:** Confocal laser scanning microscopy images illustrating the transport and distribution of 5(6)-carboxyfluorescein within tomato root tissues following the treatment with the CMA/CTA DN gels. Green fluorescence indicates the presence and localization of 5(6)-carboxyfluorescein, reflecting hydrogel-mediated delivery dynamics in the plant. All observations were independently repeated 3 times to ensure the robustness of the results.


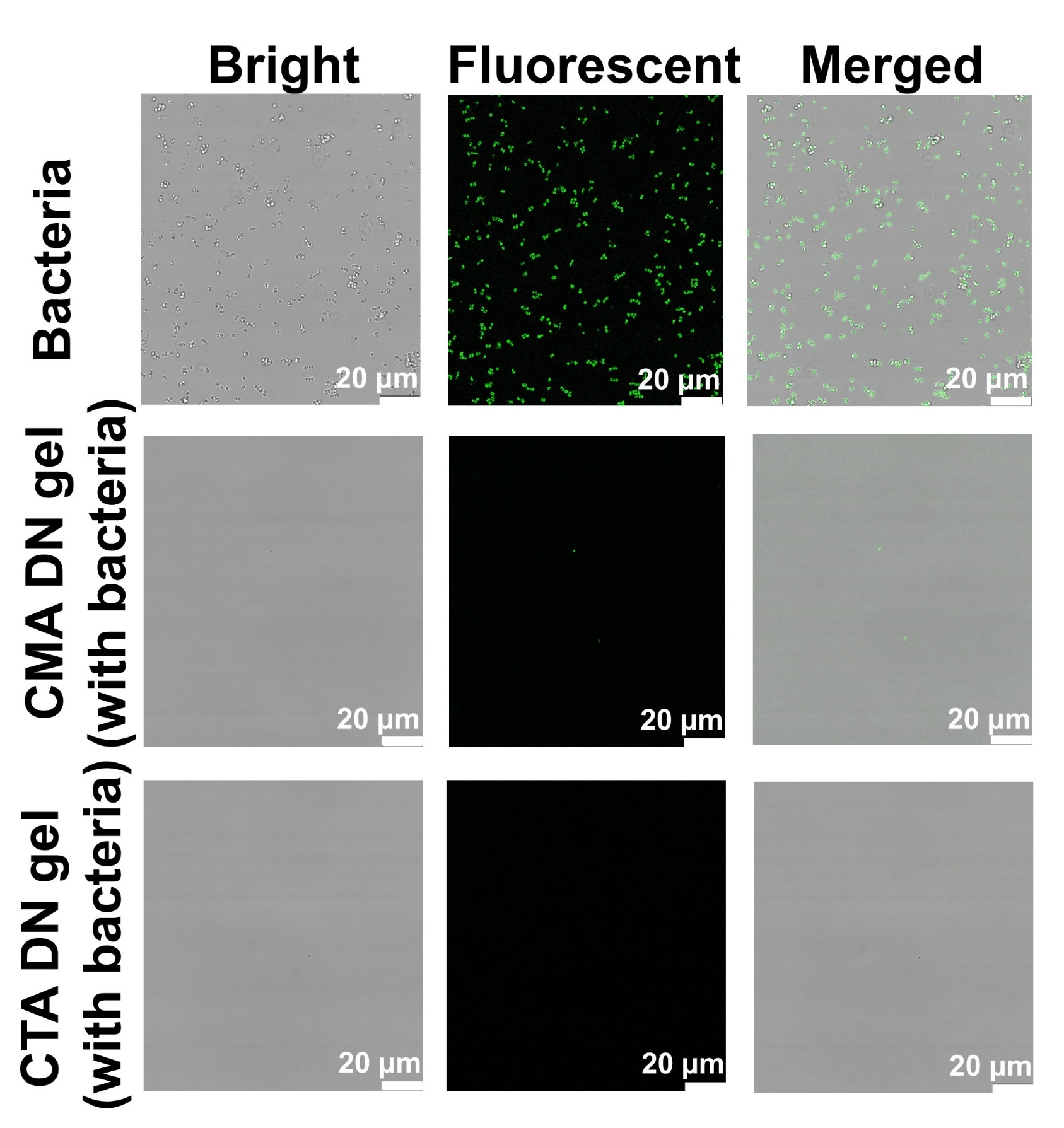


**Supplementary Figure 20:** Confocal fluorescence images of *R. solanacearum*-GFP after exposure to the CMA/CTA DN gels. All observations were independently repeated 3 times to ensure the robustness of the results.


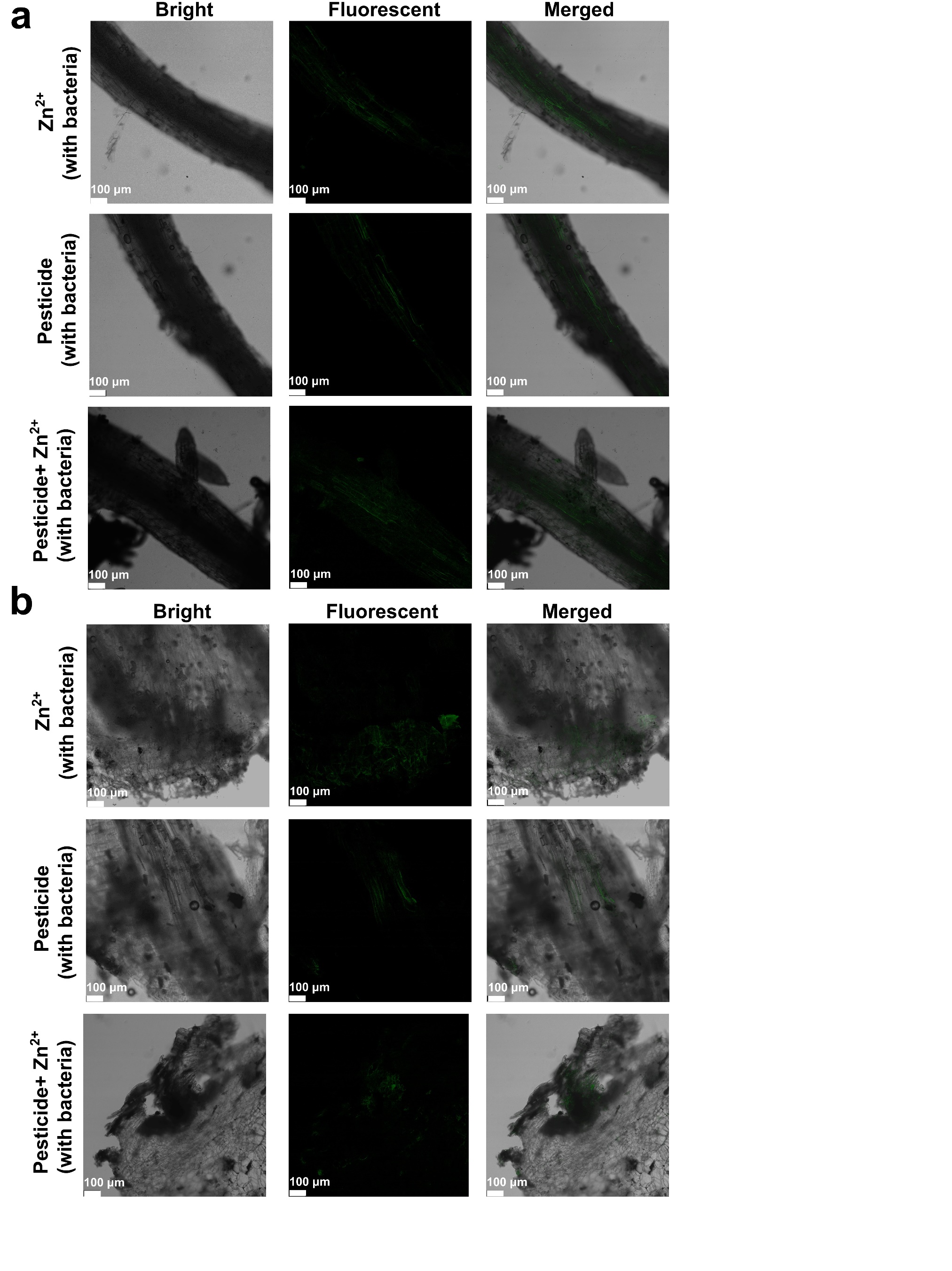


**Supplementary Figure 21:** Confocal laser scanning microscopy images showing the infection of (a) tomato root and (b) stem by *R. solanacearum*-GFP following the treatment with Zn^2+^, pesticide, and pesticide+Zn^2+^. Green fluorescence represents the localization of *R. solanacearum*-GFP, providing a visual indication of bacterial colonization in plant tissues. All observations were independently repeated 3 times to ensure the robustness of the results.


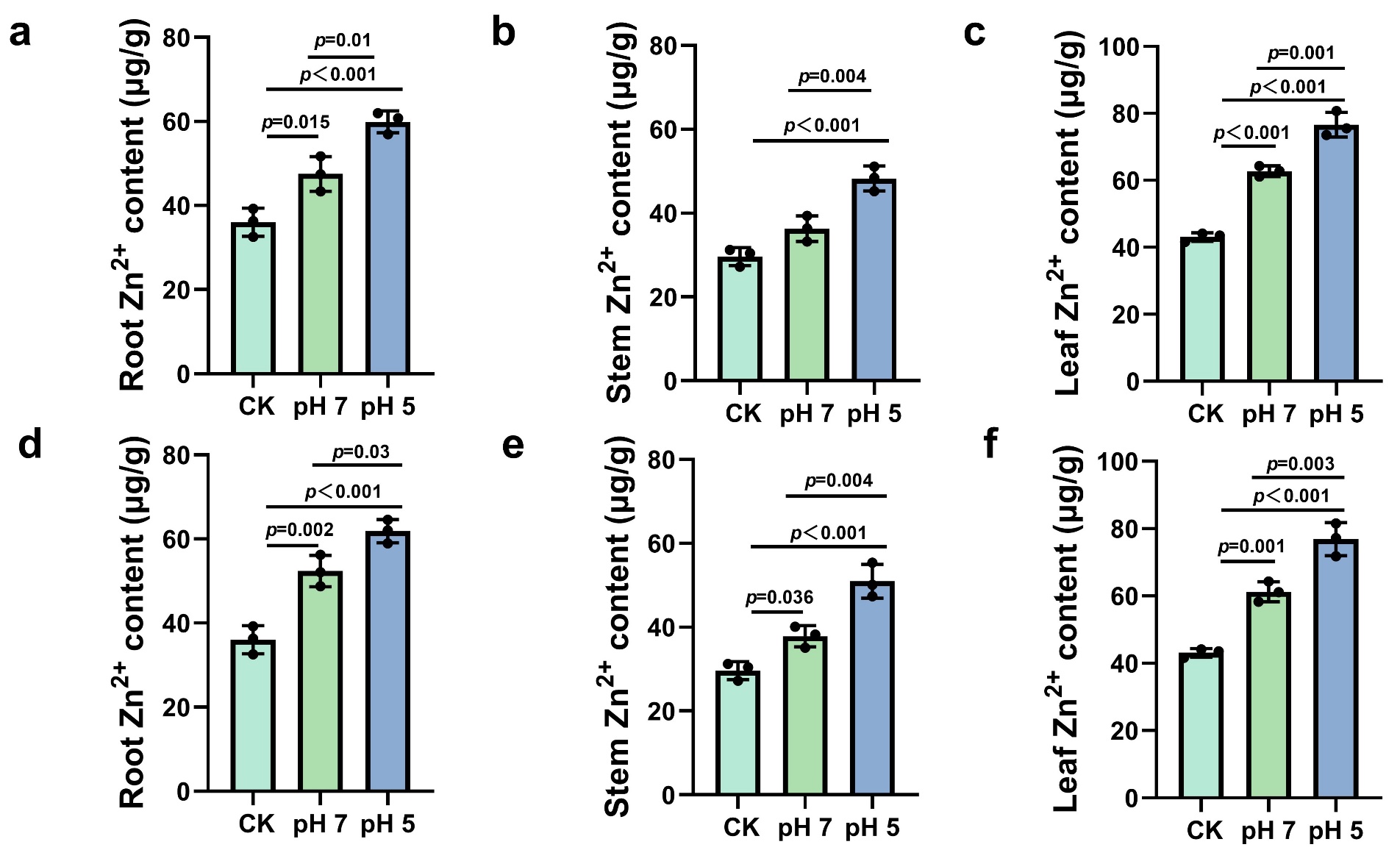


**Supplementary Figure 22:** Zn^2+^ accumulation (assessed by ICP-MS) in the roots, stems, and leaves of tomato plants treated with the released solutions from (a-c) the CMA DN gel and (d-f) CTA DN gel under pH conditions of 5 and 7. Prior to testing, all released solutions were adjusted to pH 7 to ensure comparability. Statistical differences were determined using one-way ANOVA with post hoc test. All measurements were performed with 3 technical replicates and 3 biological replicates for each group. Data are presented as mean ± SD (n = 3 independent experiments).


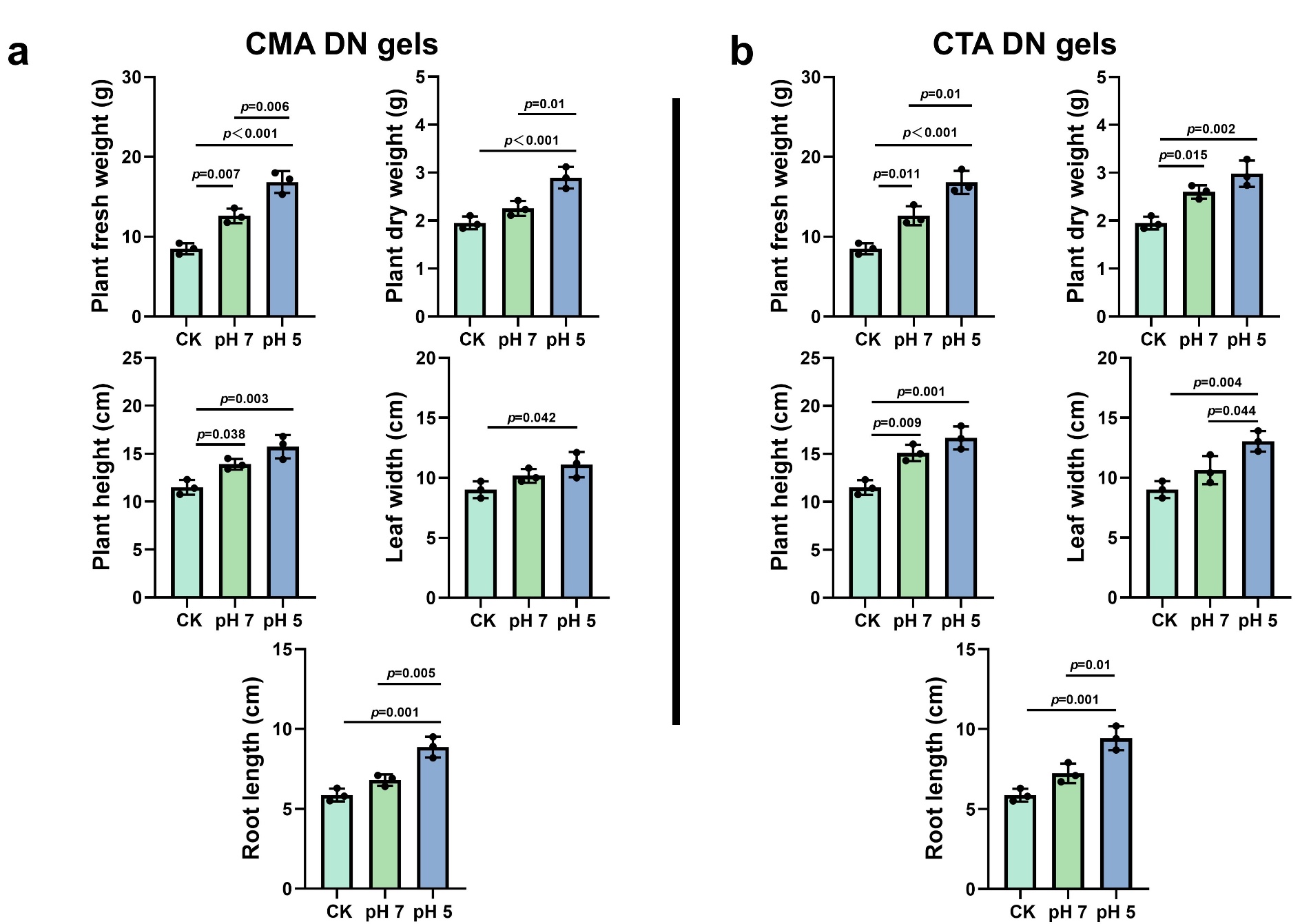


**Supplementary Figure 23:** Evaluation of growth parameters, including fresh weight, dry weight, plant height, leaf width, and root length, in tomato plants treated with the released solutions from (a) the CMA DN gel and (b) CTA DN gel under pH conditions of 5 and 7. Prior to testing, all released solutions were adjusted to pH 7 to ensure comparability. Statistical differences were determined using one-way ANOVA with post hoc test. All measurements were performed with 3 technical replicates and 3 biological replicates for each group. Data are presented as mean ± SD (n = 3 independent experiments).


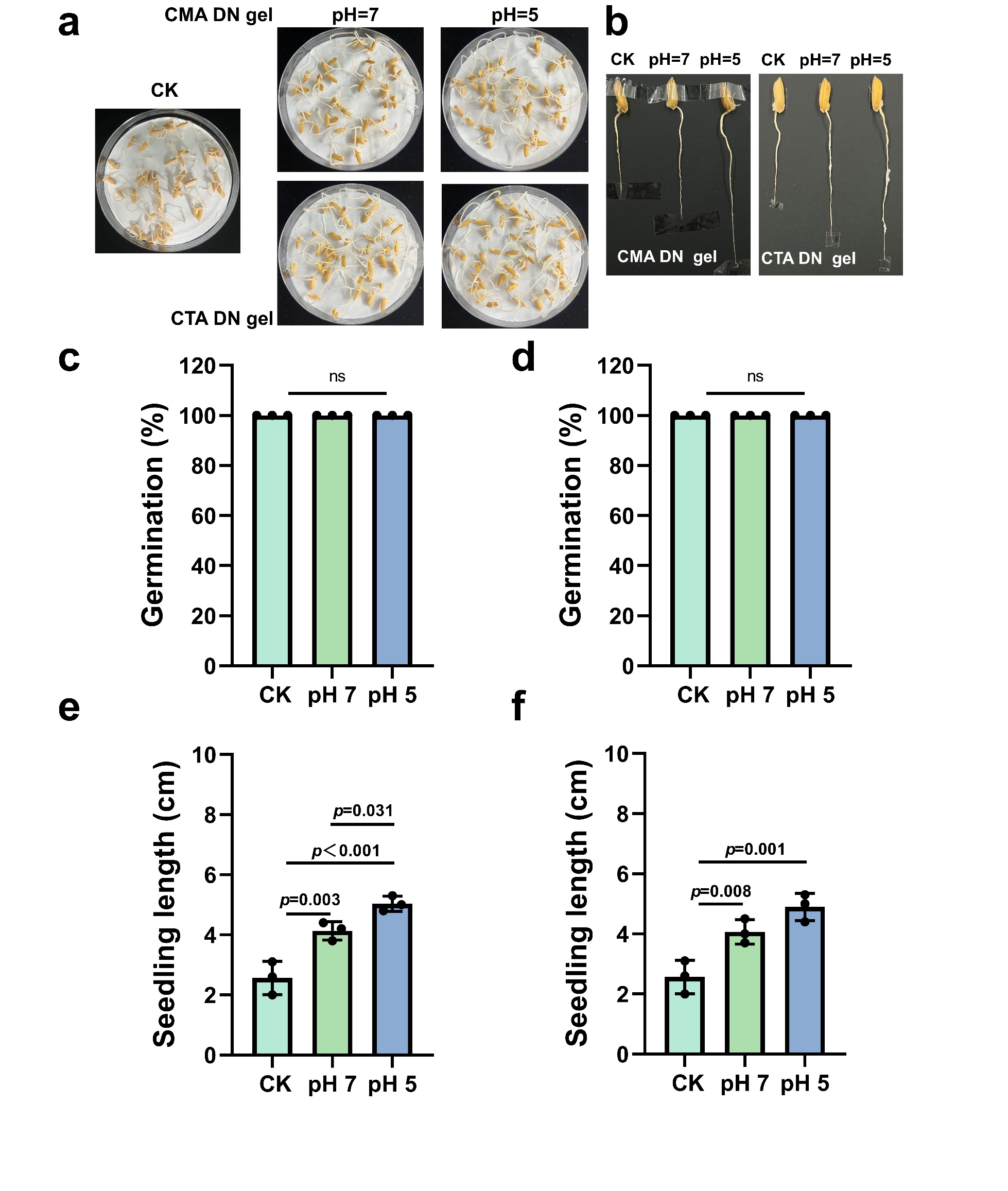


**Supplementary Figure 24:** (a) Evaluation of rice seed germination following a 7-day treatment with the solutions released from the CMA and CTA DN gels at pH 5 and pH 7. (b) Assessment of shoot length in rice seedlings after 7 days of exposure to the same released solutions. (c, d) Germination rate and (e, f) seedling length of rice seeds treated with the solutions released from (c, e) the CMA DN gel and (d, f) the CTA DN gel under pH conditions of 5 and 7. Prior to testing, all released solutions were adjusted to pH 7. Statistical differences were determined using one-way ANOVA with post hoc test. All measurements were performed with 40 technical replicates and 3 biological replicates for each group. ns indicates no statistical difference (*P* ≥ 0.05). Data are presented as mean ± SD (n = 3 independent experiments).


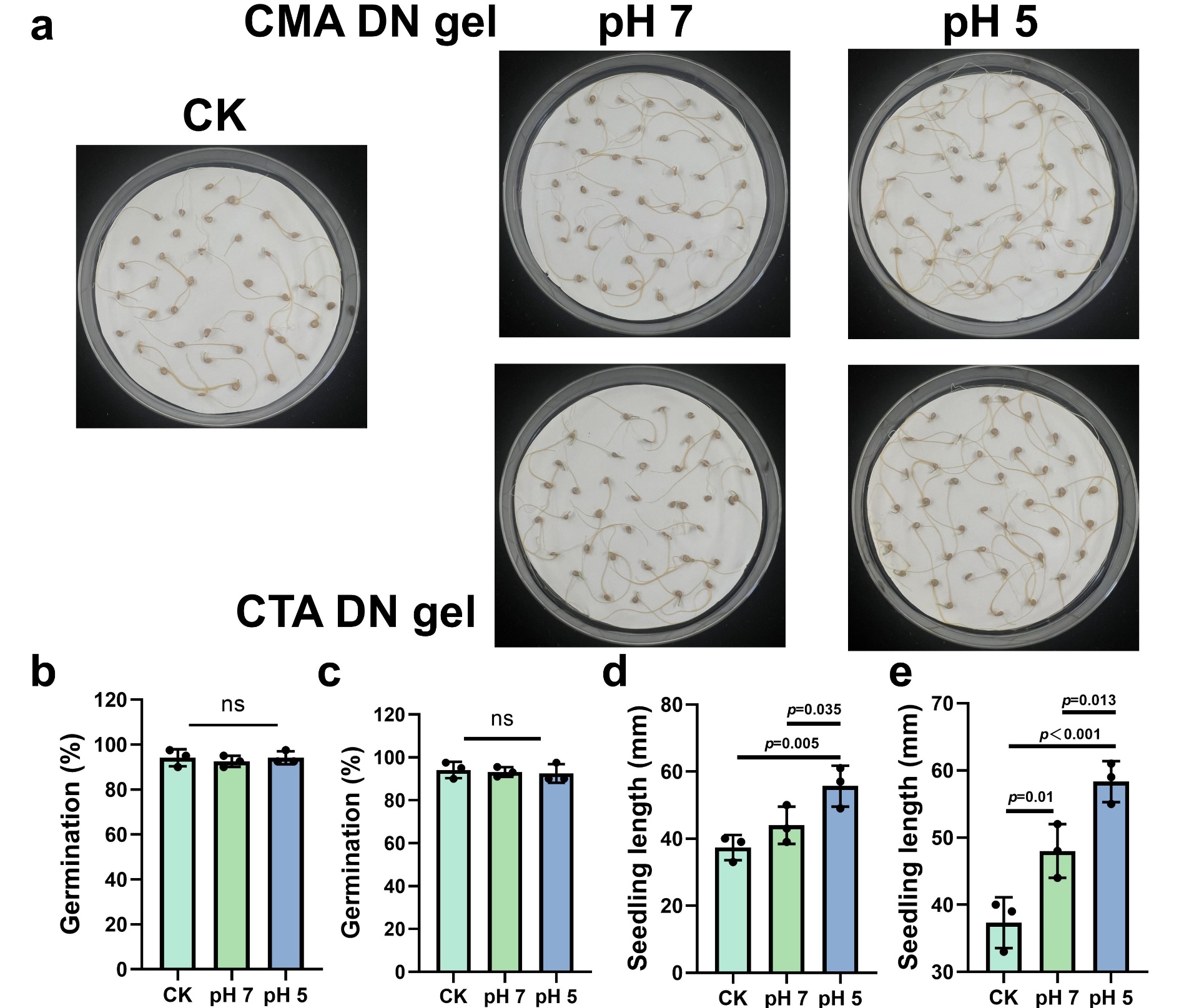


**Supplementary Figure 25:** (a) Germination images, (b, c) germination rate, and (d, e) seedling length of tomato seeds treated with the solutions released from (b, d) the CMA DN gel and (c, e) CTA DN gel under pH conditions of 5 and 7. Prior to testing, all released solutions were adjusted to pH 7. Statistical differences were determined using one-way ANOVA with post hoc test. All measurements were performed with 40 technical replicates and 3 biological replicates for each group. ns indicates no statistical difference (*P* ≥ 0.05). Data are presented as mean ± SD (n = 3 independent experiments).


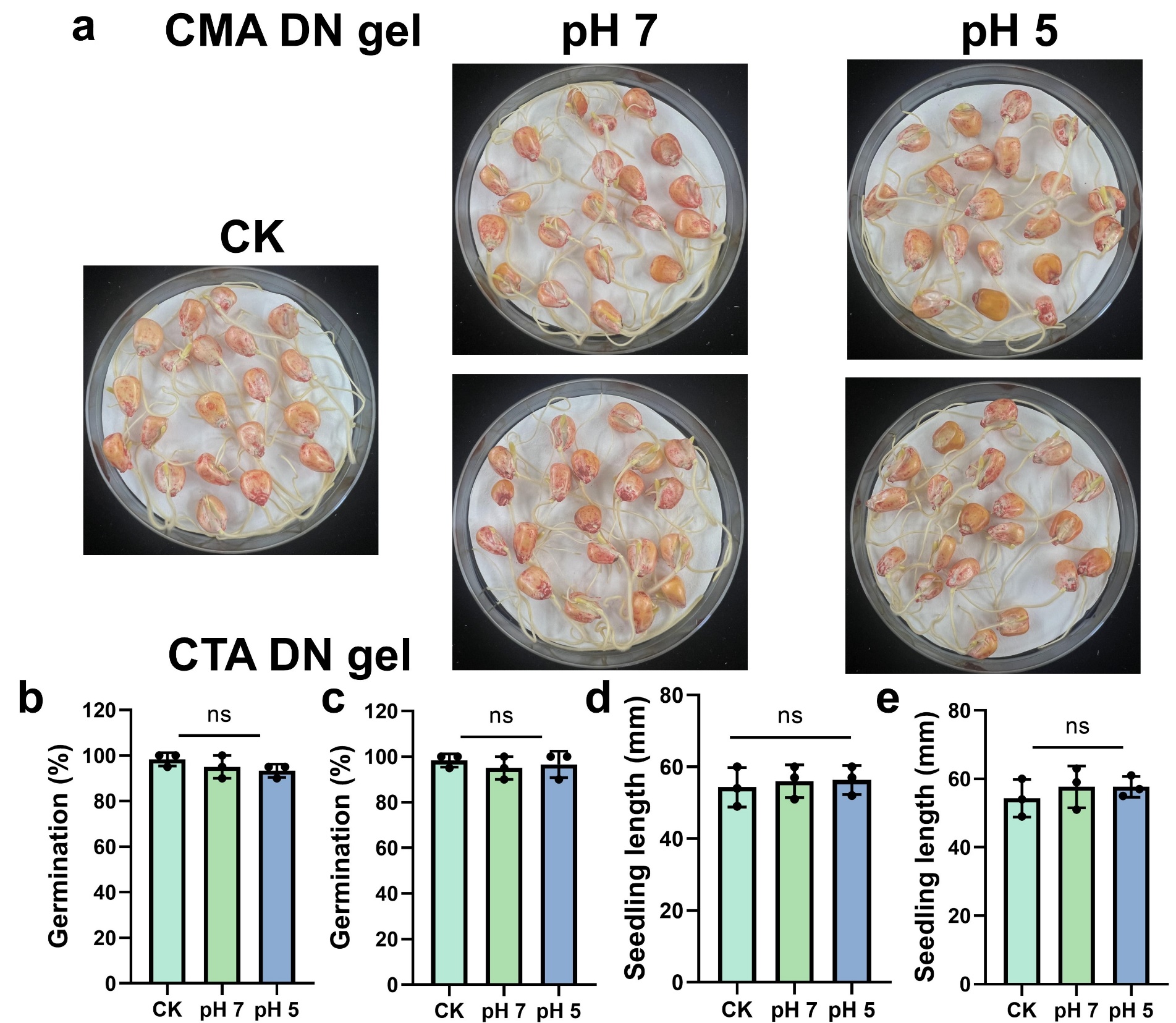


**Supplementary Figure 26:** (a) Germination images, (b, c) germination rate, and (d, e) seedling length of maize seeds treated with the solutions released from (b, d) the CMA DN gel and (c, e) CTA DN gel under pH conditions of 5 and 7. Prior to testing, all released solutions were adjusted to pH 7. ns indicates no significant difference (*P* ≥ 0.05), as determined by one-way ANOVA with post hoc test. All measurements were performed with 20 technical replicates and 3 biological replicates for each group. Data are presented as mean ± SD (n = 3 independent experiments).


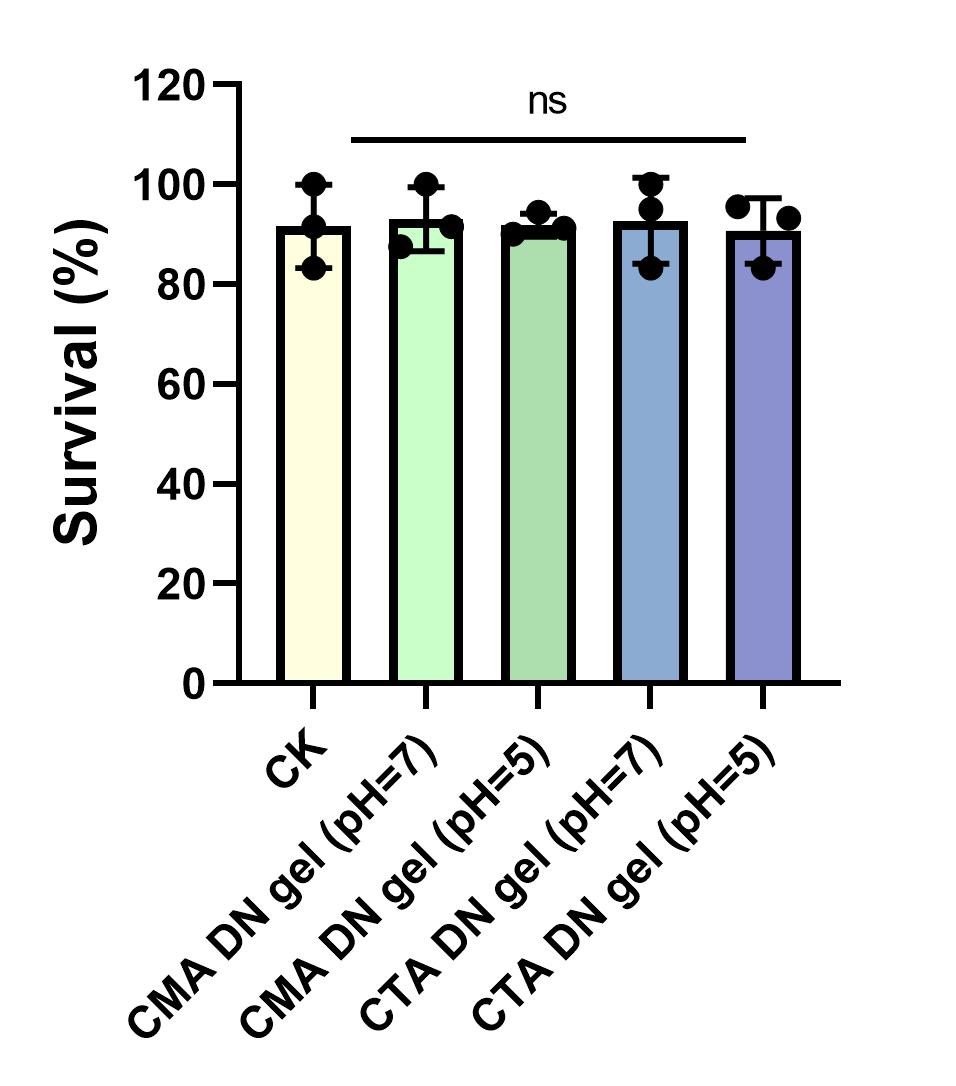


**Supplementary Figure 27:** Earthworm survival rate following exposure to the CMA and CTA DN gels at pH 5 and 7. The CMA/CTA DN gels were mixed with soils adjusted to different pH levels (pH 5 and 7), and earthworms were co-cultured in these treated soils. ns indicates no significant difference (*P* ≥ 0.05), as determined by one-way ANOVA with post hoc test. All measurements were performed with 20 technical replicates and 3 biological replicates for each group. Data are presented as mean ± SD (n = 3 independent experiments).

**Field Experiment 1 (2024):**


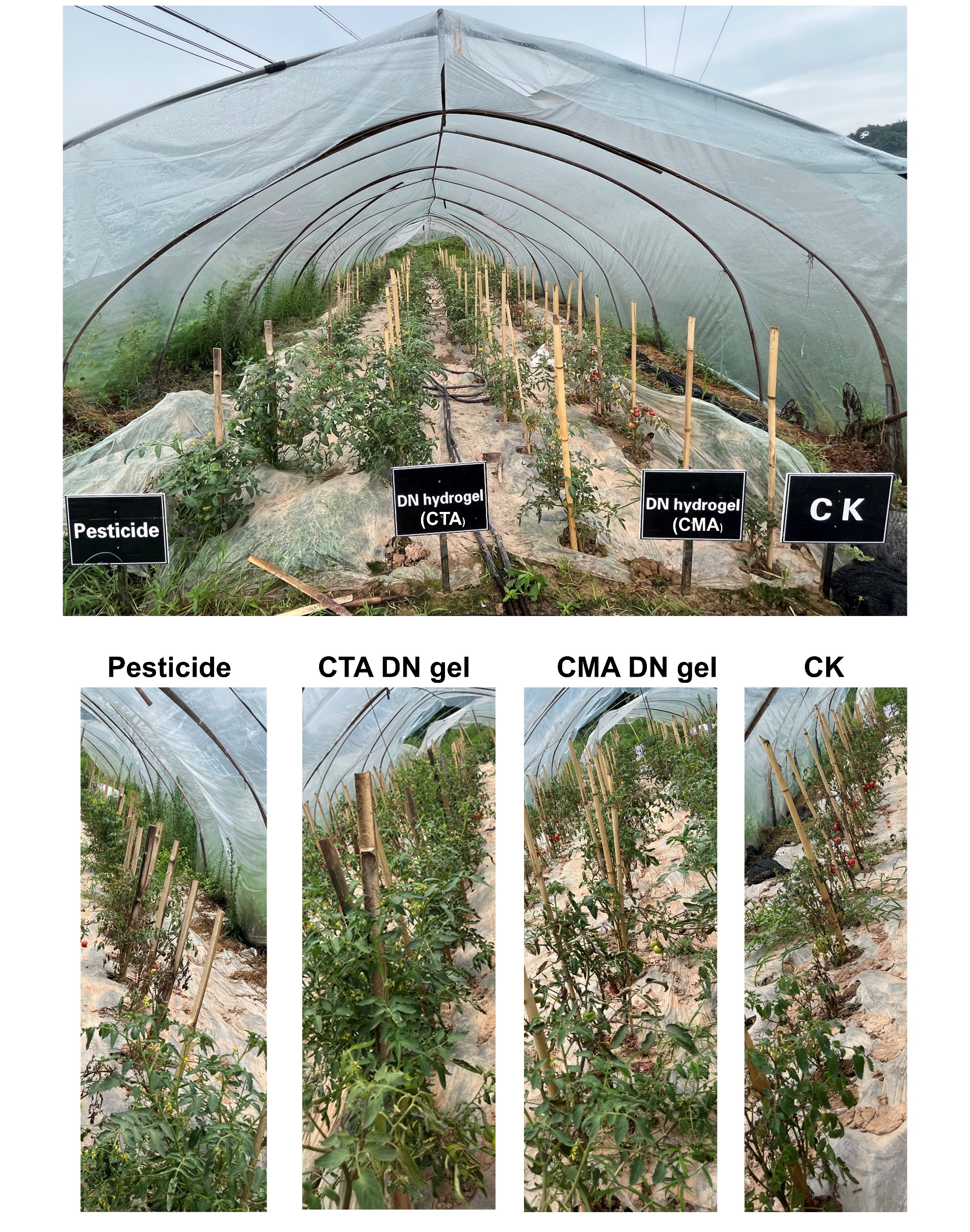


**Supplementary Figure 28:** Representative field photographs of tomato plants taken two weeks post-treatment with the pesticide, CMA DN gel, and CTA DN gel, illustrating the visual differences in plant health and disease progression among the treated groups.


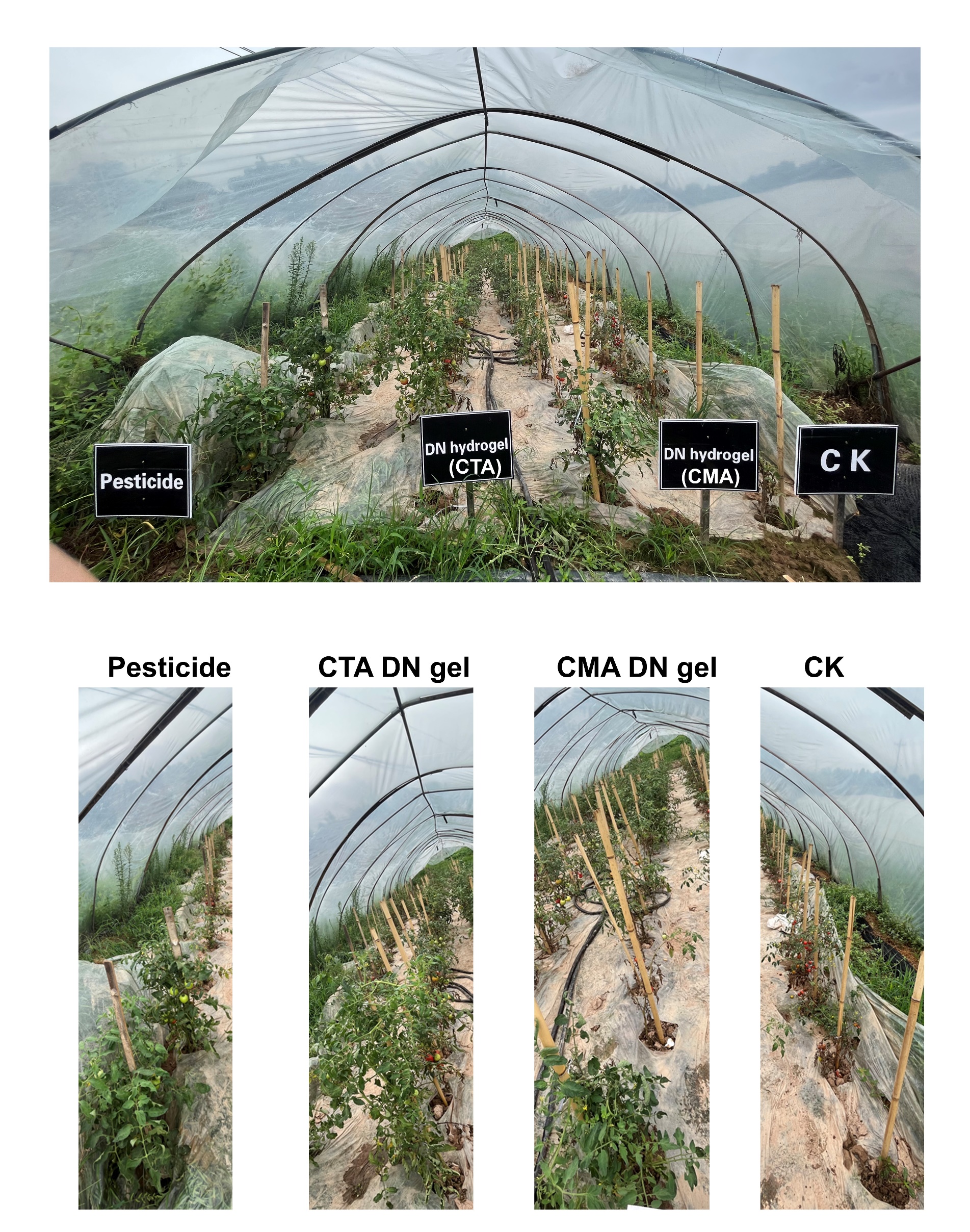


**Supplementary Figure 29:** Representative field photographs of tomato plants taken three weeks post-treatment with the pesticide, CMA DN gel, and CTA DN gel, illustrating the visual differences in plant health and disease progression among treatment groups.


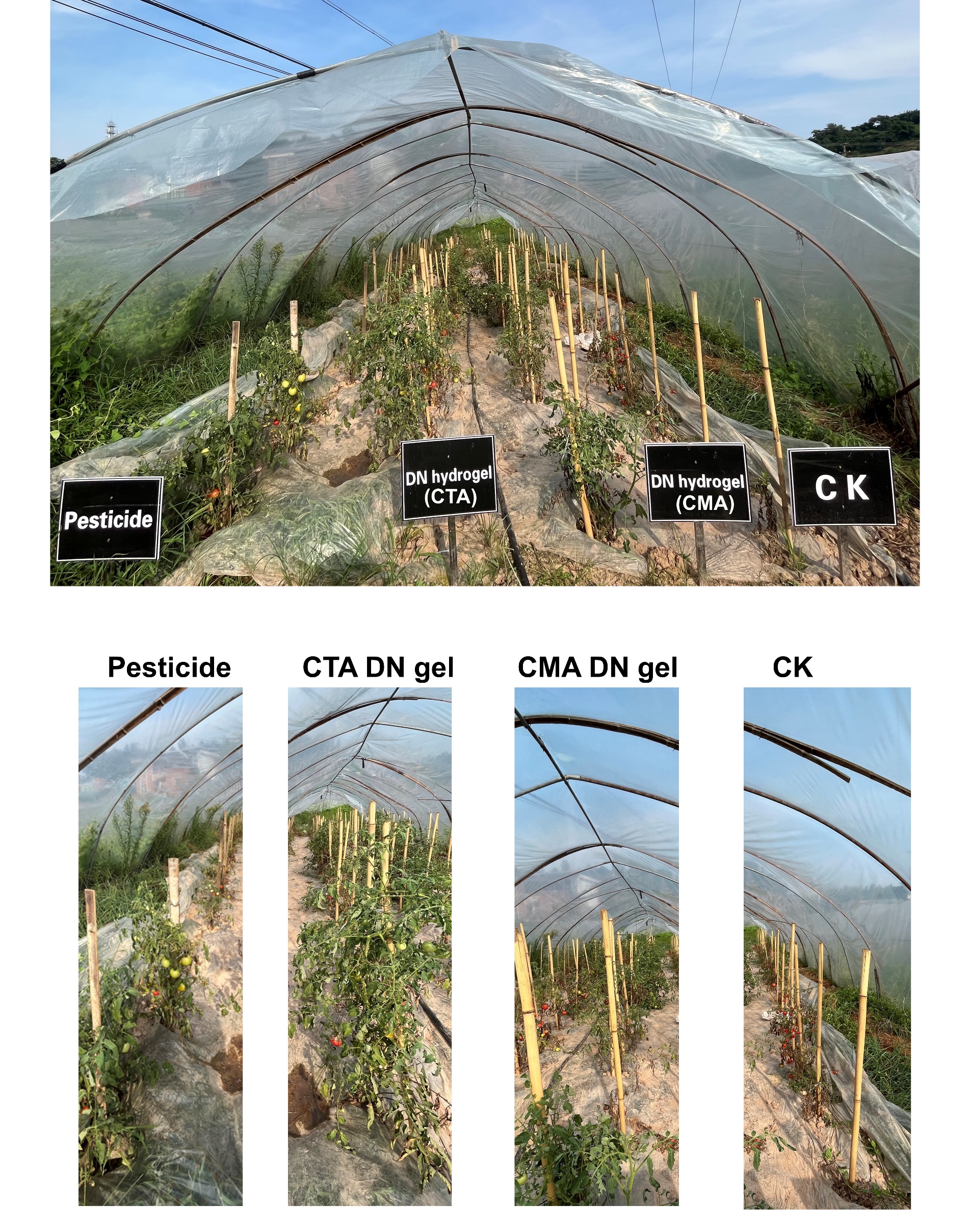


**Supplementary Figure 30:** Representative field photographs of tomato plants taken four weeks post-treatment with the pesticide, CMA DN gel, and CTA DN gel, illustrating the visual differences in plant health and disease progression among the treated groups.


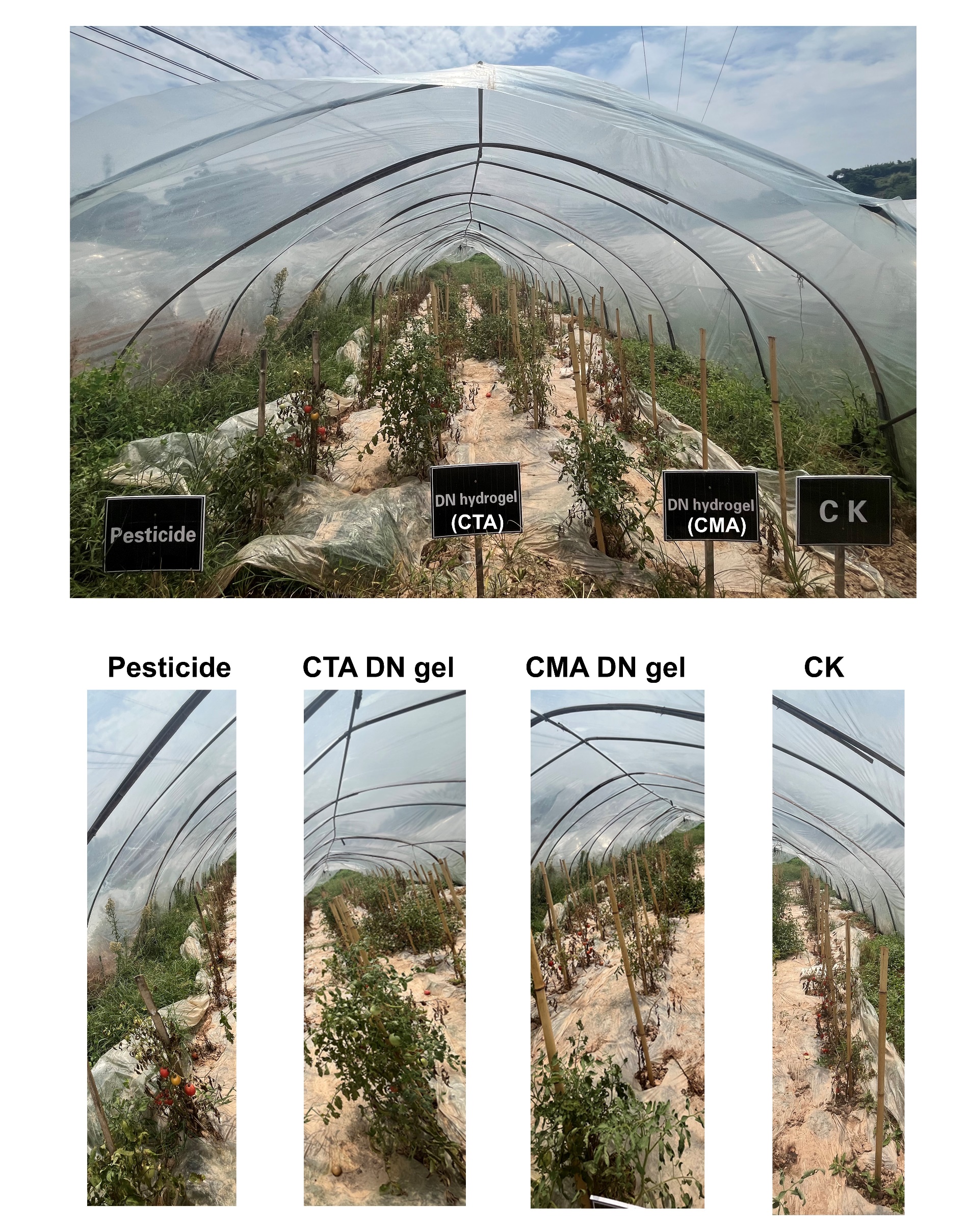


**Supplementary Figure 31:** Representative field photographs of tomato plants taken five weeks post-treatment with the pesticide, CMA DN gel, and CTA DN gel, illustrating the visual differences in plant health and disease progression among the treated groups.


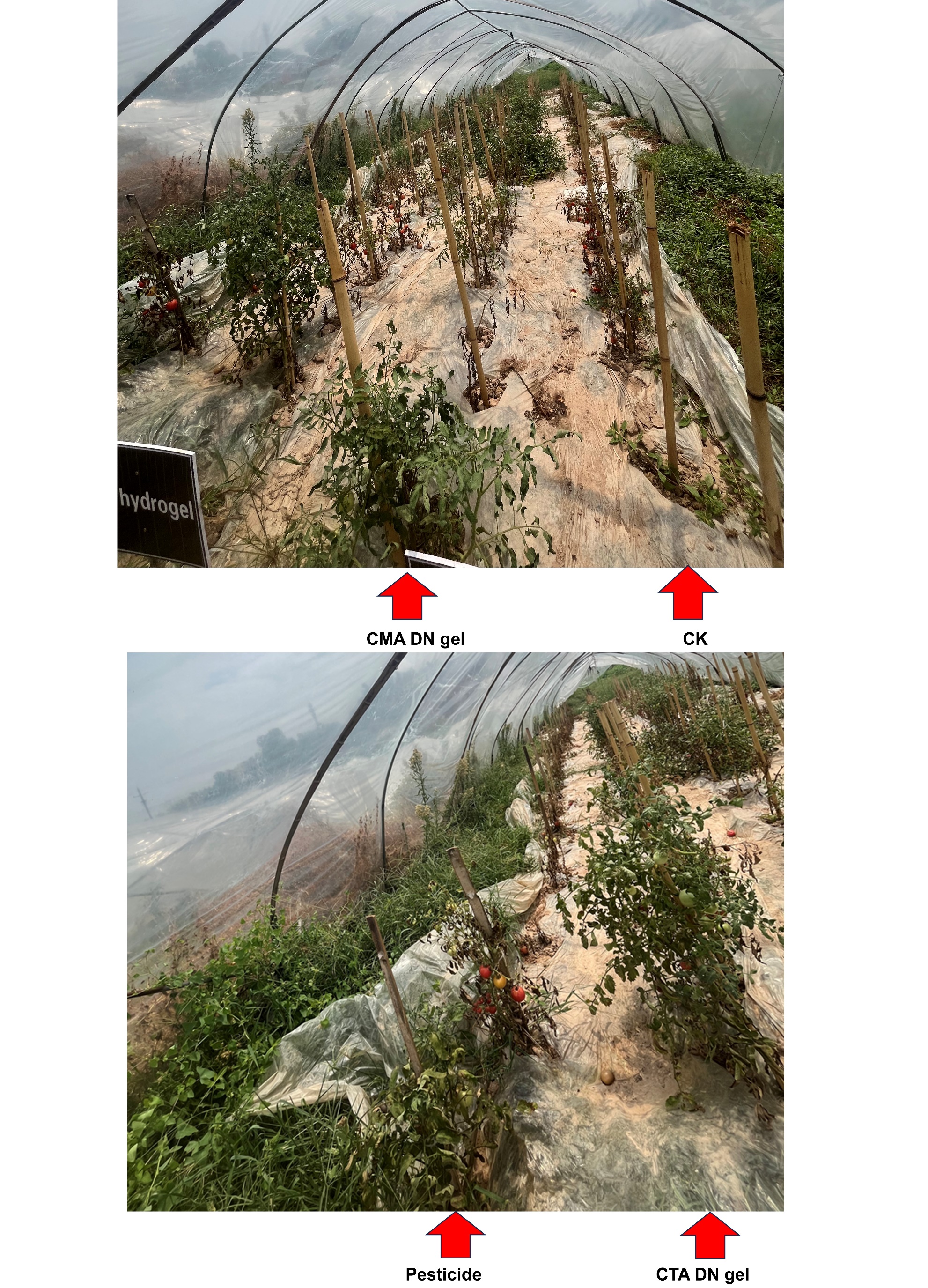


**Supplementary Figure 32:** Representative field photographs of tomato plants taken five weeks post-treatment with the pesticide, CMA DN gel, and CTA DN gel, illustrating the visual differences in plant health and disease progression among the treated groups.

**Field Experiment 2 (2025):**


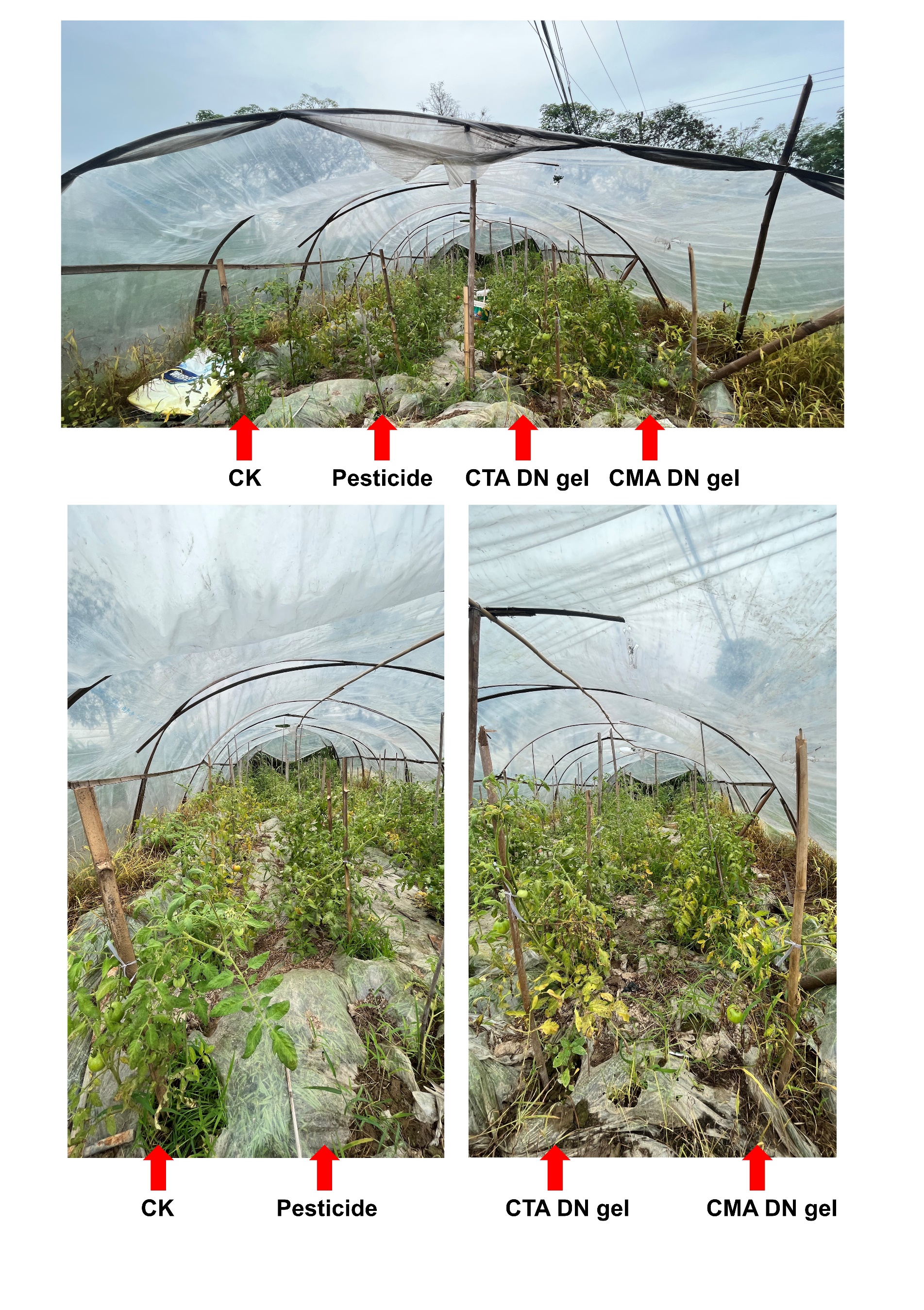


**Supplementary Figure 33:** Representative field photographs of tomato plants taken two weeks post-treatment with the pesticide, CMA DN gel, and CTA DN gel, illustrating the visual differences in plant health and disease progression among the treated groups.


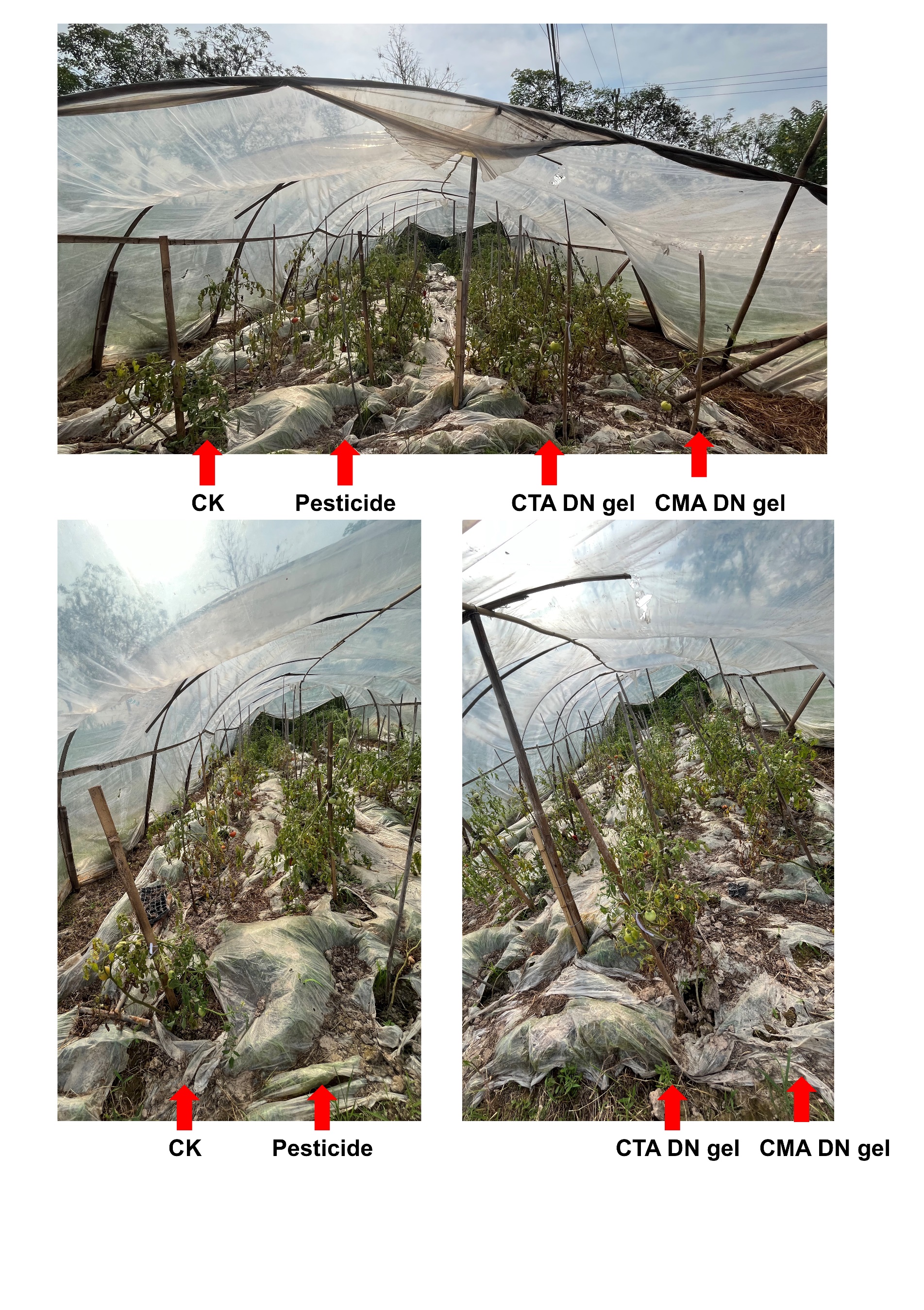


**Supplementary Figure 34:** Representative field photographs of tomato plants taken three weeks post-treatment with the pesticide, CMA DN gel, and CTA DN gel, illustrating the visual differences in plant health and disease progression among the treated groups.


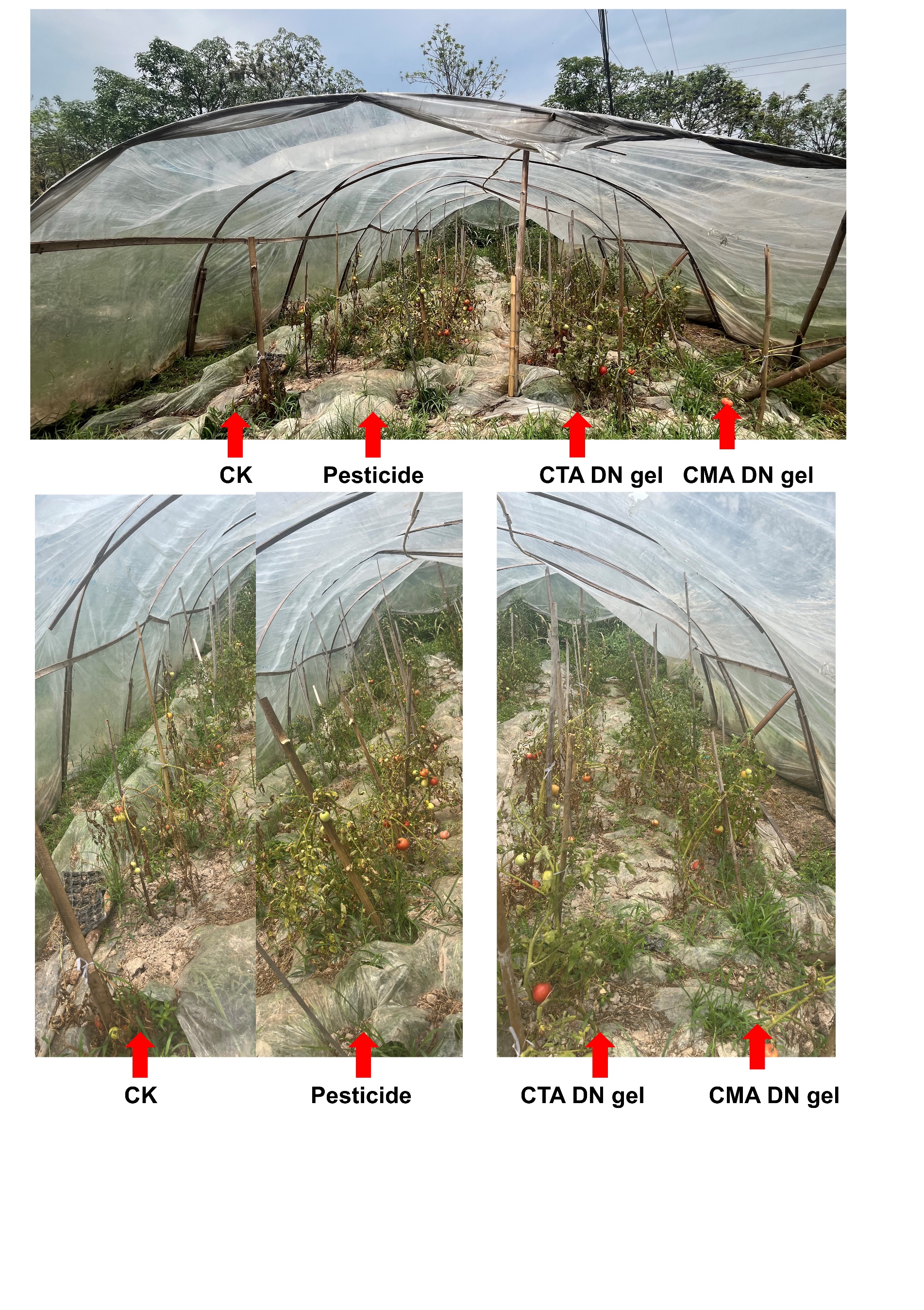


**Supplementary Figure 35:** Representative field photographs of tomato plants taken four weeks post-treatment with the pesticide, CMA DN gel, and CTA DN gel, illustrating the visual differences in plant health and disease progression among the treated groups.


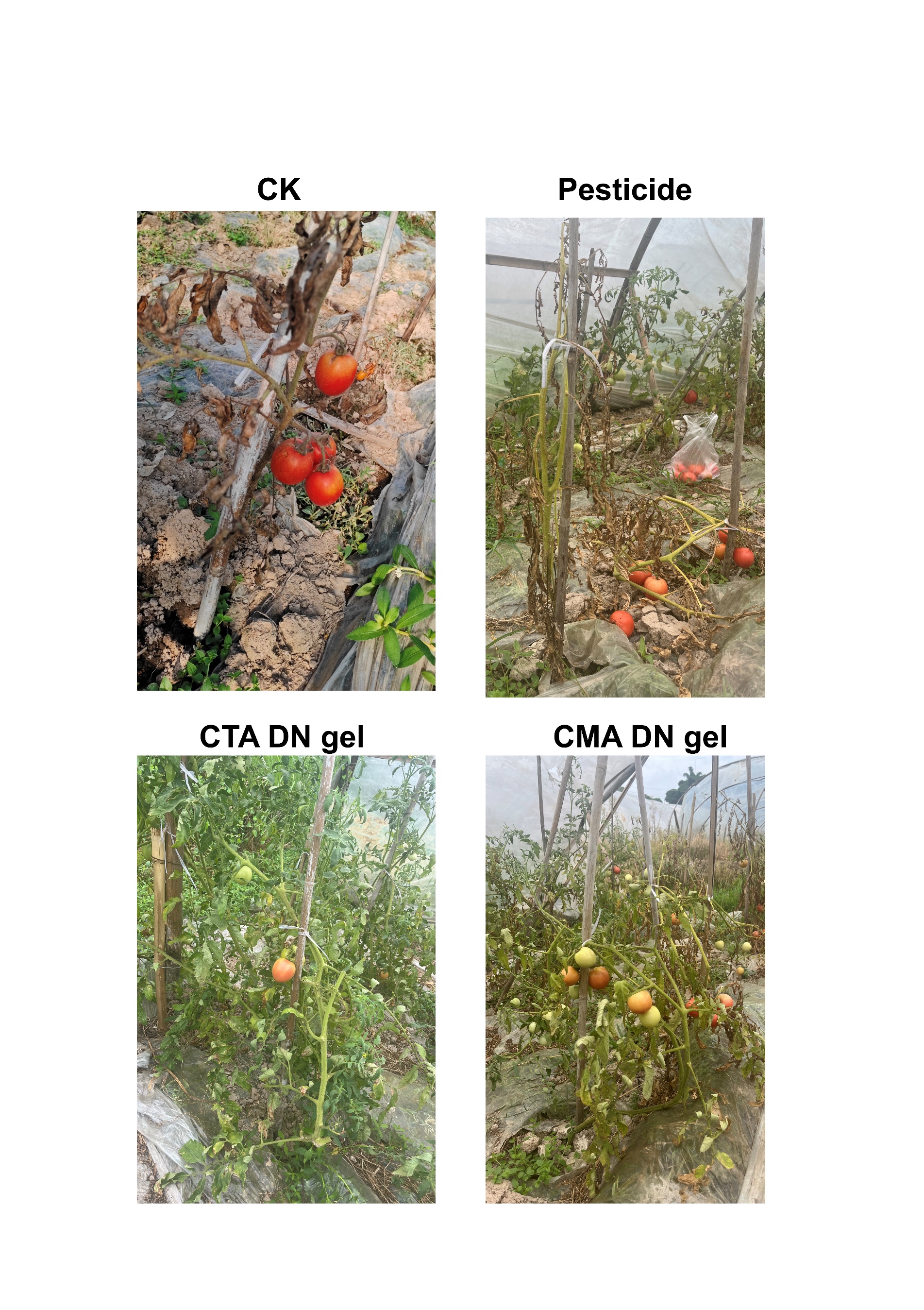


**Supplementary Figure 36:** Representative images showing the disease progression in tomato plants from the different treated groups, four weeks after field application of the CMA and CTA DN gels.


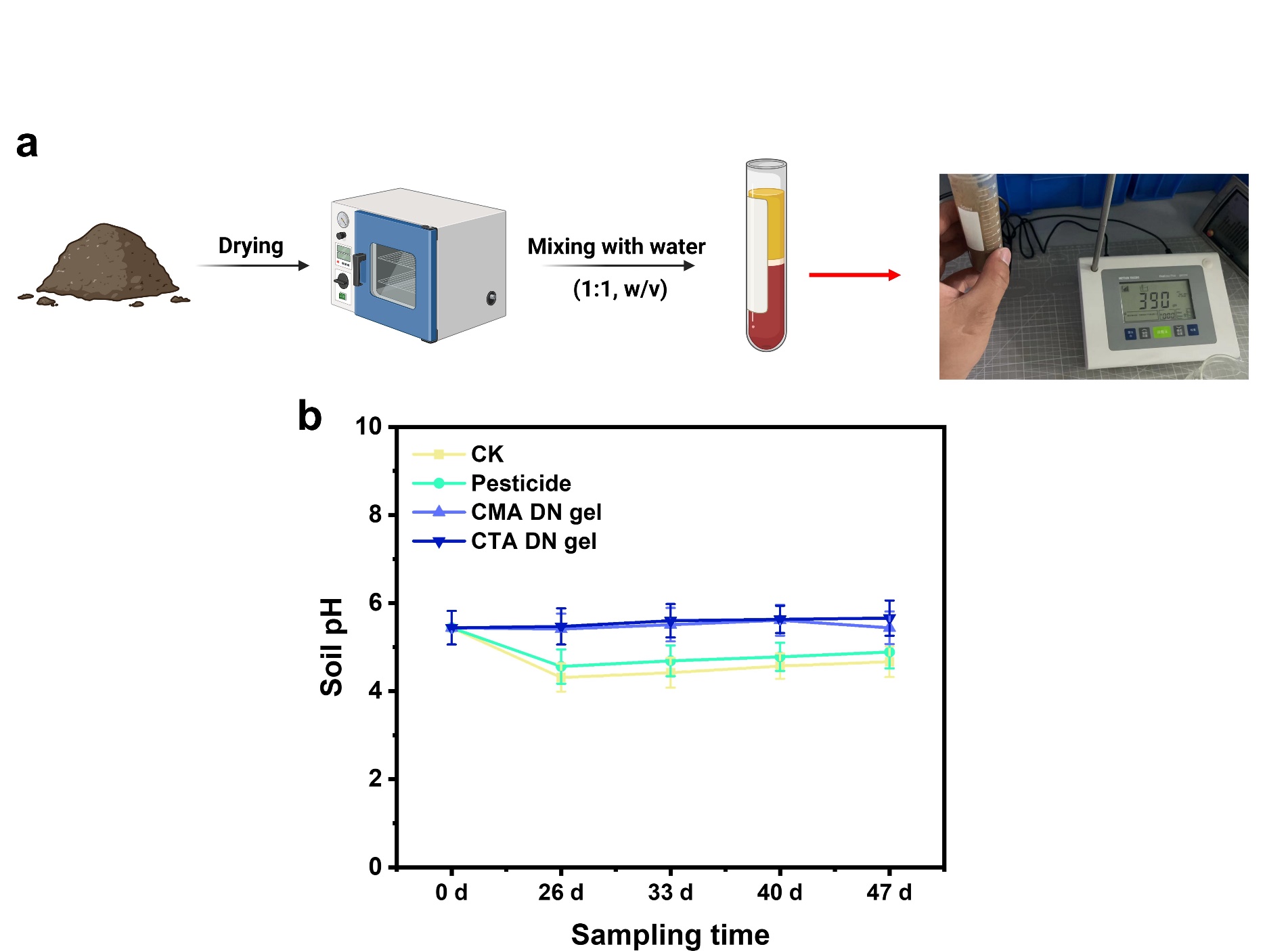


**Supplementary Figure 37:** (a) Schematic representation of the laboratory protocol used for soil pH measurement. (b) Time-course analysis of soil pH variation from day 0 (pre-treatment) to day 47 following the field application of DN gels. All measurements were performed with 6 technical replicates and 3 biological replicates for each group. Data are presented as mean ± SD (n = 3 independent experiments). The first three images in panel a (prior to the red arrows) were generated using BioRender, and the appropriate license has been obtained (Created in BioRender. Xiang, S. (2026) https://BioRender.com/5spydqm).


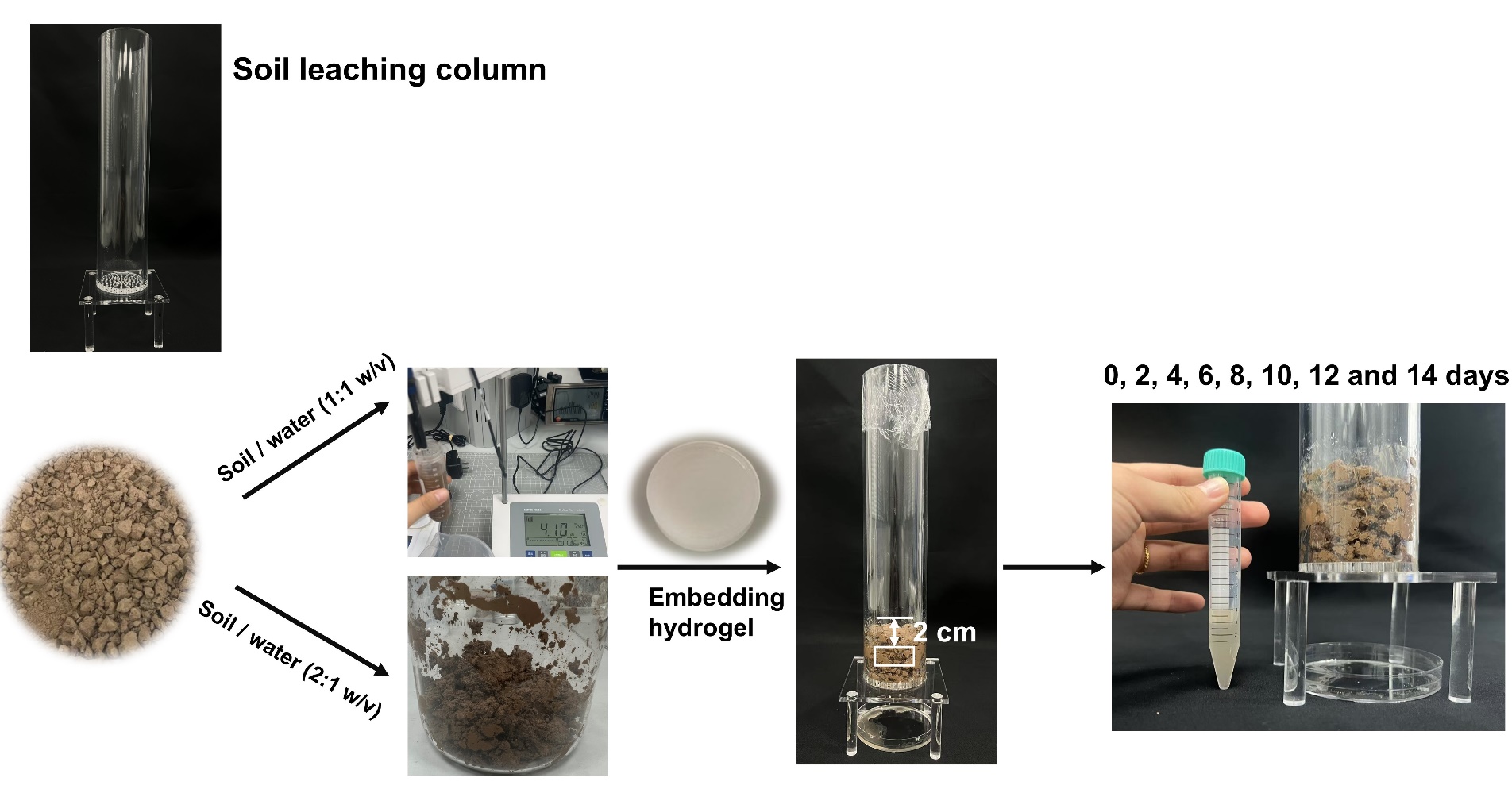


**Supplementary Figure 38:** Schematic illustration of the soil leaching apparatus designed to simulate the release dynamics of pesticides from the CMA or CTA DN gels under acidic (disease-affected) soil conditions. A total of 2 mL of the CMA or CTA DN gel was embedded in the soil matrix. Irrigation with 5 mL of water was conducted at day 0, 2, 4, 6, 8, 10, 12, and 14. Soil leachate samples were collected 2 h before each irrigation to quantify the concentration of released pesticide. The hydrogel was embedded in soil by placing a layer of soil at the bottom of the container, positioning the hydrogel block on top of this layer, and then covering it with an additional layer of soil.


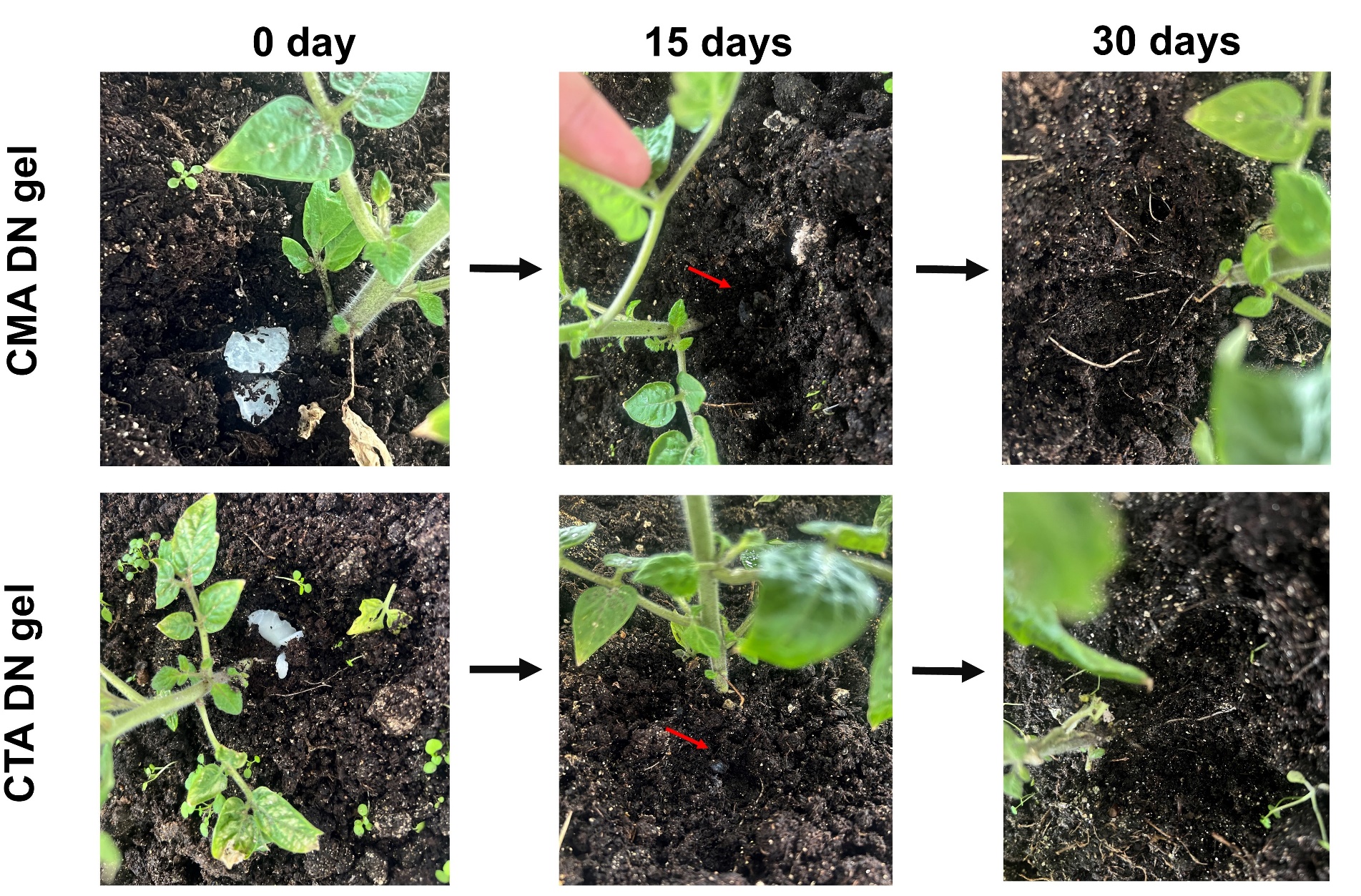


**Supplementary Figure 39:** Degradation process of the CMA DN gel and CTA DN gel blocks in soil.


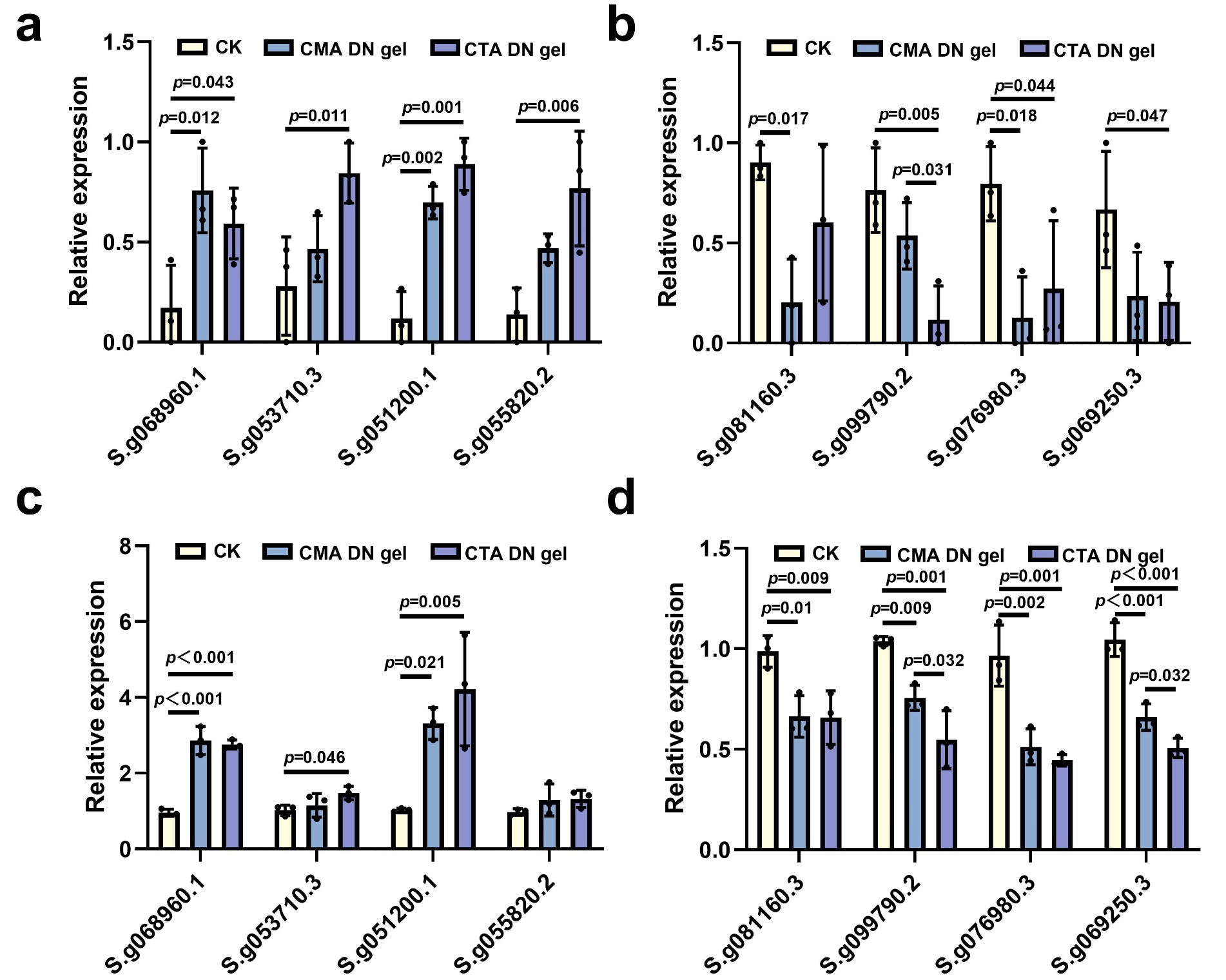


**Supplementary Figure 40:** Representative differentially expressed genes selected from the transcriptomic analysis: (a) upregulated genes and (b) downregulated genes. For panels a,b, all measurements were performed with 4 technical replicates. Data are presented as the mean of independent samples. qPCR validation of transcriptomic results: (c) upregulated genes and (d) downregulated genes. For panels c,d, all measurements were performed with 3 technical replicates and 3 biological replicates for each group. Data are presented as mean ± SD (n = 3 independent experiments). Statistical differences were determined using one-way ANOVA with post hoc test.

**Supplementary Table 1:** Evaluation of disease severity in tomato plants (pot experiments) following the treatment with water (CK group), Zn^2+^, the pesticide, pesticide+Zn^2+^, and the CMA/CTA DN gels. The disease index was assessed to quantify the extent of bacterial wilt symptoms. The distinct letters (a/b/c/d, A/B/C/D, *P* < 0.05) denote significant differences determined by one-way ANOVA with post hoc test. For each treatment, 6 replicate plants were used per experiment, with a total of 3 independent experimental repeats (n=3).

| **Group** | **Disease index**  **(7 days)** | **Disease index**  **(14 days)** |
| --- | --- | --- |
| **CK** | 83±7.7^d^ | 100^D^ |
| **Zn^2+^** | 33±3.5^c^ | 87.5±8.4^C^ |
| **Pesticide** | 25±2.9^bc^ | 79.2±8.1^BC^ |
| **Pesticide+Zn^2+^** | 20±2.4^b^ | 66.7±7.3^B^ |
| **CMA DN gel** | 0^a^ | 12.5±1.6^A^ |
| **CTA DN gel** | 0^a^ | 8.3±1.2^A^ |

**Supplementary Table 2:** Outdoor temperatures in Rongchang District, Chongqing, China, from June to August 2024 (data sourced from the China Meteorological Administration).

| **1-Jun** | **2-Jun** | **3-Jun** | **4-Jun** | **5-Jun** | **6-Jun** | **7-Jun** | **8-Jun** |
| --- | --- | --- | --- | --- | --- | --- | --- |
| 27°C | 24°C | 23°C | 22°C | 29°C | 30°C | 26°C | 23°C |
| **9-Jun** | **10-Jun** | **11-Jun** | **12-Jun** | **13-Jun** | **14-Jun** | **15-Jun** | **16-Jun** |
| 25°C | 30°C | 26°C | 32°C | 32°C | 34°C | 36°C | 32°C |
| **17-Jun** | **18-Jun** | **19-Jun** | **20-Jun** | **21-Jun** | **22-Jun** | **23-Jun** | **24-Jun** |
| 30°C | 29°C | 27°C | 29°C | 31°C | 29°C | 27°C | 26°C |
| **25-Jun** | **26-Jun** | **27-Jun** | **28-Jun** | **29-Jun** | **30-Jun** |  |  |
| 26°C | 25°C | 23°C | 28°C | 32°C | 34°C |  |  |
| **1-Jul** | **2-Jul** | **3-Jul** | **4-Jul** | **5-Jul** | **6-Jul** | **7-Jul** | **8-Jul** |
| 31°C | 34°C | 31°C | 33°C | 30°C | 35°C | 35°C | 31°C |
| **9-Jul** | **10-Jul** | **11-Jul** | **12-Jul** | **13-Jul** | **14-Jul** | **15-Jul** | **16-Jul** |
| 30°C | 28°C | 32°C | 36°C | 34°C | 32°C | 34°C | 34°C |
| **17-Jul** | **18-Jul** | **19-Jul** | **20-Jul** | **21-Jul** | **22-Jul** | **23-Jul** | **24-Jul** |
| 36°C | 33°C | 37°C | 33°C | 38°C | 38°C | 39°C | 38°C |
| **25-Jul** | **26-Jul** | **27-Jul** | **28-Jul** | **29-Jul** | **30-Jul** | **31-Jul** |  |
| 36°C | 38°C | 34°C | 28°C | 32°C | 33°C | 34°C |  |
| **1-Aug** | **2-Aug** | **3-Aug** | **4-Aug** | **5-Aug** | **6-Aug** | **7-Aug** | **8-Aug** |
| 37°C | 38°C | 37°C | 34°C | 35°C | 38°C | 39°C | 40°C |
| **9-Aug** | **10-Aug** | **11-Aug** | **12-Aug** | **13-Aug** | **14-Aug** | **15-Aug** | **16-Aug** |
| 39°C | 31°C | 37°C | 40°C | 41°C | 40°C | 39°C | 37°C |
| **17-Aug** | **18-Aug** | **19-Aug** | **20-Aug** | **21-Aug** | **22-Aug** | **23-Aug** | **24-Aug** |
| 37°C | 33°C | 28°C | 40°C | 40°C | 42°C | 42°C | 42°C |
| **25-Aug** | **26-Aug** | **27-Aug** | **28-Aug** | **29-Aug** | **30-Aug** | **31-Aug** |  |
| 41°C | 41°C | 42°C | 43°C | 41°C | 39°C | 40°C |  |

**Supplementary Table 3:** Outdoor temperatures in Rongchang District, Chongqing, China, from June to August 2025 (data sourced from the China Meteorological Administration).

| **1-Jun** | **2-Jun** | **3-Jun** | **4-Jun** | **5-Jun** | **6-Jun** | **7-Jun** | **8-Jun** |
| --- | --- | --- | --- | --- | --- | --- | --- |
| 23°C | 26°C | 28°C | 29°C | 30°C | 29°C | 32°C | 32°C |
| **9-Jun** | **10-Jun** | **11-Jun** | **12-Jun** | **13-Jun** | **14-Jun** | **15-Jun** | **16-Jun** |
| 32°C | 35°C | 36°C | 36°C | 38°C | 35°C | 34°C | 37°C |
| **17-Jun** | **18-Jun** | **19-Jun** | **20-Jun** | **21-Jun** | **22-Jun** | **23-Jun** | **24-Jun** |
| 39°C | 26°C | 32°C | 33°C | 26°C | 29°C | 31°C | 30°C |
| **25-Jun** | **26-Jun** | **27-Jun** | **28-Jun** | **29-Jun** | **30-Jun** |  |  |
| 25°C | 30°C | 34°C | 35°C | 30°C | 28°C |  |  |
| **1-Jul** | **2-Jul** | **3-Jul** | **4-Jul** | **5-Jul** | **6-Jul** | **7-Jul** | **8-Jul** |
| 32°C | 34°C | 34°C | 35°C | 27°C | 31°C | 27°C | 30°C |
| **9-Jul** | **10-Jul** | **11-Jul** | **12-Jul** | **13-Jul** | **14-Jul** | **15-Jul** | **16-Jul** |
| 26°C | 28°C | 29°C | 31°C | 32°C | 33°C | 35°C | 35°C |
| **17-Jul** | **18-Jul** | **19-Jul** | **20-Jul** | **21-Jul** | **22-Jul** | **23-Jul** | **24-Jul** |
| 36°C | 36°C | 32°C | 29°C | 32°C | 31°C | 29°C | 29°C |
| **25-Jul** | **26-Jul** | **27-Jul** | **28-Jul** | **29-Jul** | **30-Jul** | **31-Jul** |  |
| 32°C | 33°C | 32°C | 34°C | 37°C | 35°C | 38°C |  |
| **1-Aug** | **2-Aug** | **3-Aug** | **4-Aug** | **5-Aug** | **6-Aug** | **7-Aug** | **8-Aug** |
| 39°C | 40°C | 40°C | 40°C | 40°C | 39°C | 36C | 38°C |
| **9-Aug** | **10-Aug** |  |  |  |  |  |  |
| 38°C | 38°C |  |  |  |  |  |  |

**Supplementary Table 4:** Field evaluation of the disease incidence in tomato plants following the treatment with water (CK group), the pesticide, CMA DN gel, and CTA DN gel. The morbidity rate was recorded to assess the protective efficacy of each treatment against *R. solanacearum* infection under natural field conditions. The distinct letters (a/b/c, A/B/C, α/β/γ, x/y/z, *P* < 0.05) denote significant differences determined by one-way ANOVA with post hoc test. For each treatment, 30-40 replicate plants were used per experiment, with a total of 3 independent experimental repeats (n=3). These data are an integration of the field experiments conducted in 2024 and 2025.

| **Group** | **Morbidity rate (2^nd^ week)** | **Morbidity rate (3^rd^ week)** | **Morbidity rate (4^th^ week)** | **Morbidity rate (5^th^ week)** |
| --- | --- | --- | --- | --- |
| **CK** | 29.3±2.1%^c^ | 54.1±9.4%^C^ | 76.3±6.6%^γ^ | 81.2±7.5%^z^ |
| **Pesticide** | 19.0±2.6%^b^ | 37.3±6.1%^B^ | 54.6±8.7%^β^ | 63.2±6.8%^y^ |
| **CMA DN gel** | 10.1% ±2.0^a^ | 24.5±5.6%^A^ | 34.1±8.7%^α^ | 35.9±2.3%^x^ |
| **CTA DN gel** | 8.2±3.1%^a^ | 23.6±2.7%^A^ | 29.0±8.9%^α^ | 28.2±4.3%^x^ |

**Supplementary Table 5:** Field evaluation of the disease severity in tomato plants following the treatment with water (CK group), the pesticide, CMA DN gel, and CTA DN gel. The disease index was determined to quantify the extent of *R. solanacearum* infection and to evaluate the protective efficacy of each treatment under natural field conditions. The distinct letters, (a/b/c, A/B/C, α/β/γ, x/y/z, *P* < 0.05), denote significant differences determined by one-way ANOVA with post hoc test. For each treatment, 30-40 replicate plants were used per experiment, with a total of 3 independent experimental repeats (n=3). This table presents only the data from 2024.

| **Group** | **Disease index**  **(2^nd^ week)** | **Disease index**  **(3^rd^ week)** | **Disease index**  **(4^th^ week)** | **Disease index**  **(5^th^ week)** |
| --- | --- | --- | --- | --- |
| **CK** | 20.1±4.3^c^ | 32.5±5.8^C^ | 70.5±7.4^γ^ | 75.0±8.9^z^ |
| **Pesticide** | 12.5±3.4^b^ | 18.9±3.1^B^ | 45.3±6.7^β^ | 54.1±7.2^y^ |
| **CMA DN gel** | 5.8±1.6^a^ | 10.5±2.3^A^ | 18.8±4.1^α^ | 25.2±3.1^x^ |
| **CTA DN gel** | 3.4±1.4^a^ | 7.7＋1.2^A^ | 13.7±2.7^α^ | 19.9±3.1^x^ |

**Supplementary Table 6:** Assessment of the key agronomic parameters (plant height and width) in tomato plants cultivated under field conditions after application of the CMA or CTA DN gels. The distinct letters (a/b, A/B, *P* < 0.05) denote significant differences determined by one-way ANOVA with post hoc test. For each treatment, 30-40 replicate plants were used per experiment, with a total of 3 independent experimental repeats (n=3). This table presents only the data from 2024.

| **Group** | **Plant height (cm)** | **Plant width (cm)** |
| --- | --- | --- |
| **CK** | 75.3±3.5^a^ | 48±2.9^A^ |
| **Pesticide** | 79.9±4.2^a^ | 54.8±3.4^A^ |
| **CMA DN gel** | 107.1±6.4^b^ | 70.7±4.9^B^ |
| **CTA DN gel** | 113±7.1^b^ | 77.4±5.6^B^ |

**Supplementary Table 7:** Statistics of sequences after quality control in soil metagenomic analysis.

| **Samples** | **Clean reads** | **Clean base (bp)** | **Percent in raw reads (%)** | **Percent in raw bases (%)** |
| --- | --- | --- | --- | --- |
| **CK1** | 50188512 | 7525948432 | 99.9702 | 99.93928 |
| **CK2** | 48845408 | 7324649095 | 99.97052 | 99.94102 |
| **CK3** | 45285054 | 6790774318 | 99.97162 | 99.94242 |
| **CK4** | 50843734 | 7623915013 | 99.96699 | 99.93232 |
| **CMA1** | 46173352 | 6923774451 | 99.97049 | 99.93832 |
| **CMA2** | 43006562 | 6443153400 | 98.83441 | 98.71443 |
| **CMA3** | 41412142 | 6204523842 | 98.81482 | 98.69874 |
| **CMA4** | 44813642 | 6713938540 | 98.82877 | 98.70957 |
| **CTA1** | 41004744 | 6141868159 | 98.48445 | 98.34285 |
| **CTA2** | 46731902 | 6999117342 | 98.77134 | 98.62102 |
| **CTA3** | 39935180 | 5982915263 | 98.72801 | 98.60668 |
| **CTA4** | 39445752 | 5908490494 | 98.54077 | 98.40134 |

**Supplementary Table 8:** Statistical summary of gene prediction results from soil metagenomic data.

| **Sample** | **ORFs** | **Total length (bp)** | **Average length (bp)** | **Max (bp)** | **Min (bp)** |
| --- | --- | --- | --- | --- | --- |
| **CK1** | 470796 | 255396894 | 542.48 | 7593 | 102 |
| **CK2** | 506778 | 277479615 | 547.54 | 16011 | 102 |
| **CK3** | 484554 | 266317551 | 549.61 | 8463 | 102 |
| **CK4** | 460268 | 247215855 | 537.11 | 8499 | 102 |
| **CMA1** | 368852 | 187366566 | 507.97 | 4461 | 102 |
| **CMA2** | 369899 | 191509482 | 517.73 | 8454 | 102 |
| **CMA3** | 237608 | 120645630 | 507.75 | 7284 | 102 |
| **CMA4** | 365405 | 188858922 | 516.85 | 5637 | 102 |
| **CTA1** | 331106 | 169005516 | 510.43 | 5172 | 102 |
| **CTA2** | 419581 | 222929007 | 531.31 | 7728 | 102 |
| **CTA3** | 271772 | 138767352 | 510.6 | 5553 | 102 |
| **CTA4** | 303132 | 158590947 | 523.17 | 14457 | 102 |

**Supplementary Table 9:** LEfSe analysis of discriminative features. Statistical analyses were performed in R software using the non-parametric Kruskal-Wallis rank-sum test for multiple group comparisons. All tests were two-sided, and no adjustments for multiple comparisons were applied.

| Function name | Group | Mean | LDA_value | P value |
| --- | --- | --- | --- | --- |
| Erm(31) | CMA | 2.028344368 | 1.660854477 | 0.023069802 |
| Rhodococcus fascians cmr |  | 1.856783145 | 0 |  |
| opmE |  | 2.327070887 | 0 |  |
| MexC |  | 2.403379541 | 0 |  |
| cipA |  | 2.606827459 | 0 |  |
| Rhodobacter sphaeroides am... |  | 2.632776351 | 0 |  |
| mexX |  | 1.780139855 | 0 |  |
| FosX |  | 1.724049856 | 0 |  |
| bmr |  | 2.647025187 | 0 |  |
| AxyY | CMA | 2.695845712 | 2.002668989 | 0.023069802 |

**Supplementary Table 10:** Comparison of key properties of the CMA DN gel and CTA DN gel. The asterisk indicates a significant difference according to one-way ANOVA with post hoc test.

| Key indicators | CMA DN gel | CTA DN gel | P value^a^ |
| --- | --- | --- | --- |
| Strength (G') | 3748.9 Pa | **6865.4 Pa** | **0.004** |
| Pesticide release (pH=5) | 74.8% | 80.0% | 0.19 |
| Phe release (pH=5) | 47.0% | **56.2%** | **0.026** |
| Zn^2+^ release (pH=5) | 61.3% | **73.0%** | **0.02** |
| Yield (kg/plant) | 3.1 | 3.5 | 0.29 |

^a^P value < 0.05 is considered statistically significant.

**Supplementary Table 11:** Primers used in this study.

| **Primers’ names** | **Primers’ sequence** |
| --- | --- |
| *R. solanacearum-F* | 5'- GTCGCCGTCAACTCACTTTCC -3' |
| *R. solanacearum-R* | 5'- GTCGCCGTCAGCAATGCGGAATCG -3' |
| *Solyc06g068960.1-F* | 5'- CTAGGATCCGACACTTCGCC -3' |
| *Solyc06g068960.1-R* | 5'- CCAAGCGGTTCAGGATCTGA -3' |
| *Solyc12g099790.2-F* | 5'- GGGTTGTTGCTCAAAGGCAG -3' |
| *Solyc12g099790.2-R* | 5'- ATTGGGGCTGCCTTAGATGG -3' |
| *Solyc02g076980.3-F* | 5'- TCTCGATTGCACCACCAACA-3' |
| *Solyc02g076980.3-R* | 5'- TGTTTCACCTTCAGGCACGA -3' |
| *Solyc10g055820.2-F* | 5'- CATTTCCTGGCTTTGGCACC -3' |
| *Solyc10g055820.2-R* | 5'- AATTGGGCCTCGTCCGAAAT -3' |
| *Solyc06g053710.3-F* | 5'- TCATTCTCAGCCAGAGGGGA -3' |
| *Solyc06g053710.3-R* | 5'- GCAGTTGAAGCCCAAGAACG -3' |
| *Solyc05g051200.1-F* | 5'- ATGAGGGGTCCTTGGTCTCT -3' |
| *Solyc05g051200.1-R* | 5'- TCAACTCCCAAGTCTTGAAAAAC -3' |
| *Solyc01g081160.3-F* | 5'- TCAACGTGGTTTCGTGGGAT -3' |
| *Solyc01g081160.3-R* | 5'- TTGGAATTCTTGTCCCGGCA -3' |
| *Solyc02g069250.3-F* | 5'- GGAGTTGGTTGCATGTCAGC -3' |
| *Solyc02g069250.3-R* | 5'- GGTGCTGCTCTATCCATGGG -3' |

**Supplementary References**

1. Jeon, S. *et al*. Targeted delivery of sucrose-coated nanocarriers with chemical cargoes to the plant vasculature enhances long-distance translocation. Small. 20(7), 2304588 (2024).
2. Gomes, S. *et al*. Safe and sustainable by design Ag nanomaterials: A case study to evaluate the bio-reactivity in the environment using a soil model invertebrate. Sci. Total Environ., 927, 171860 (2024).
3. Bach-Pages, M. and Preston, G. Methods to quantify biotic-induced stress in plants. Host-pathogen interactions: methods and protocols. Springer New York. 241-255 (2017).
