# Supplementary material for: A pH-responsive double network hydrogel for control of tomato bacterial wilt
Source: Nat Commun. 2026 Jun 12;17:5219. doi: 10.1038/s41467-026-73922-3 (PMC13263331; doi:10.1038/s41467-026-73922-3)
Supplement: Supplementary file 2 — Description of Additional Supplementary Files [file 41467_2026_73922_MOESM2_ESM.pdf]

Description of Additional Supplementary Files

Supplementary Data 1: Differential gene statistics from soil metagenomic analysis.

Supplementary Data 2: Antibiotic Resistance Ontology abundance table from soil metagenomic analysis.
