# Supplementary material for: A pH-responsive double network hydrogel for control of tomato bacterial wilt
Source: Nat Commun. 2026 Jun 12;17:5219. doi: 10.1038/s41467-026-73922-3 (PMC13263331; doi:10.1038/s41467-026-73922-3)
Supplement: Supplementary file 5 — Reporting Summary [file 41467_2026_73922_MOESM5_ESM.pdf]

nature portfolio

Corresponding author(s): Alberto Bianco

Last updated by author(s): Apr 30, 2026

Reporting Summary

Nature Portfolio wishes to improve the reproducibility of the work that we publish. This form provides structure for consistency and transparency in reporting. For further information on Nature Portfolio policies, see our Editorial Policies and the Editorial Policy Checklist.

Statistics

For all statistical analyses, confirm that the following items are present in the figure legend, table legend, main text, or Methods section.

n/a	Confirmed	
☐	☒	The exact sample size (*n*) for each experimental group/condition, given as a discrete number and unit of measurement
☐	☒	A statement on whether measurements were taken from distinct samples or whether the same sample was measured repeatedly
☐	☒	The statistical test(s) used AND whether they are one- or two-sided<br>*Only common tests should be described solely by name; describe more complex techniques in the Methods section.*
☒	☐	A description of all covariates tested
☒	☐	A description of any assumptions or corrections, such as tests of normality and adjustment for multiple comparisons
☐	☒	A full description of the statistical parameters including central tendency (e.g. means) or other basic estimates (e.g. regression coefficient) AND variation (e.g. standard deviation) or associated estimates of uncertainty (e.g. confidence intervals)
☐	☒	For null hypothesis testing, the test statistic (e.g. *F*, *t*, *r*) with confidence intervals, effect sizes, degrees of freedom and *P* value noted<br>*Give P values as exact values whenever suitable.*
☒	☐	For Bayesian analysis, information on the choice of priors and Markov chain Monte Carlo settings
☒	☐	For hierarchical and complex designs, identification of the appropriate level for tests and full reporting of outcomes
☒	☐	Estimates of effect sizes (e.g. Cohen's *d*, Pearson's *r*), indicating how they were calculated

Our web collection on statistics for biologists contains articles on many of the points above.

Software and code

Policy information about availability of computer code

Data collection	The data were collected on Frontier™ spectrometer (PerkinElmer, FTIR), Zetasizer (Malvern Panalytical, zeta potential), Avance III 500 MHz spectrometer (Bruker, NMR), Regulus8220 (cryo-SEM), SU8020 and TEM systems (Hitachi High-Tech, SEM/TEM), and an MCR 702 e rheometer (Anton Paar, rheology).
Data analysis	Data were analyzed using IBM SPSS Statistics 26.0, Origin 2021, GraphPad Prism 9.0.0 and Microsoft Excel 2016, while fluorescent images were quantitatively analyzed using ImageJ 6.0.

For manuscripts utilizing custom algorithms or software that are central to the research but not yet described in published literature, software must be made available to editors and reviewers. We strongly encourage code deposition in a community repository (e.g. GitHub). See the Nature Portfolio guidelines for submitting code & software for further information.

Data

Policy information about availability of data

All manuscripts must include a data availability statement. This statement should provide the following information, where applicable:

- Accession codes, unique identifiers, or web links for publicly available datasets
- A description of any restrictions on data availability
- For clinical datasets or third party data, please ensure that the statement adheres to our policy

The soil metagenomic and tomato RNA sequencing data generated in this study have been deposited in the NCBI database under accession code: PRJNA1445876

(https://www.ncbi.nlm.nih.gov/search/all/?term=PRJNA1445876+), PRJNA1271282 (https://www.ncbi.nlm.nih.gov/search/all/?term=PRJNA1271282). All other analyses and data generated in this study are provided in the Supplementary Infor-mation/Source Data file. Source data is available for Figures 3i-n, 4a,b,e, 5c-e, 6b,c, 7c,d, 8e and 9a-c, 9k, 9l, Supplementary Figures 3, 13, 14, 15a-d, 16, 17a,c, 22, 23, 24c-f, 25b-e, 26b-e, 27, 37b and 40 and Supplementary Tables 1, 4, 5, 6 and 10 in the associated source data file. The source data are provided with this article

Research involving human participants, their data, or biological material

Policy information about studies with human participants or human data. See also policy information about sex, gender (identity/presentation), and sexual orientation and race, ethnicity and racism.

Reporting on sex and gender	n/a
Reporting on race, ethnicity, or other socially relevant groupings	n/a
Population characteristics	n/a
Recruitment	n/a
Ethics oversight	n/a

Note that full information on the approval of the study protocol must also be provided in the manuscript.

Field-specific reporting

Please select the one below that is the best fit for your research. If you are not sure, read the appropriate sections before making your selection.

☒ Life sciences ☐ Behavioural & social sciences ☐ Ecological, evolutionary & environmental sciences

For a reference copy of the document with all sections, see nature.com/documents/nr-reporting-summary-flat.pdf

Life sciences study design

All studies must disclose on these points even when the disclosure is negative.

Sample size	For all experiments, each treatment group included at least three technical replicates and a minimum of three independent biological replicates to ensure the reliability of the results.
Data exclusions	No data exclusion.
Replication	All experimental results are reproducible
Randomization	All biological samples were randomly assigned to experimental groups to minimize potential bias and eliminate subjective interference.
Blinding	Blinding was implemented during data collection and analysis to minimize subjective bias.

Reporting for specific materials, systems and methods

We require information from authors about some types of materials, experimental systems and methods used in many studies. Here, indicate whether each material, system or method listed is relevant to your study. If you are not sure if a list item applies to your research, read the appropriate section before selecting a response.

Materials & experimental systems

n/a	Involved in the study	
☒	☐	Antibodies
☐	☒	Eukaryotic cell lines
☒	☐	Palaeontology and archaeology
☒	☐	Animals and other organisms
☒	☐	Clinical data
☒	☐	Dual use research of concern
☐	☒	Plants

Methods

n/a	Involved in the study	
☒	☐	ChIP-seq
☒	☐	Flow cytometry
☒	☐	MRI-based neuroimaging

Eukaryotic cell lines

Policy information about cell lines and Sex and Gender in Research

Cell line source(s)	R. solanacearum and R. solanacearum carrying green fluorescent protein (R. sola-nacearum-GFP) were obtained from Prof. Wang's laboratory at the College of Re-sources and Environment, Southwest University, China.
Authentication	No relevant authentication information is available.
Mycoplasma contamination	There is no Mycoplasma contamination.
Commonly misidentified lines (See ICLAC register)	n/a

Plants

Seed stocks	The tomato seeds (DRK0568) were sourced from Seminis Seeds (Beijing) Co., Ltd.
Novel plant genotypes	n/a
Authentication	n/a
