# Supplementary material for: A pH-responsive double network hydrogel for control of tomato bacterial wilt
Source: Nat Commun. 2026 Jun 12;17:5219. doi: 10.1038/s41467-026-73922-3 (PMC13263331; doi:10.1038/s41467-026-73922-3)
Supplement: Supplementary file 6 — Transparent Peer Review file [file 41467_2026_73922_MOESM6_ESM.pdf]

nature portfolio

Peer Review File

A pH-Responsive Double Network Hydrogel for Control of Tomato Bacterial Wilt

Corresponding Author: Dr Alberto Bianco

This file contains all reviewer reports in order by version, followed by all author rebuttals in order by version.

Version 0:

Reviewer comments:

Reviewer #1

(Remarks to the Author)
This manuscript submitted by Xiang et al. presented a well-designed and comprehensive study on the development of dual pH-responsive double-network hydrogels for the targeted and sustainable management of tomato bacterial wilt caused by Ralstonia solanacearum. The work is highly innovative, combining material science with plant pathology to address a significant agricultural problem. The results are clearly presented and strongly support the conclusions. The topic is interesting and within the scope of Nature Communications. However, there are some other problems should be addressed.
1.Although the hydrogel shows stability over 30 days in field conditions, a more detailed discussion on its biodegradation timeline and long-term ecological impact would strengthen the environmental safety claim.
2.A brief comparative discussion with other pH-responsive systems (e.g., MOFs, nanoparticles) in the Introduction or Discussion could better contextualize the novelty of the current work.
3.The authors note that CTA performs better due to more carboxyl groups and simpler synthesis. A side-by-side comparison table in the supplementary information summarizing the key differences (e.g., release rate, mechanical strength, cost) would be helpful.
4.Figure 4a-d is not sufficiently clear.

Reviewer #2

(Remarks to the Author)
This manuscript describes a dual pH-responsive double-network (DN) hydrogel designed for the control of bacterial wilt caused by Ralstonia solanacearum. While the study integrates material design, antibacterial performance, and field testing, several key conceptual and mechanistic claims are not sufficiently supported by experimental evidence. In particular, the rationale for the material design, the definition of the “double-network” structure, the claimed targeting behavior, and the assessment of biosafety and ecological impact remain inadequate for the standards of Nature Communications. Substantial additional work would be required to address these concerns.
1.The manuscript employs both CTA and CMA hydrogels in a double-network structure, yet fails to provide a convincing scientific rationale for why both are necessary. Their respective advantages, functional distinctions, and synergistic contributions are not clearly articulated.
2.The choice of L-phenylalanine among numerous amino acids is not convincingly justified. The manuscript does not clearly explain its unique advantages in terms of self-assembly, bioactivity, or coordination chemistry compared with other amino acids. More importantly, although Phe–Zn^{2+} is claimed to form a secondary network, there is no direct experimental evidence or characterization demonstrating its interaction or crosslinking with the CMA/CTA hydrogel system.
3.The scale bars in Figure 2b–h are unclear. Furthermore, the drastic morphological changes observed after introducing the Phe–Zn^{2+} system are not adequately explained. The origin, composition, and formation mechanism of the observed surface film-like structure remain speculative and unsupported by experimental analysis.
4.Although the hydrogel is repeatedly described as a “double-network” system, the manuscript does not provide convincing evidence to support this claim. There is a lack of structural, mechanical, or network-specific characterization demonstrating the coexistence of two independent yet interpenetrating networks. Without such evidence, the DN designation appears overstated.
5.The antibacterial activity of the CMA/CTA DN hydrogel appears to be largely non-selective, driven primarily by the release of Zn^{2+} and a broad-spectrum pesticide. The manuscript does not address the potential negative impacts on beneficial soil

microbiota during field application. Moreover, the risks of long-term use, including bacterial resistance development and environmental contamination, are not meaningfully discussed or experimentally evaluated.
6.The manuscript claims that the hydrogel is “targeted” toward R. solanacearum, yet no specific targeting mechanism is demonstrated. The system appears to rely solely on environmental pH responsiveness, which is a general stimulus rather than a true biological targeting strategy. The absence of defined molecular, cellular, or microenvironmental targets undermines the validity of the targeting claim.
7.The manuscript does not clearly state how many days after treatment the observations in Figure 4c were conducted. This lack of essential experimental detail compromises data reproducibility and interpretation.
8.In Figure 5c, seedling length under acidic conditions (pH = 5) does not appear to be clearly superior to that under neutral conditions (pH = 7). This weak or ambiguous difference does not convincingly support the claimed pH-responsive growth enhancement.
9.The biosafety assessment is incomplete. Key agricultural indicators such as pollen germination rate and fruit set are not evaluated. Without assessing reproductive parameters, the manuscript does not sufficiently demonstrate that the hydrogel is safe for long-term agricultural use.
10.All tomato plant images presented in the manuscript lack scale bars, preventing quantitative or comparative evaluation of plant growth and morphology.
11.The release profiles in Figure 6d indicate that free pesticide exhibits rapid and substantial release within the first few days, exceeding that of CMA DN and CTA DN. This raises the question of whether the hydrogel system provides a meaningful advantage over conventional pesticide application. Furthermore, the relatively low initial release (<20%) from CMA DN and CTA DN casts doubt on whether sufficient antibacterial concentrations are achieved.
12.The transcriptomic data lack experimental validation, such as real-time PCR. Additionally, the analysis is superficial, focusing only on pathway enrichment without clearly distinguishing between upregulated and downregulated genes. This significantly limits the mechanistic insight into plant immune responses and weakens the biological conclusions.

Reviewer #3

(Remarks to the Author)
I co-reviewed this manuscript with one of the reviewers who provided the listed reports. This is part of the Nature Communications initiative to facilitate training in peer review and to provide appropriate recognition for Early Career Researchers who co-review manuscripts.

Version 1:

Reviewer comments:

Reviewer #1

(Remarks to the Author)
All the comments have been carefully addressed. Accept this as it is.

Reviewer #2

(Remarks to the Author)
The authors have carefully and comprehensively addressed the comments raised in the first round of review. The revisions are thorough and well justified, and the overall quality of the manuscript has been significantly improved. The additional analyses, clarifications, and improved presentation have strengthened the scientific rigor and clarity of the work.

In its current form, the manuscript meets the standard for publication, and I have no further major concerns.

Reviewer #3

(Remarks to the Author)
I co-reviewed this manuscript with one of the reviewers who provided the listed reports. This is part of the Nature Communications initiative to facilitate training in peer review and to provide appropriate recognition for Early Career Researchers who co-review manuscripts.

Open Access This Peer Review File is licensed under a Creative Commons Attribution 4.0 International License, which permits use, sharing, adaptation, distribution and reproduction in any medium or format, as long as you give appropriate credit to the original author(s) and the source, provide a link to the Creative Commons license, and indicate if changes were made.
In cases where reviewers are anonymous, credit should be given to 'Anonymous Referee' and the source.
The images or other third party material in this Peer Review File are included in the article's Creative Commons license, unless indicated otherwise in a credit line to the material. If material is not included in the article's Creative Commons license and your intended use is not permitted by statutory regulation or exceeds the permitted use, you will need to obtain permission directly from the copyright holder.
To view a copy of this license, visit https://creativecommons.org/licenses/by/4.0/

Point-to-point responses to the Editorial Office:

Editorial Comments:

Comment 1. Please follow all reporting standards below:
Please complete or update the following checklist(s) to verify compliance with our research ethics and data reporting standards. Address all points on the checklist, revising your manuscript in response to the points if needed.
The form(s) must be downloaded and completed in Adobe Reader rather than opened in a web browser. Each form must be uploaded as a Related Manuscript file at the time of resubmission.
Reporting summary:
https://www.nature.com/documents/nr-reporting-summary.pdf
*** Your work characterises chemical or biomolecular materials. Please see the link below for reporting requirements. There is no form to upload but you may need to revise your manuscript to comply with this policy.**
https://www.nature.com/ncomms/submit/chemical-characterisation

Response: Thank you for this explanation. We have completed the required reporting checklist in accordance with the guidelines and uploaded it as requested. In addition, we have carefully reviewed the reporting requirements for chemical and biomolecular material characterization and have revised the manuscript where necessary to ensure that all material characterization complies with these standards.

Comment 2. All Nature Communications manuscripts must include a "Data Availability" section after the Methods section but before the References. If any of the data can only be shared on request or are subject to restrictions, please specify the reasons and explain how, when, and by whom the data can be accessed. For more information on this policy and a list of examples, see: https://www.nature.com/documents/nr-data-availability-statements-data-citations.pdf

Response: Thank you for the suggestion. We have revised the "Data Availability" section accordingly and now state that the data are available from the authors upon reasonable request.

Comment 3. As Nature Portfolio policies strongly encourages you to share your research data in a public repository (e.g. spreadsheets, text, images), we are partnering with the figshare repository so that you can use the figshare integration via the 'Research Data Deposition' tab when submitting your revised manuscript.
Data are stored privately until a manuscript decision is reached and you can edit/withdraw them up to this point: you retain rights and control over your data. The data will be published at the same time as your article; you will receive a data DOI, with guidance on linking the data and manuscript. In the event your manuscript is not accepted, you can keep or remove your data in figshare.

We recommend the use of discipline-specific repositories where available and for a number of data types this is mandatory. Ensure you do not submit these data types or any sensitive data to figshare.

Response: Thank you for the helpful suggestion. We have organized the raw data into an Excel file, named Source Data, and uploaded it along with the revised manuscript as requested.

Comment 4. We strongly encourage you to deposit all new data associated with the paper in a persistent repository where they can be freely and enduringly accessed. We recommend submitting the data to discipline-specific and community-recognised repositories; a list of repositories is provided here: http://www.nature.com/sdata/policies/repositories Refer to our data policies here: https://www.nature.com/nature-portfolio/editorial-policies/reporting-standards#availability-of-data.

Response: Thank you for the suggestion. As mentioned above, all newly generated data during the revision have been compiled into the "Source Data" file and submitted to the journal in accordance with the requirements.

Comment 5. Proteomic datasets must be deposited in a publicly accessible database, and accession codes and associated hyperlinks must be provided in the 'Data Availability' section.
All DNA sequencing, RNA sequencing or microarray data must be deposited in an approved, publicly accessible repository listed here (https://www.nature.com/nature-research/editorial-policies/reporting-standards#availability-of-data), and relevant accession codes should be stated in the data availability statement.

Response: Thank you for the guidance. We have deposited all sequencing data in the NCBI database in accordance with the requirements, and the relevant accession codes have been included in the 'Data Availability' section.

Comment 6. To maximise the reproducibility of research data, we ask that you provide a Source Data file containing the raw data underlying the following types of display items:
- Any reported means/averages in box plots, bar charts, and tables
- Dot plots/scatter plots, especially when there are overlapping points
- Line graphs
- Uncropped and unprocessed scans of all blots and gels including all quantified replicates. The edge of membranes, molecular weight ladders and loading controls should be presented on all blots. Where membranes have been cut, please ensure that at least one marker above and below is present. For an example of presenta-

tion of full scan blots, see the Source Data file of https://www.nature.com/articles/s41467-020-16984-1#Sec35 and for more information, please refer to https://www.nature.com/nature-research/editorial-policies/image-integrity.
The data should be provided in a single Excel file with data for each figure/table in a separate sheet, or in multiple labelled files within a zipped folder. Name this file or folder 'Source Data', and include a brief description in your cover letter. The 'Data Availability' section should also include the statement 'Source Data are provided with this paper.'.

Response: Thank you for the guidance. We have provided all the required raw data and compiled them into a single Excel file as requested. In addition, we have included the statement 'Source Data are provided with this paper' in the 'Data Availability' section.

Comment 7. Please replace your bar graphs with plots that feature information about the distribution of the underlying data. All data points should be shown for plots with a sample size less than 10. For larger sample sizes, please consider box-and-whisker or violin plots as alternatives. Measures of centrality, dispersion and/or error bars should be plotted and described in the figure legend.

Response: Thank you for the suggestion. We have revised all bar graphs accordingly to clearly present the distribution of the underlying data, in line with the journal's requirements.

Point-to-point responses to the Reviewers:

Reviewer #1:

General comment: This manuscript submitted by Xiang et al. presented a well-designed and comprehensive study on the development of dual pH-responsive double-network hydrogels for the targeted and sustainable management of tomato bacterial wilt caused by *Ralstonia solanacearum*. The work is highly innovative, combining material science with plant pathology to address a significant agricultural problem. The results are clearly presented and strongly support the conclusions. The topic is interesting and within the scope of Nature Communications. However, there are some other problems should be addressed.

Response: Thank you very much for your positive and encouraging evaluation of our manuscript. We sincerely appreciate your recognition of the novelty and significance of our work. In response to your valuable comments, we have carefully revised the manuscript to further improve its quality and clarity.

Comment 1. Although the hydrogel shows stability over 30 days in field conditions, a more detailed discussion on its biodegradation timeline and long-term ecological impact would strengthen the environmental safety claim.

Response 1: We thank the reviewer for this insightful suggestion. To better address this concern, we conducted additional experiments to further evaluate the biodegradation behavior of the hydrogels under soil conditions. Specifically, tomato plants were re-treated with CMA/CTA DN hydrogels, and the degradation process in soil was continuously monitored. As shown in the new **Supplementary Figure 39**, compared to the initial state, both CMA and CTA DN hydrogels exhibited a noticeable reduction in volume after 15 days, with no visible hydrogel residues remaining in the soil at 30 days, indicating near complete degradation. These observations suggest that the degradation period of the hydrogels is approximately 30 days. Importantly, previous studies have reported the presence of agar-degrading microorganisms in agricultural soils (Ikenaga et al., *Microbes Environ*. 2021, 36(1), ME20136; Lakshmikanth et al., *Process Biochem*. 2009, 44(9), 999-1003.), which are capable of facilitating the breakdown of polysaccharides such as agarose. As CMA and CTA DN hydrogels are primarily composed of agarose, these microorganisms are likely to further promote their degradation in soil environments.

To further evaluate the long-term ecological impact, we collected soil samples from field trials 7 months after CMA/CTA DN hydrogel application and performed metagenomic sequencing to systematically assess their effects on soil microbial communities. During this period, the soils were preserved without additional crop cultivation or agricultural disturbance (e.g., tillage or herbicide application), minimizing external influences on microbial activity. The results showed that the hydrogel treatment reshaped the microbial community structure. However, it is important to emphasize that no significant reduction in sequencing depth, gene richness, or assembly quality was observed. The dominant microbial phyla remained present across all treatments, and no abnormal bloom or collapse of major microbial taxa was detected. Therefore, the observed changes represent community restructuring rather than ecological disruption. Overall,

our results demonstrate that CMA and CTA DN hydrogels do not induce detectable ecological destabilization or increased resistance risk within the experimental timeframe. Nevertheless, we acknowledge that potential long-term effects or risks associated with repeated applications were not fully addressed in this study. Accordingly, we have revised the Discussion section to (i) highlight the necessity of long-term and repeated-application assessments to fully evaluate the environmental safety and application potential of CMA/CTA DN hydrogels, and (ii) compare the ecological impacts observed in this study with the known resistance risks associated with conventional chemical bactericides (For a detailed response regarding this section, please refer to our reply to Reviewer 2's comment 5).

To address these points, the following sentences have been added to the revised manuscript (line 672-678):

"*Simultaneously, soil-based degradation experiments further confirmed these observations (***Supplementary *Figure 39****), demonstrating that CMA DN gels and CTA DN gels remain observable by naked eye in soil for approximately one month. A clear, gradual degradation of the hydrogels was monitored, and this process facilitates the sustained release of the encapsulated pesticide. Moreover, the slow degradation also guarantees prolonged retention of the active agent at the site of disease occurrence, supporting extended bioactivity.*"

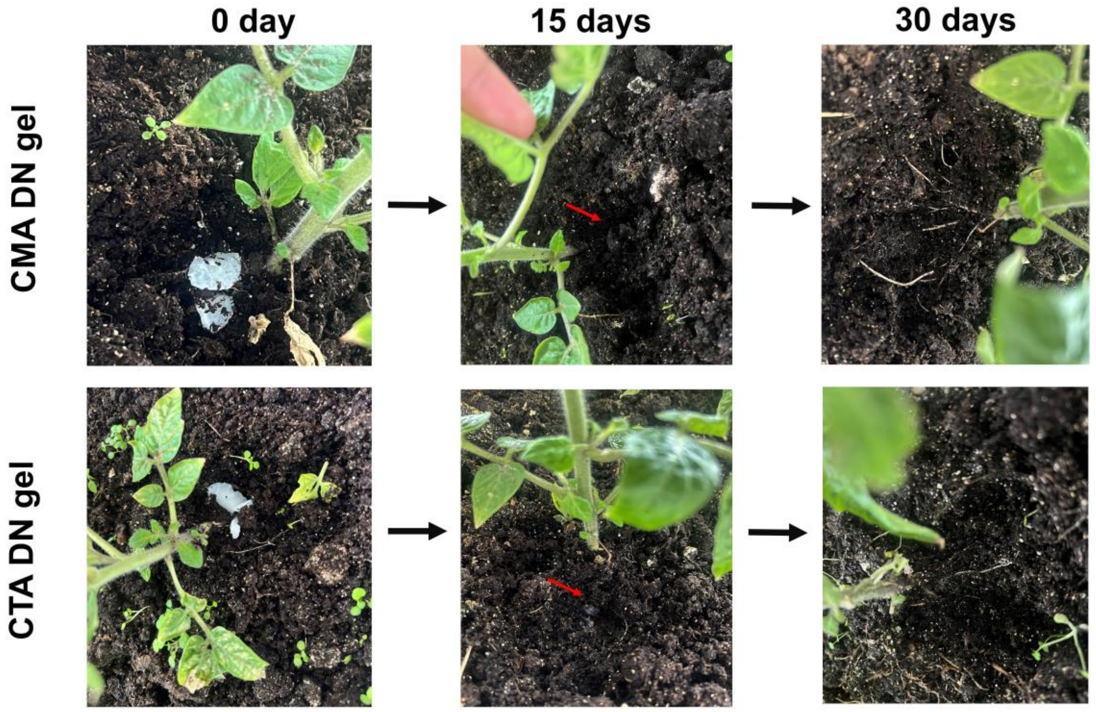

Supplementary Figure 39: Degradation process of CMA DN gel and CTA DN gel blocks in soil.

Comment 2. A brief comparative discussion with other pH-responsive systems (e.g., MOFs, nanoparticles) in the Introduction or Discussion could better contextualize the novelty of the current work.

Response 2: We thank the reviewer for this valuable suggestion. In response, we have revised the Introduction to incorporate a concise comparative discussion of representative pH-responsive systems, including MOF-based and mesoporous silica nanocarriers,

in agricultural applications. This addition has been integrated into the original narrative to improve the logical flow and to more clearly highlight the limitations of existing nanomaterial-based systems, particularly for soil-borne and root-associated disease control. The following sentences and related references have been added in the Introduction (line 52-60):

"*For example, Liang et al. developed a pH-responsive core-shell metal-organic framework (MOF) nanocarrier using ZnO and ZIF-8 via an in-situ growth strategy.*[20] *This system enabled pH-responsive release of berberine and significantly reduced the incidence and severity of tomato bacterial wilt compared to the aqueous formulation. In addition, other nanomaterials, such as mesoporous silica nanoparticles, have been also widely explored for the construction of pH-responsive pesticide delivery systems for plant disease control.*[22] *However, these systems are predominantly applied to foliar application,*[22] *and their potential for managing root-associated diseases has not been systematically investigated.*"

Comment 3. The authors note that CTA performs better due to more carboxyl groups and simpler synthesis. A side-by-side comparison table in the supplementary information summarizing the key differences (e.g., release rate, mechanical strength, cost) would be helpful.

Response 3: We thank the reviewer for this suggestion. In response, we have compiled a side-by-side comparison of the key parameters of CMA DN gel and CTA DN gel in the new **Supplementary Table 10**, which allows for a clearer and more direct evaluation between the two systems. As shown in the table, CTA DN gel exhibits superior performance in terms of mechanical properties as well as the release of Phe and Zn^{2+} compared to CMA DN gel.

To further address this point, we have also incorporated corresponding statements in the Discussion section to better highlight these differences and their underlying rationale (line 970-972):

"*However, CTA DN gel exhibited superior pH-responsive controlled-release performance, enhanced mechanical properties (***Supplementary *Table 10****), and a stronger ability to induce systemic resistance in plants.*"

Supplementary Table 10: Comparison of key properties of CMA DN gel and CTA DN gel.

Key indicators	CMA DN gel	CTA DN gel	P value[a]
Strength (G')	3748.9 Pa	**6865.4 Pa**	**0.004**
Pesticide release (pH=5)	74.8%	80.0%	0.19
Phe release (pH=5)	47.0%	**56.2%**	**0.026**
Zn^{2+} release (pH=5)	61.3%	**73.0%**	**0.02**

Yield (kg/plant)	3.1	3.5	0.29

[a]P value < 0.05 is considered statistically significant.

Comment 4. Figure 4a-d is not sufficiently clear.

Response 4: We thank the reviewer for pointing out this issue. We have reorganized Figure 4 accordingly. As the original Figure 4 contained too many panels, it was difficult to present the details of each result clearly. Therefore, we have moved the original Figures 4a and 4c to the Supplementary Information, where they are now presented as **Supplementary Figures 18** and **20**, respectively. The remaining panels (original **Figures 4b** and **4d**) have been rearranged together with other relevant results to form a new **Figure 4** (see below), with improved clarity and readability.

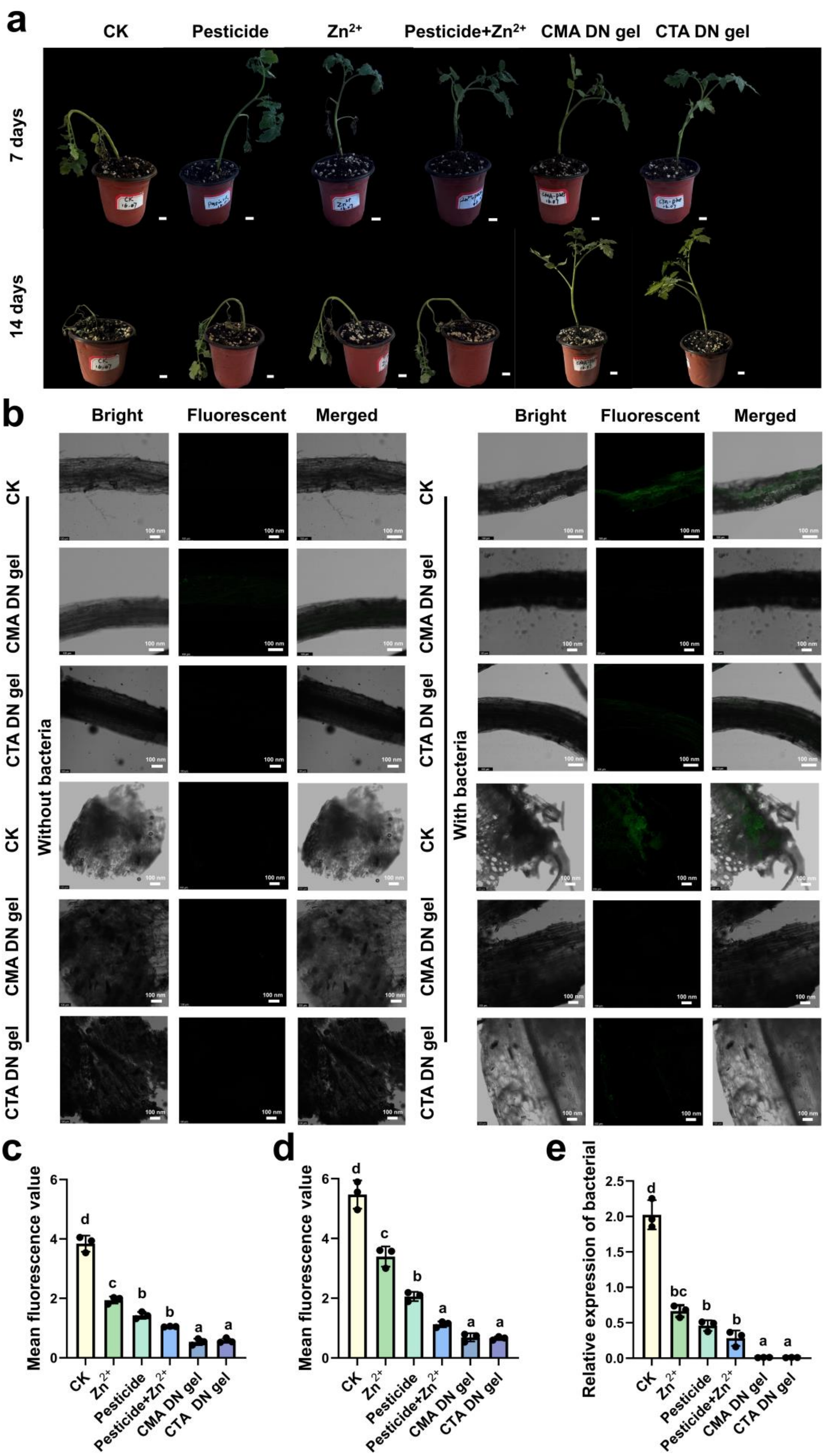

a
CK
Pesticide
Zn2+
Pesticide+Zn2+
CMA DN gel
CTA DN gel
7 days
14 days
b
Bright
Fluorescent
Merged
CK
CMA DN gel
CTA DN gel
Without bacteria
With bacteria
100 nm
c
d
e
Mean fluorescence value
Relative expression of bacterial
CK
Zn2+
Pesticide
Pesticide+Zn2+
CMA DN gel
CTA DN gel

Figure 4: *In vivo* assessment of the protective efficacy of CMA/CTA DN gels against *R. solanacearum* infection in tomato plants. **(a)** Representative photographs of tomato plants exhibiting symptoms of bacterial wilt after treatment with CMA/CTA DN gels (the white scale bars indicate 1 cm). **(b)** Cross-sectional and longitudinal confocal laser scanning microscopy images of tomato roots infected with *R. solanacearum*-GFP, showing bacterial distribution following the treatment with DN gels. **(c, d)** Quantitative analysis of fluorescence intensity in **(c)** cross-sectional and **(d)** longitudinal root images to assess *R. solanacearum*-GFP load after hydrogel treatment. **(e)** qPCR analysis of *R. solanacearum*-GFP abundance in tomato roots post-treatment with CMA and CTA DN gels. "CK" denotes control plants treated with deionized water. The mean values presented in each bar, accompanied by different letters in panels c, d and e ($P < 0.05$), indicate significant differences based on one-way ANOVA with post hoc test. Vertical bars indicate standard deviations.

Reviewer #2:

General comment: This manuscript describes a dual pH-responsive double-network (DN) hydrogel designed for the control of bacterial wilt caused by Ralstonia solanacearum. While the study integrates material design, antibacterial performance, and field testing, several key conceptual and mechanistic claims are not sufficiently supported by experimental evidence. In particular, the rationale for the material design, the definition of the "double-network" structure, the claimed targeting behavior, and the assessment of biosafety and ecological impact remain inadequate for the standards of Nature Communications. Substantial additional work would be required to address these concerns.

Response: We thank the reviewer for the constructive comments. In the revised manuscript, we have clarified the design rationale and provided additional evidence to better define the double-network structure and support the proposed mechanism. We have also strengthened the evaluation of biosafety and ecological impact through additional experiments, including pollen germination assays and soil metagenomic analysis.

Comment 1: The manuscript employs both CTA and CMA hydrogels in a double-network structure, yet fails to provide a convincing scientific rationale for why both are necessary. Their respective advantages, functional distinctions, and synergistic contributions are not clearly articulated.

Response 1: We thank the reviewer for this comment. We apologize for not clearly explaining the rationale for employing both CMA and CTA hydrogels in the double-network system. Carboxymethylated agarose (CMA) is a commonly used pH-responsive material, particularly in the biomedical field, and it was therefore initially selected as a representative system (Khan et al., 2019, 124: 1220-1229; Huang et al., 2020, 30, 2000644). However, during our study, we found that its synthesis was relatively complex and required substantial amounts of organic solvents, such as isopropanol and ethanol, during both the reaction and purification, which increases cost and raises environ-

mental concerns for agricultural use. Moreover, the relatively limited number of carboxyl groups in CMA may restrict its pH responsiveness. To address these limitations, we explored citric acid-modified agarose (CTA) as a sustainable alternative. CTA can be synthesized through a simple heating process under mild conditions without the use of organic solvents, and purification can be achieved using water alone, making it more environmentally friendly and cost-effective. In addition, CTA is expected to possess a higher density of carboxyl groups than CMA, which may enhance its pH responsiveness and coordination with Zn^{2+}, thereby improving mechanical strength and controlled-release performance, as supported by the data summarized in the new **Supplementary Table 10**. Furthermore, citric acid is a natural metabolic intermediate and metal-chelating agent in plants, which may facilitate nutrient uptake and contribute positively to plant growth after hydrogel degradation. These considerations, however, are primarily based on theoretical analysis and require experimental validation; therefore, both CMA and CTA were incorporated in this study to enable a direct comparative evaluation and to determine whether CTA, as a more sustainable alternative, offers advantages over the more established CMA system for agricultural applications. Both the synthetic strategy and experimental results indicate that CTA DN gel is more suitable for such use. To clarify this rationale, we have revised the final paragraph of the Introduction to explicitly describe the respective characteristics, differences, and motivations for using both CMA and CTA (line 84-107):

"*To impart pH responsiveness, carboxyl groups were incorporated in the agarose structure through controlled chemical modifications.*[33,34] *The first strategy involved the introduction of carboxymethyl groups onto agarose to obtain carboxymethylated agarose (CMA). CMA can coordinate with Zn^{2+} to form a pH-responsive hydrogel. Under acidic conditions, the protonation of the carboxyl groups weakens their binding to Zn^{2+}, leading to a loosening of the hydrogel structure, facilitating the release of both drugs and Zn^{2+}.*[33,35] *Owing to this mechanism, CMA-based hydrogels have been widely applied in drug delivery systems, particularly for anticancer and antibacterial agents.*[33] *However, the synthesis of CMA is relatively complex and typically requires substantial amounts of organic solvents,*[33] *which may increase production costs and raise potential environmental concerns for agricultural applications. In addition, the relatively low density of carboxyl groups in CMA may limit its pH responsiveness. To overcome these limitations, a second strategy was proposed by introducing citric acid, which contains multiple carboxyl groups, to modify agarose, yielding citrate agarose (CTA).*[34] *Citric acid can be covalently linked to agarose via esterification under relatively mild conditions,*[33] *without the need for organic solvents, making the process more environmentally friendly and cost-effective. More importantly, the presence of carboxylic acids in the structure of citric acid is expected to enhance the coordination of Zn^{2+}, thereby improving gel strength and pH responsiveness. Despite these potential advantages, studies on CTA hydrogels for drug delivery remain limited, and it is still unclear whether CTA offers superior overall performance compared to CMA. Therefore, a systematic comparison between CMA- and CTA-based hydrogels is necessary to evaluate their relative performance and to determine their suitability for agricultural applications.*"

Supplementary Table 10: Comparison of key properties of CMA DN gel and CTA DN gel.

Key indicators	CMA DN gel	CTA DN gel	P value[a]

Strength (G')	3748.9 Pa	**6865.4 Pa**	**0.004**
Pesticide release (pH=5)	74.8%	80.0%	0.19
Phe release (pH=5)	47.0%	**56.2%**	**0.026**
Zn^{2+} release (pH=5)	61.3%	**73.0%**	**0.02**
Yield (kg/plant)	3.1	3.5	0.29

[a]P value < 0.05 is considered statistically significant.

Comment 2: The choice of L-phenylalanine among numerous amino acids is not convincingly justified. The manuscript does not clearly explain its unique advantages in terms of self-assembly, bioactivity, or coordination chemistry compared with other amino acids.

Response 2: We thank the reviewer for these insightful comments. We agree that the selection of amino acids requires clear justification, particularly in comparison with other potential candidates. In agricultural applications, material selection must balance functionality with cost, synthetic simplicity, and environmental compatibility. These factors directly determine large-scale feasibility and ecological sustainability. In this context, L-phenylalanine (Phe) is a practical choice. It is an essential amino acid for plants, relatively inexpensive, and widely used as a nutrient additive in fertilizers (Mallhi et al., 2019, *Plants*, 8(11), 525). This indicates its inherent suitability for agricultural applications. Although many amino acids and self-assembly strategies are available, a number of reported approaches rely on solvent-triggered assembly, often involving organic solvents, such as dimethylsulfoxide (Singh et al., *Langmuir,* 2025, 41(3), 1639-1650 (2025); Singh et al., 2020, *Soft Matter*, 16(28), 6599-6607). Such processes may increase cost and introduce potential environmental risks. In contrast, Phe can dissolve in water and directly coordinate with Zn^{2+}. This enables the formation of supramolecular assemblies, such as nanofibers or hydrogels, in a fully aqueous system. This solvent-free process is simpler and more environmentally benign. Notably, many other amino acids cannot achieve similar self-assembly behavior under comparable conditions (Ma et al., 2023, *ChemistrySelect*, 8(18), e202301151). In addition, this coordination-driven assembly allows the formation of a secondary network without introducing extra components. Zn^{2+} simultaneously participates in constructing both the agarose-based network and the Phe-based network. This simplifies the system design. More importantly, under acidic conditions, the carboxylic group of Phe becomes protonated. This weakens its coordination with Zn^{2+} and leads to structural disruption. As a result, the pH responsiveness of the overall system is further enhanced when Phe-Zn^{2+} serves as the second component of the gel. Overall, considering its low cost, agricultural compatibility, aqueous self-assembly capability, and intrinsic pH-responsive behavior, the selection of Phe–Zn^{2+} as the secondary network is both rational and advantageous.

Regarding the structural issues related to the incorporation of Phe-Zn^{2+} into the CMA

and CTA hydrogels, please refer to our responses to Comments 3 and 4 below.
We have discussed the rationale for selecting Phe in the Introduction (line 112-131):
"*To overcome these limitations, we planned to introduce a second network into the CMA or CTA hydrogels. In this context, self-assembled amino acids were considered ideal building blocks, as they can function as an effective secondary network in DN systems. Their self-assembly is driven by non-covalent interactions, such as metal coordination, H-bonding and π-π stacking, leading to the formation of a dynamic, reversible, and stimuli-responsive structure. Among various amino acids and assembly strategies, L-phenylalanine (Phe), an essential amino acid for plant growth,*[36] *was selected. Specifically, Zn^{2+} was then employed to coordinate with Phe, enabling the in-situ formation of a Phe-Zn^{2+} supramolecular secondary network.*[37] *Notably, this assembly process occurs directly in aqueous conditions, avoiding the use of organic solvents (e.g., dimethylsulfoxide),*[38, 39] *which are commonly employed in solvent-mediated strategies to induce or regulate the self-assembly of amino acids or their derivatives, thereby ensuring improved environmental compatibility and safety. In addition, this strategy minimizes complexity of the system, as the introduction of Zn^{2+} is sufficient to promote the formation of both the CMA/CTA network and the Phe-Zn^{2+} secondary network. More importantly, under acidic conditions, the protonation of the carboxylic group of Phe weakens its coordination with Zn^{2+}, leading to the disassembly of the Phe-Zn^{2+} network. This feature further enhances the pH responsiveness of the CMA and CTA hydrogels. Based on these advantages, in this study the combination of the couple Phe-Zn^{2+} was ultimately selected for the formation of the secondary network.*"

Comment 2. More importantly, although Phe–Zn^{2+} is claimed to form a secondary network, there is no direct experimental evidence or characterization demonstrating its interaction or crosslinking with the CMA/CTA hydrogel system.
Comment 3. The scale bars in Figure 2b-h are unclear. Furthermore, the drastic morphological changes observed after introducing the Phe-Zn^{2+} system are not adequately explained. The origin, composition, and formation mechanism of the observed surface film-like structure remain speculative and unsupported by experimental analysis.
Comment 4. Although the hydrogel is repeatedly described as a 'double-network' system, the manuscript does not provide convincing evidence to support this claim. There is a lack of structural, mechanical, or network-specific characterization demonstrating the coexistence of two independent yet interpenetrating networks. Without such evidence, the DN designation appears overstated.

Response 2, 3 & 4: We thank the reviewer for raising these critical points. To address Comments 3 and 4, related to the structural characteristics and network organization of the CMA DN and CTA DN hydrogels, we provide a unified response to ensure clarity and logic consistency.

In principle, DN hydrogels are currently categorized into three types: fully chemically crosslinked DN hydrogels, fully physically crosslinked DN hydrogels, and hybrid DN hydrogels featuring both chemical and physical crosslinking (Yang et al., *Adv. Funct. Mater*. 2022, 32 (19), 2110244.). Among them, fully physically crosslinked DN hydrogels are generally composed of naturally derived biopolymers such as alginate, agarose, gelatin, and chitosan, which confer excellent biosafety (Zhao et al., *Pharma-*

ceutics. 2023, 15 (10), 2514.). Both networks are crosslinked through non-covalent interactions (e.g., hydrogen bonding, hydrophobic or electrostatic interactions), endowing the hydrogels with advantages such as reversibility and stimuli-responsiveness (Zhao et al., *Pharmaceutics*. 2023, 15 (10), 2514.). These features make them ideal candidates for biomedical applications in drug delivery (Zhao et al., *Pharmaceutics*. 2023, 15 (10), 2514.). Following this design principle, we selected agarose as the primary network because of its well-established gelation behavior and suitability for environmentally benign applications. However, agarose-based hydrogels alone generally suffer from limited mechanical strength. To overcome this limitation, we introduced a secondary physically crosslinked network based on Phe-Zn^{2+} supramolecular assembly, a strategy that is characteristic of DN hydrogels and is known to significantly enhance mechanical performance (Chen et al., *J Mater Chem B*. 2015,3, 3654-3676). This effect is clearly supported by our rheological results: the storage modulus (G′) of CMA-Zn^{2+} and CTA-Zn^{2+} hydrogels was measured to be 1576 Pa (**Figure 1f**) and 3076 Pa (**Figure 1g**), respectively, whereas the incorporation of the Phe-Zn^{2+} network increased these values to 3942 Pa and 6859 Pa (**Figure 1h**). These results demonstrate that the Phe–Zn^{2+} network effectively reinforces the agarose-based network, confirming that our hydrogels exhibit the key compositional and functional characteristics of DN systems.

From a structural perspective, the DN hydrogels are typically characterized by the coexistence of two interpenetrating or intertwined networks (Chen et al., *J Mater Chem B*. 2015,3, 3654-3676). In our initial SEM observations, structures resembling the Phe–Zn^{2+} assemblies were identified in the DN hydrogels (**Figure 2e, f**); however, the spatial relationship between these structures and the agarose network could not be clearly resolved. To address this limitation, we performed additional cryo-SEM characterization to better preserve and visualize the native hydrogel architecture. As shown in **Figure 1e**, the CMA and CTA hydrogels display the characteristic honeycomb-like porous morphology of agarose-based networks (Rahbani, J. et al., 2013, 34(3), 405-408.), whereas the Phe-Zn^{2+} system exhibits a distinct rod-like structure. Importantly, after incorporation of Phe-Zn^{2+} into the CMA or CTA hydrogels, similar rod-like structures are clearly observed to be embedded within and distributed throughout the agarose network in both DN systems. This observation provides direct morphological evidence of an interpenetrating and intertwined network architecture. Taken together, the combined evidence from material composition, mechanical reinforcement, and structural characterization supports the conclusion that the CMA/CTA and Phe-Zn^{2+} components form a DN hydrogel system.

We have revised the manuscript accordingly to include the cryo-SEM results and an expanded discussion to more clearly substantiate the DN structure and address the reviewer’s concerns:

Line 194-205

"*To further elucidate the internal architecture of these hydrogels, cryogenic scanning electron microscopy (cryo-SEM) characterization was conducted. As shown in **Figure 1e**, both CMA and CTA hydrogels exhibit a characteristic honeycomb-like porous structure, consistent with previous reports.[45] The CTA hydrogel exhibits a slightly smaller pore size than the CMA hydrogel, which may lead to differences in their mechanical properties. In contrast, the Phe-Zn^{2+} hydrogel displays a distinct rod-like morphology. Importantly, in both CMA DN and CTA DN hydrogels, rod-like features characteristic of the Phe-Zn^{2+} assemblies are uniformly distributed and embedded within the continuous agarose matrix, indicating the formation of a continuous and interpenetrating network architecture. This observation provides direct morphological evidence supporting the successful construction of a double-network system.*"

Line 213-217:

"*In addition, the CTA-Zn^{2+} gel exhibited markedly higher mechanical strength than the CMA-Zn^{2+} gel, which may be attributed to the higher density of carboxylic groups in CTA, leading to stronger coordination with Zn^{2+}, as well as to its smaller pore size (**Figure 1e**).*[46]"

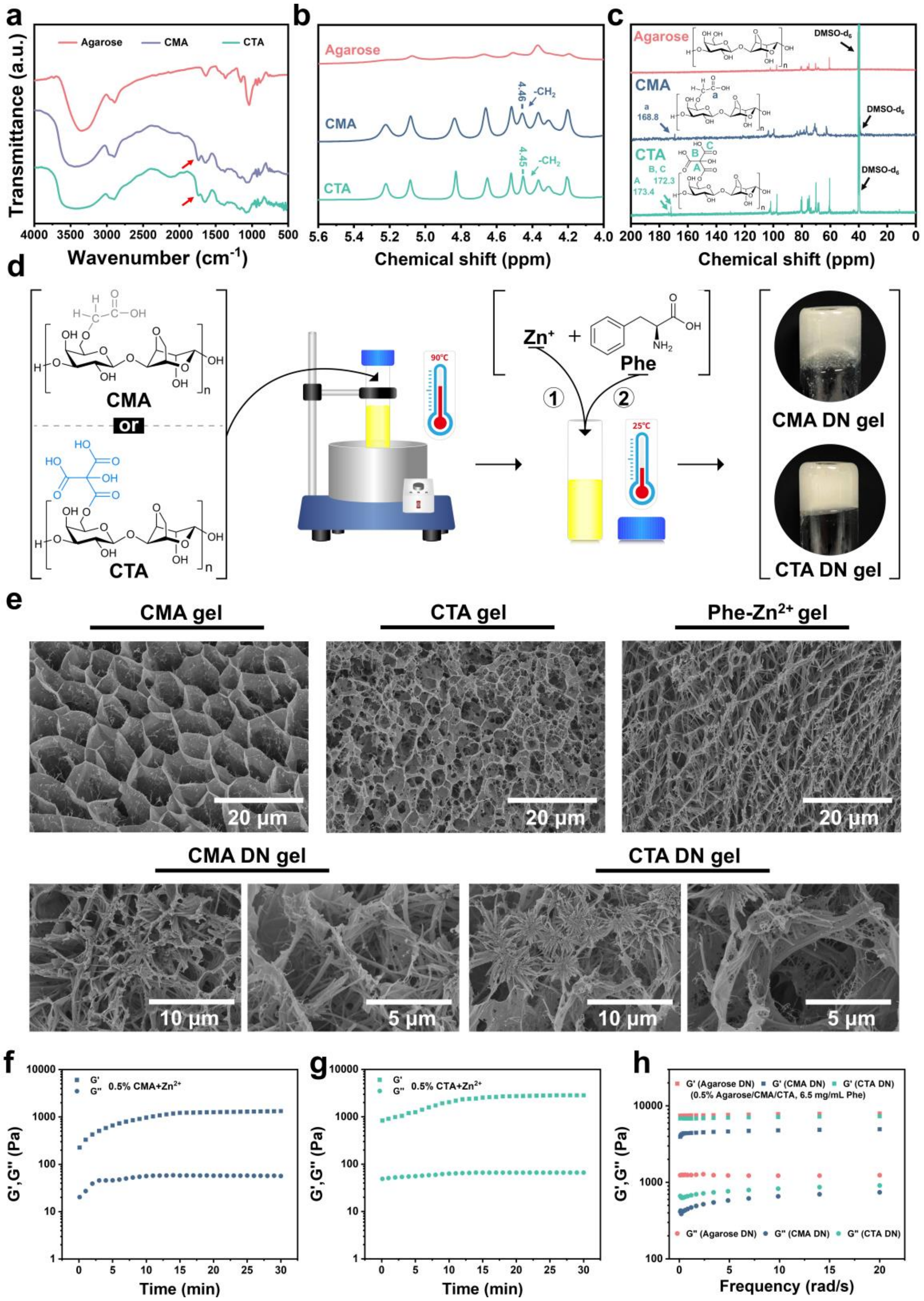

Figure 1. Physicochemical characterization of CMA/CTA and the corresponding DN gels. **(a)** FTIR spectra of agarose, CMA, and CTA. **(b)** ^{1}H NMR and **(c)** ^{13}C NMR spectra of agarose in

DMSO-d_6 (the assignment of carbon atoms with numbers in the agarose structure also applies to the structures of CMA and CTA). **(d)** Schematic illustration of the preparation procedure for CMA or CTA DN gels. **(e)** Cryo-SEM images of CMA, CTA, Phe-Zn^{2+}, CMA DN, and CTA DN hydrogels. **(f, g)** Rheological monitoring of gelation kinetics via time sweep analysis of storage modulus (G') and loss modulus (G") for **(f)** 0.5% CMA + Zn^{2+} and **(g)** 0.5% CTA + Zn^{2+}. **(h)** Frequency sweep profiles of agarose-, CMA-, and CTA-based DN gels.

Comment 5: The antibacterial activity of the CMA/CTA DN hydrogel appears to be largely non-selective, driven primarily by the release of Zn^{2+} and a broad-spectrum pesticide. The manuscript does not address the potential negative impacts on beneficial soil microbiota during field application. Moreover, the risks of long-term use, including bacterial resistance development and environmental contamination, are not meaningfully discussed or experimentally evaluated.

Response 5: We highly acknowledge these concerns and we have carefully addressed them through additional experiments. Specifically, we performed metagenomic sequencing on soil samples collected from the field trials in which CMA/CTA DN gels were applied in June 2025. The interval between the initial application of the hydrogels and sample collection was approximately seven months. Following the completion of the field experiments, no additional crops were planted, and no agricultural practices were conducted, thereby minimizing external disturbances to the soil microbial community. Under these controlled conditions, we evaluated whether the application of CMA/CTA DN gels influenced the soil microbial community composition and functional potential. The results are summarized as follows:

1. Microbial Community Composition

Beta-diversity analyses based on Bray-Curtis distance revealed significant separation between CK (control plants treated with water) and CMA/CTA-treated soils (Analysis of Similarities (ANOSIM) R = 0.667, P = 0.003; Non-metric Multidimensional Scaling (NMDS) stress = 0.038) (**Figure 7 a** and **7 b**). These results indicate that the hydrogel treatment reshaped the microbial community structure.

Importantly, however:

- No drastic reduction in sequencing depth, gene richness, or assembly quality was observed (**Supplementary Table 7** and **Supplementary Table 8**).
- Dominant phyla remained present across all treatments.
- No evidence of uncontrolled bloom or collapse of major microbial taxa was detected.

Therefore, the observed shift represents community restructuring rather than ecological disruption.

2. Functional Stability of Core Metabolism

Kyoto Encyclopedia of Genes and Genomes (KEGG) functional profiling showed that the relative abundance of core metabolic pathways remained highly stable among the treatments. For example:

- "Metabolic pathways" remained approximately 17.4-17.6% across the groups (**Figure 7c, Supplementary Data 1**).
- "Biosynthesis of secondary metabolites" remained approximately 7.4%.

Although several pathways (e.g., two-component systems, carbon metabolism) showed raw $P < 0.05$, none remained significant after False Discovery Rate (FDR) correction ($q \approx 0.089$) (**Figure 7c, Supplementary Data 1**), indicating no robust functional collapse or large-scale metabolic reprogramming. Thus, the structural shifts did not

translate into a loss of essential ecosystem metabolic functions.

3. Antibiotic Resistance Gene (ARG) Assessment

Comprehensive Antibiotic Resistance Database (CARD) annotation identified ARG-related genes across all samples (84,250 annotated unigenes in total), primarily belonging to efflux pump families (e.g., patA, emrB), which are commonly present in environmental microbiomes (**Figure 7d, Supplementary Data 2**).

Linear discriminant analysis Effect Size (LEfSe) analysis identified two resistance-related markers enriched in the CMA group (Erm(31) and AxyY, P = 0.023) (**Supplementary Table 9**). However:

- Total ARG abundance, calculated from CARD annotations, was lower in CMA- and CTA-treated soils compared to the control group. This indicates that the hydrogel application did not promote ARG enrichment at the community level (**Figure 7e**).
- No significant amplification of β-lactam, glycopeptide, or multidrug resistance modules at the community level was detected.
- ARG expansion was not accompanied by increased mobile genetic element signals (see below).

Therefore, while limited marker-level enrichment was detected, there was no evidence of systematic or large-scale resistance proliferation under the tested conditions.

4. Mobile Genetic Elements (MGE)

Annotations of transposase, integrase, and recombinase genes were observed across all groups (**Figure 7f**). However:

- No statistical evidence indicated treatment-induced expansion of MGE abundance.
- No coordinated ARG-MGE co-enrichment pattern was observed.

This suggests that the hydrogel application did not measurably enhance a horizontal gene transfer potential.

5. Long-Term Ecological Risk Consideration

We acknowledge that repeated or long-term application may impose selective pressure on soil microbiota. Accordingly, we have expanded the Discussion section to:

- Clarify that current conclusions are based on short-term field evaluation.
- Propose longitudinal monitoring of ARGs and MGEs under repeated application.
- Compare the ecological modulation observed here with the known resistance risks associated with conventional chemical bactericides.

Overall, although CMA and CTA significantly reshaped microbial community composition, the preservation of core metabolic functions, absence of large-scale ARG amplification, and lack of MGE expansion indicate that these hydrogels do not induce detectable ecological destabilization or resistance escalation under the tested field conditions.

To address these concerns, we have also provided a detailed discussion of the soil metagenomic sequencing results in the Results section of the revised manuscript, adding the new Figure 7 (line 697-761):

"*To systematically evaluate the long-term ecological impact of CMA/CTA DN gels under field conditions, we performed a metagenomic sequencing on soil samples collected approximately seven months after hydrogel applications. During this period, no additional crops were planted and no agricultural practices were conducted, minimizing external disturbances and allowing a more direct assessment of microbial responses. Beta-diversity analysis based on Bray-Curtis distance revealed a clear and statistically significant separation between CK and CMA/CTA-treated soils (Analysis of Similarities (ANOSIM) R = 0.667, P = 0.003; Non-metric Multidimensional Scaling (NMDS) stress*

= 0.038; ***Figure 7a*** *and* ***7b****), suggesting a treatment-driven shift in the community composition. Despite this separation, sequencing depth, gene richness, and assembly quality remained comparable across all groups (***Supplementary *****Table*** *7 and* **Supplementary** ***Table 8****). The dominant phyla were consistently preserved, and the overall taxonomic profile showed no signs of imbalance. Together, these patterns point to community restructuring rather than ecological disruption. A similar trend was observed at the functional level. Kyoto Encyclopedia of Genes and Genomes (KEGG) profiling showed that core metabolic pathways remained highly stable among the treatments, with "metabolic pathways" (~17.4–17.6%) and "biosynthesis of secondary metabolites" (~7.4%) exhibiting minimal variation (****Figure 7c, Supplementary Data 1****). Although several pathways reached nominal significance ($P < 0.05$), these differences were not retained after False Discovery Rate (FDR) correction ($q \approx 0.089$)(* ***Figure 7c, Supplementary Data 1****), suggesting that the observed variations were not deleterious/dramatic/influent. Thus, this composition shift did not translate into functional impairment of key metabolic processes. Comprehensive antibiotic resistance database (CARD) annotation identified antibiotic resistance genes (ARG)-related genes across all samples (84,250 annotated unigenes), predominantly belonging to commonly occurring efflux pump families (e.g., patA and emrB;* ***Figure 7d, Supplementary Data 2 (ARO abundance)****). Linear discriminant analysis Effect Size (LEfSe) analysis further detected limited enrichment of two markers (Erm(31) and AxyY, $P = 0.023$) in the CMA group (***Supplementary** ***Table 9****). At the community level, however, total ARG abundance was lower in CMA- and CTA-treated soils than in the control (****Figure 7e****), and major resistance categories, including β-lactams, glycopeptides, and multidrug resistance, did not show coordinated enrichment. This pattern was further supported by the analysis of mobile genetic elements (MGEs) (****Figure 7f****). Transposase, integrase, and recombinase genes were detected across all groups, however their abundance remained statistically comparable. In addition, no coupled increase between ARGs and MGEs was observed, suggesting limited potential for enhanced horizontal gene transfer under these conditions. Taken together, CMA and CTA DN gels induced measurable shifts in microbial community composition without disrupting core ecosystem functions. The absence of coordinated ARG expansion and MGE enrichment further suggests that the system does not promote resistance proliferation under the tested conditions. This favorable profile is likely related to the intrinsic properties of the materials. Agarose and Phe are natural polymers and amino acids, respectively, and are generally considered biocompatible and environmentally benign. Their use therefore minimizes potential ecological risks in agricultural systems. The loaded antibiotic, zhongshengmycin, is a microbial secondary metabolite produced by Streptomyces violaceusniger and has been widely applied in China for the control of bacterial diseases such as bacterial wilt.[67] It has a low propensity to induce environmental resistance or ecological harm.[68] To date, there is no clear evidence linking its application to soil microbial imbalance or major ecological disturbance. In contrast, conventional antibiotics used in agriculture, including aminoglycosides, tetracyclines, and quinolones, have been widely reported to disrupt soil microbial communities.[69] These compounds can enrich target-specific ARGs and promote co-selection of resistance determinants, thereby accelerating the spread of multidrug resistance.[69] For example, oxytetracycline can increase the abundance of tetracycline resistance genes (e.g., tetA, tetL, tetM, tetQ, and tetW) by 10- to 1000-fold even at low exposure levels (2-70 μg/kg), highlighting its strong selective pressure on the soil resistome.[70] Such effects were not observed in the present study following the application of zhongshengmycin-loaded CMA and CTA DN gels. Zn^{2+} is an essential micronutrient for plant growth and immunity,[55] but excessive application may lead to phytotoxicity or*

environmental risks. In this study, no ecological destabilization was observed after seven months. This is likely due to the controlled Zn^{2+} dosage and the pH-responsive release behavior of the DN hydrogels, which helps preventing excessive zinc accumulation. It should be noted, however, that the current assessment is based on a single application. Longer-term monitoring and repeated application studies of CMA and CTA DN gels will be necessary to fully evaluate potential cumulative effects."

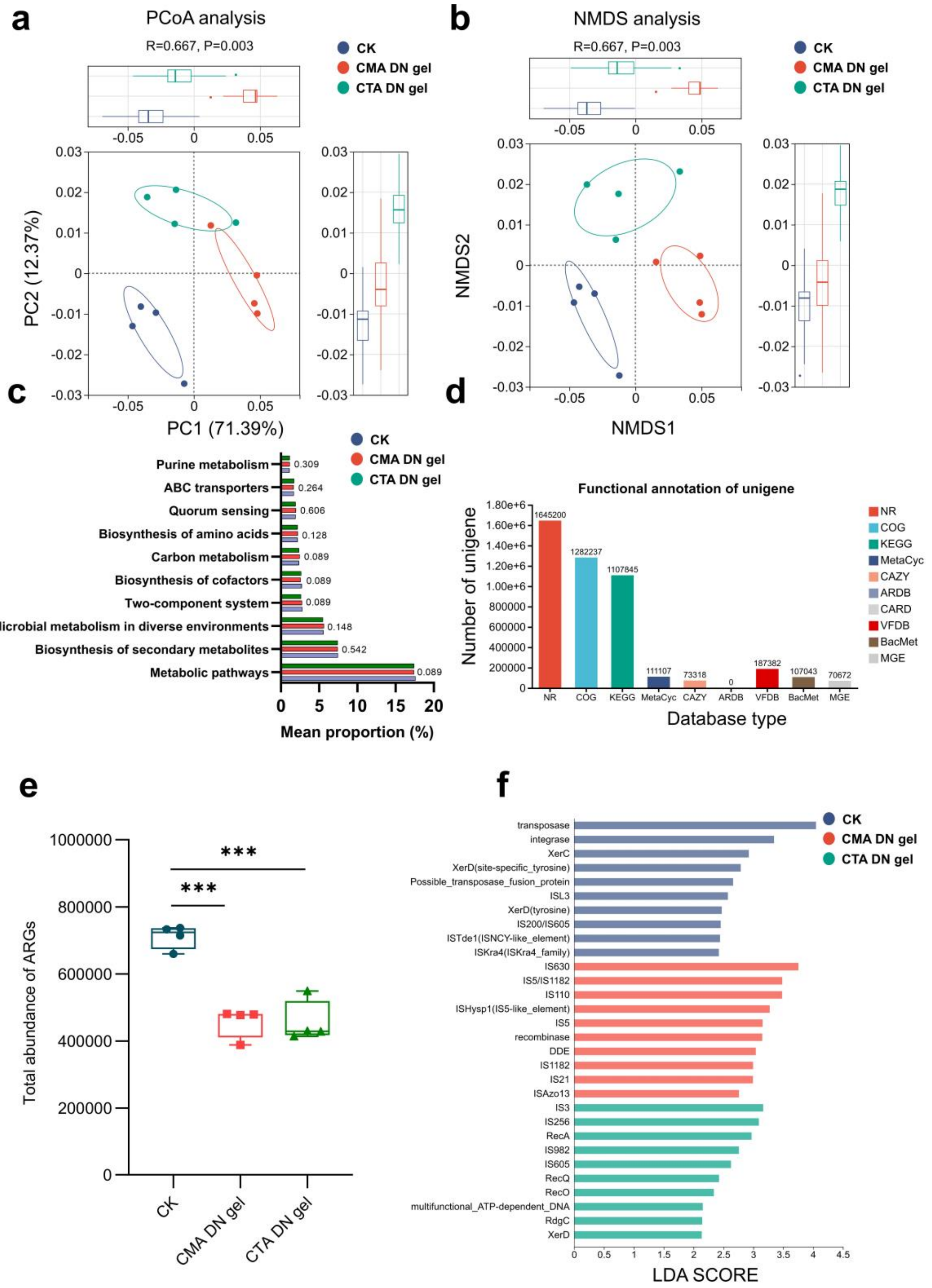

Figure 7: Metagenomic analysis of soil samples collected 7 months after the treatment with

CMA and CTA DN gels. **(a)** Principal coordinates analysis (PCoA). **(b)** Non-metric multidimensional scaling (NMDS). **(c)** Statistical significance testing based on KEGG profiling. **(d)** Summary of CARD annotation. **(e)** Comparison of total ARG abundance. **(f)** LEfSe analysis (LDA stands for Linear Discriminant Analysis). The asterisk indicates a significant difference according to one-way ANOVA with post hoc test (***P < 0.001). The vertical bars indicate standard deviations.

Comment 6: The manuscript claims that the hydrogel is "targeted" toward R. solanacearum, yet no specific targeting mechanism is demonstrated. The system appears to rely solely on environmental pH responsiveness, which is a general stimulus rather than a true biological targeting strategy. The absence of defined molecular, cellular, or microenvironmental targets undermines the validity of the targeting claim.

Response 6: We appreciate this comment. We would like to clarify that in the original manuscript, the term "targeted" was used because the pH-responsive drug release behavior of the CMA/CTA DN gels coincides with the pH conditions under which *R. solanacearum* infection typically occurs in the field. However, we did not incorporate a specific targeting mechanism, as our DN gels were not specifically engineered to target the microenvironment of bacterial wilt. Therefore, to ensure greater accuracy and scientific rigor, we have removed the term "targeted" throughout the manuscript (including the title) and replaced it with "efficient":
New title: *"Dual pH-Responsive Multifunctional Double Network Hydrogels for Efficient and Sustainable Control of Tomato Bacterial Wilt*"

Comment 7: The manuscript does not clearly state how many days after treatment the observations in Figure 4c were conducted. This lack of essential experimental detail compromises data reproducibility and interpretation.

Response 7: We apologize for overlooking this detail. Confocal observation of *R. solanacearum*-GFP was conducted after 24 h of culture. We have added this description in the Methods section of the revised manuscript (line 1081-1082):
"*Additionally, for R. solanacearum-GFP treated with the DN gels, confocal microscopy (Leica, stellaris5) was performed 24 h after treatment to observe fluorescence signals (excitation at 488 nm, emission at 498-570 nm) to assess bacterial proliferation.*".

Comment 8: In Figure 5c, seedling length under acidic conditions (pH = 5) does not appear to be clearly superior to that under neutral conditions (pH = 7). This weak or ambiguous difference does not convincingly support the claimed pH-responsive growth enhancement.

Response 8: We thank the reviewer for this comment. We apologize for not photographing the seedlings at the same level; the seedlings in the image show noticeable differences in height. In addition, the seemingly subtle difference by eye is mainly due to the irregularity of the seedlings. In fact, we had previously provided statistical charts of seedling lengths in the Supporting Information (**Supplementary Figure 24e** and **24f**). During the measurement, one person straightened the curved seedlings while another

measured and recorded their lengths. The results showed that seedlings grown under pH 5 were significantly longer than those grown under pH 7. To make the images more intuitive, we repeated the experiment and photographed the seedlings after straightening them.

For better organization and layout, we have moved the rice germination results originally presented in **Figure 5** (**Figure 5b** and **5c**) to the Supporting Information (**Supplementary Figure 24**). In addition, the results of the pollen germination and fruit set rate experiments were included in the revised **Figure 5** (as **Figure 5b** and **5c**, respectively)

Comment 9: The biosafety assessment is incomplete. Key agricultural indicators such as pollen germination rate and fruit set are not evaluated. Without assessing reproductive parameters, the manuscript does not sufficiently demonstrate that the hydrogel is safe for long-term agricultural use.

Response: We thank the reviewer for this valuable suggestion. We treated 4-week-old tomato plants with CMA/CTA DN gels. After flowering, three flowers were collected from each plant for pollen sampling, and pollen germination was assessed using a pollen germination kit (Beijing Solarbio Science & Technology Co., Ltd.). As shown in **Figure R1**, all treatment groups exhibited good pollen viability, with germination rates exceeding 80% and no significant difference compared to the control group. In addition, we evaluated the fruit set rate of the first inflorescence in tomato plants, and all groups showed fruit set rates above 70%. These results indicate that CMA/CTA does not adversely affect the reproductive system of tomato plants.

We have incorporated the results of pollen germination and fruit set into the revised manuscript (line 530-537):

"*Specifically, we evaluated the pollen germination rates and the fruit set to determine whether CMA/CTA DN gels affect the reproductive process of tomato plants. The results showed that all treated groups exhibited high pollen viability, with germination rates exceeding 80% (**Figure 5b**), while the fruit set rate of the first truss in all groups was above 70% (**Figure 5c**), with no significant difference compared to the control. These findings indicate that CMA/CTA DN gels do not adversely affect the reproductive performance of tomato plants.*"

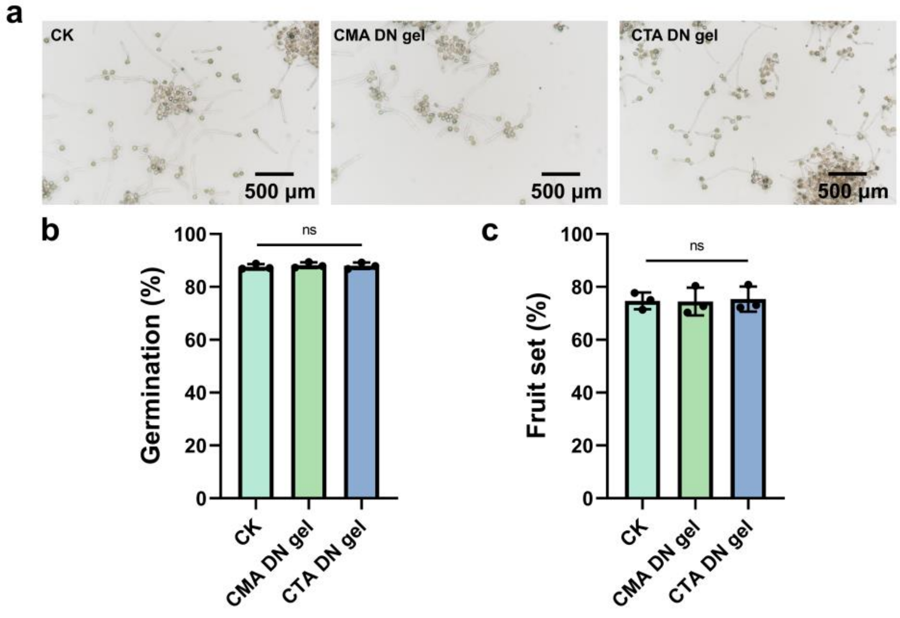

Figure R1: (a) Microscopic images of pollen germination in each treatment group following

the treatment of tomato plants with CMA and CTA DN gels under acidic conditions (pH 5). **(b)** Statistical analysis of pollen germination rates in each treated group following the treatment of tomato plants with CMA and CTA DN gels under acidic conditions (pH 5). **(c)** Statistical analysis of the fruit set rate of the first inflorescence in each treated group following the treatment of tomato plants with CMA and CTA DN gels under acidic conditions (pH 5). The vertical bars indicate standard deviations.

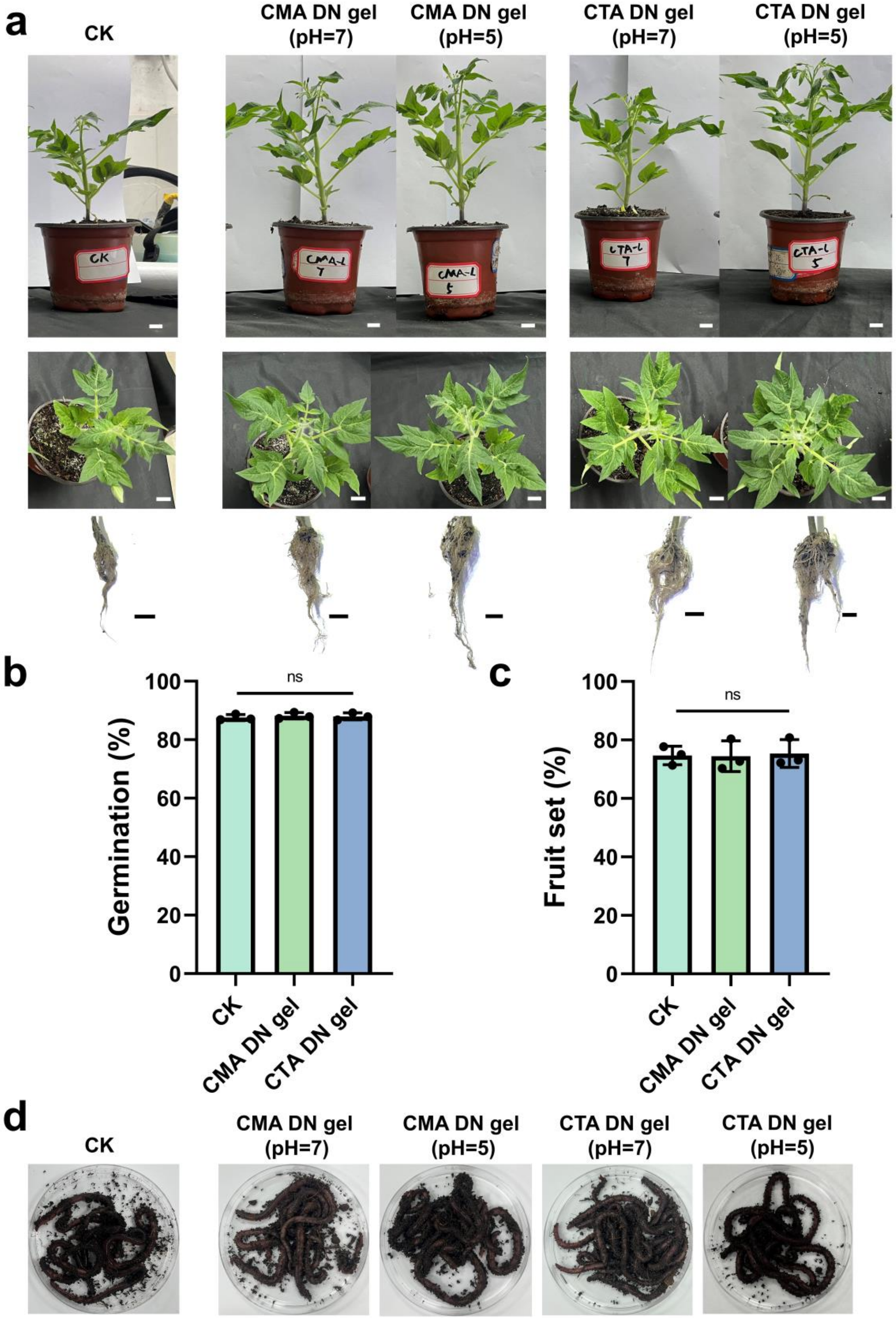

Figure 5. Biosafety evaluation of CMA and CTA DN gels. **(a)** Morphological assessment of tomato plants, including plant height, width, and root length, after a 3-week exposure to the solutions released from CMA and CTA DN gels under acidic (pH 5) and neutral (pH 7) conditions. **(b)** Statistical analysis of pollen germination rates in each treatment group following treatment of tomato plants with CMA and CTA DN gels under acidic conditions (pH 5). **(c)** Statistical analysis of the fruit set rate of the first inflorescence in each treated group following the treatment of tomato plants with CMA and CTA DN gels under acidic conditions (pH 5). **(d)** Representative images of earthworms after 7 days of exposure to soils adjusted to different pH levels (pH 5 and 7) and supplemented with CMA or CTA DN gels. Prior to testing, all released solutions were adjusted to pH 7 to ensure comparability. The white and black scale bars in panel a indicate 1 cm. The vertical bars in panel b and c indicate standard deviations.

Comment 10: All tomato plant images presented in the manuscript lack scale bars, preventing quantitative or comparative evaluation of plant growth and morphology.

Response 10: We thank the reviewer for this comment. We have added scale bars to all tomato plant images. Please refer to **Figure 4** and **Figure 5** for details.

Comment 11: The release profiles in Figure 6d indicate that free pesticide exhibits rapid and substantial release within the first few days, exceeding that of CMA DN and CTA DN. This raises the question of whether the hydrogel system provides a meaningful advantage over conventional pesticide application. Furthermore, the relatively low initial release (<20%) from CMA DN and CTA DN casts doubt on whether sufficient antibacterial concentrations are achieved.

Response 11: This is indeed an important point for discussion. First, it should be emphasized that the primary purpose of this experiment was to demonstrate that the hydrogel system provides prolonged pesticide activity in soil compared with the free pesticide solution. In field conditions, the environment is highly complex. For example, after applying a free pesticide solution to plant roots, rainfall can dilute most of the pesticide, and leaching may result in rapid pesticide loss through the soil, ultimately preventing an effective bacterial control and significantly reducing the disease management efficiency. Under such circumstances, repeated pesticide applications are often required, which increases environmental burden and production costs. Figure 6d confirms this scenario: following two simulated rainfall events (on day 2 and day 4), over 80% of the free pesticide had leached from the soil, leaving minimal residual pesticide. In contrast, CMA DN and CTA DN gels can sustain pesticide release under acidic soil conditions without rapid leaching, and even after multiple simulated rainfall events, a substantial fraction of the pesticide remains in the soil. This ensures prolonged bioavailability, enhancing the duration of antibacterial and disease-control effects while reducing the need for repeated applications. Moreover, although only approximately 20% of the pesticide is initially released in the first few days, the overall pesticide content encapsulated in the hydrogels is sufficiently high; once released and diluted by soil water or irrigation, it can achieve effective antibacterial concentrations. Our pot and

field experiments further confirmed the efficacy of CMA DN and CTA DN gels in disease control, with performance clearly superior to that of free pesticide solutions, which is attributable to their sustained-release characteristics.

Additionally, the pesticide concentration within CMA DN and CTA DN gels can be adjusted according to field requirements to ensure effectiveness. Most of the pesticide is protected within the hydrogels, and its sustained-release mechanism also prevents potential phytotoxicity or adverse effects on beneficial soil microorganisms that may occur with high-concentration pesticide applications. We already added some discussion in the MS to further highlight this point (line 666-678):

"*When combined with the drug release profiles (**Figure 2i-n**), these data clearly support the hypothesis that the CMA/CTA DN gels enable pH-triggered, sustained pesticide release in acidic soil conditions. The persistent acidic soil environment and the inherent sustained-release properties of the gels allow a prolonged pesticide release, resulting in efficient and continuous control of R. solanacearum in the soil, ultimately suppressing bacterial wilt effectively, as illustrated in **Figure 6e**. Simultaneously, soil-based degradation experiments further confirmed these observations (**Supplementary Figure 39**), demonstrating that CMA DN gels and CTA DN gels remain observable by naked eye in soil for approximately one month. A clear, gradual degradation of the hydrogels was monitored, and this process facilitates the sustained release of the encapsulated pesticide. Moreover, the slow degradation also guarantees prolonged retention of the active agent at the site of disease occurrence, supporting extended bioactivity.*"

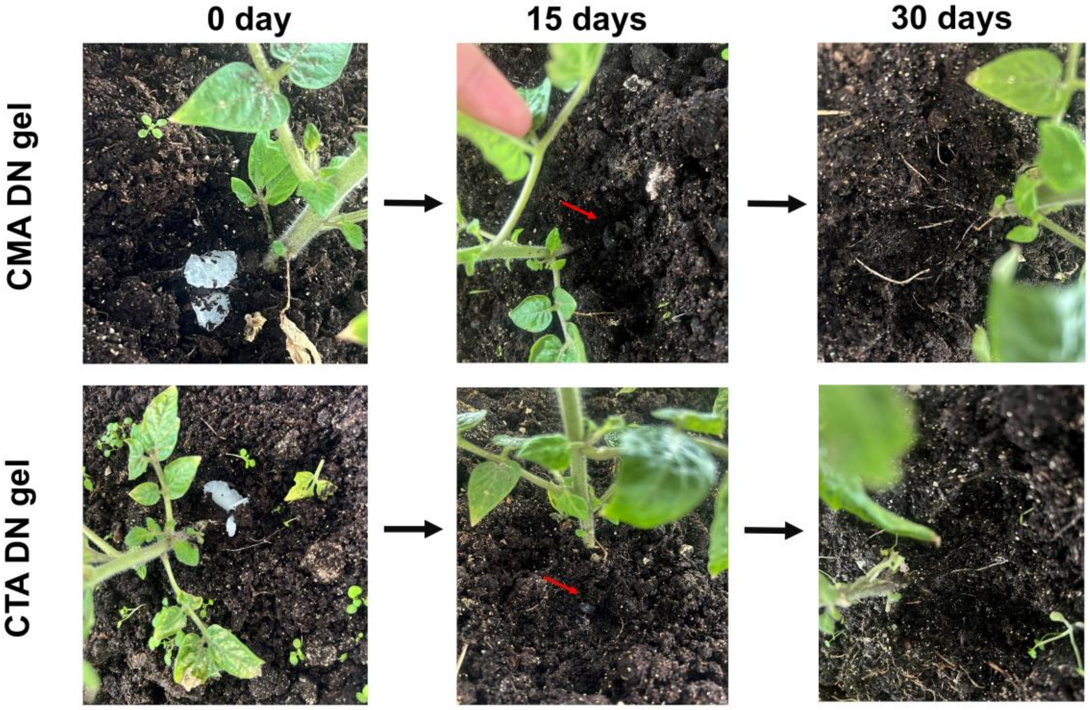

Supplementary Figure 39: Degradation process of CMA DN gel and CTA DN gel blocks in soil.

Comment 12: The transcriptomic data lack experimental validation, such as real-time PCR. Additionally, the analysis is superficial, focusing only on pathway enrichment without clearly distinguishing between upregulated and downregulated

genes. This significantly limits the mechanistic insight into plant immune responses and weakens the biological conclusions.

Response 12: We thank the reviewer for this valuable suggestion. We have strengthened the transcriptomic analysis and provided additional experimental validation in the revised manuscript.

First, to verify the reliability of the transcriptomic data, we performed quantitative real-time PCR (qPCR) analysis on a subset of differentially expressed genes. Specifically, four prominently upregulated genes and four markedly downregulated genes identified from the heatmap were selected for validation. The qPCR results showed a high level of consistency with the transcriptomic data, confirming the robustness and reliability of the sequencing results. Second, we have expanded the biological interpretation of the transcriptomic data by explicitly distinguishing between upregulated and downregulated genes. In particular, we further discussed key pathways and genes associated with plant immune responses, with an emphasis on ethylene-related signaling pathways. These analyses provide stronger mechanistic support for the role of CMA/CTA DN gels in enhancing tomato resistance.

The corresponding results and discussion have been incorporated into the revised manuscript (line 836-875):

"*To validate the reliability of the RNA-seq data, eight representative differentially expressed genes were selected for qPCR analysis (***Supplementary** ***Figure 40****). Among them, four genes, calcium-binding protein, CBP3 (Solyc06g068960.1), ethylene receptor, ETR4 (Solyc06g053710.3), ethylene-responsive transcription factor, ERF1 (Solyc05g051200.1), and endochitinase 3-like (Solyc10g055820.2), were consistently upregulated (***Supplementary** ***Figure 40a****), whereas four genes, calcium-dependent protein kinase, CDPK17-like (Solyc12g099790.2), cysteine protease, CYP (Solyc02g076980.3), β-glucosidase 11 (Solyc01g081160.3), and cinnamyl alcohol dehydrogenase, CAD6-like (Solyc02g069250.3), were downregulated (***Supplementary** ***Figure 40b****). The qPCR results showed strong agreement with the RNA-seq data, thereby confirming the robustness and reliability of the transcriptomic dataset (***Supplementary** ***Figure 40c, d****). Given this validation, we next focused on key signaling pathways associated with plant immunity. Calcium signaling represents an early and central component of plant defense responses. Upon pathogen perception, transient cytosolic* Ca^{2+} *elevations are decoded by diverse* Ca^{2+} *sensors, including Calmodulin (CaM)/calmodulin-like proteins (CMLs), calcineurin B-like proteins (CBLs), and calcium-dependent protein kinases (CDPKs), which subsequently regulate downstream defense processes.*[81] *Heatmap analysis revealed that hydrogel treatment induced coordinated yet differential transcriptional changes in calcium signaling-related genes. Specifically, calmodulin-like protein 3 (Solyc06g073245.1) and CBP3 (Solyc06g068960.1) were upregulated, and two leucine-rich repeat (LRR) receptor-like kinase-related genes (Solyc02g072393.1 and Solyc02g068830.2) also showed relatively high expression levels. In contrast, CDPK20-like (Solyc01g006730.3) and CDPK17-like (Solyc12g099790.2) (were generally downregulated. This coexistence of upregulated and downregulated components suggests a selective reprogramming of the*

Ca^{2+} signaling network rather than a uniform activation or suppression, which is consistent with the dynamic and functionally specialized roles of different Ca^{2+} sensors in plant immunity.[82] *In parallel, ethylene signaling, an essential regulator of plant immune responses, was also significantly affected. Heatmap analysis indicated that several ethylene signaling- and defense-related genes were upregulated following hydrogel treatment. Notably, ETR4 (Solyc06g053710.3), ERF1 (Solyc05g051200.1), endochitinase 4-like (Solyc10g055780.1), and endochitinase 3-like (Solyc10g055820.2) exhibited pronounced induction. Importantly, the magnitude of induction was generally higher in the CTA DN gel-treated group compared to the CMA DN gel-treated group, suggesting a stronger activation of the ethylene-associated defense pathway. Previous studies have demonstrated that ethylene signaling enhances tomato resistance to bacterial wilt by activating transcription factors and sustaining pathogenesis-related gene expression.*[83] *Collectively, these results indicate that the enhanced disease control efficacy of CTA DN gel relative to CMA DN gel may be, at least in part, attributed to its stronger stimulation of ethylene-mediated defense responses.*"

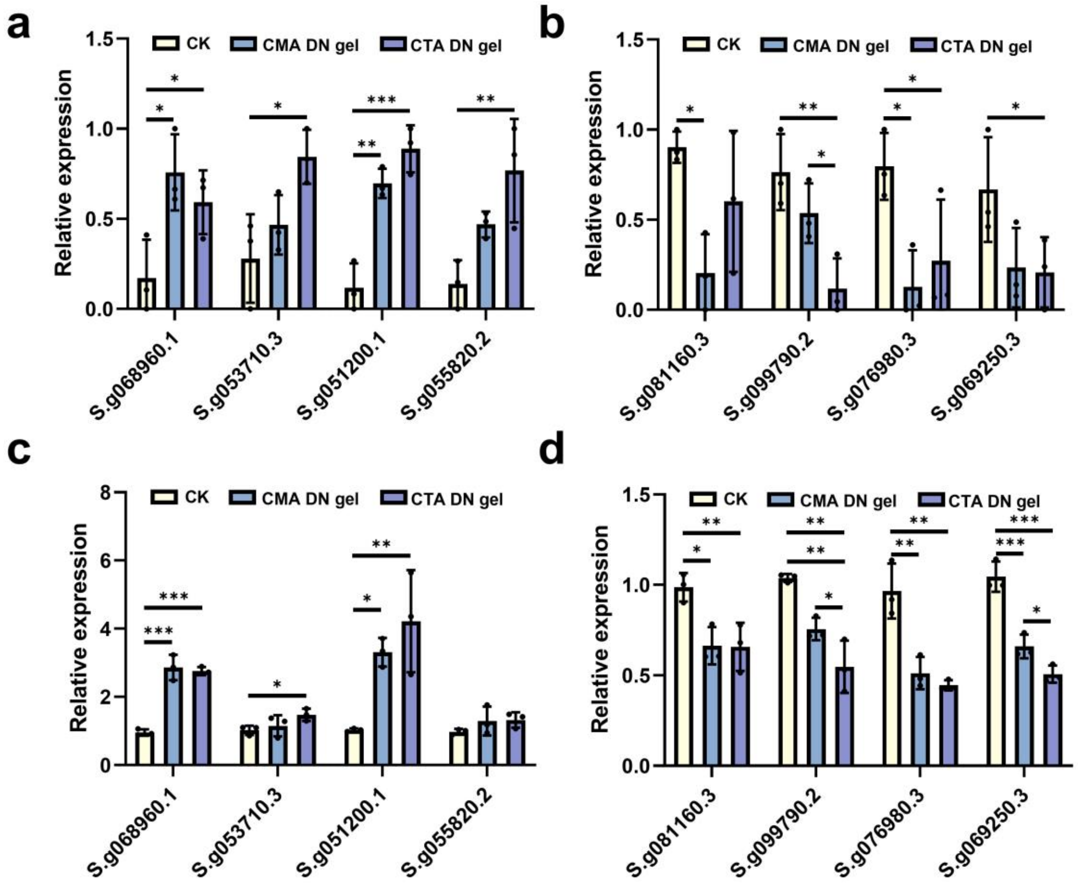

Supplementary Figure 40: Heatmap of transcriptomic analysis: **(a)** upregulated genes and **(b)** downregulated genes. qPCR validation of transcriptomic results: **(c)** upregulated genes and **(d)** downregulated genes. The asterisks indicate a significant difference according to one-way ANOVA with post hoc test (*$P < 0.05$, **$P < 0.01$, ***$P < 0.001$). The vertical bars indicate standard deviations.

Reviewer #3:

General comment: I co-reviewed this manuscript with one of the reviewers who provided the listed reports. This is part of the Nature Communications initiative to facilitate training in peer review and to provide appropriate recognition for Early Career Researchers who co-review manuscripts.

Response: We thank the reviewer for the valuable comments. We have carefully addressed all concerns and performed additional experiments. Please refer to the detailed responses provided above.

Point-to-point responses to the Editorial Office:

Editorial Comments:

Comment 1. First, we ask you to revise your paper to address our editorial requests (in the attached Author Checklist) and any remaining comments from reviewers (included at the end of this email, if applicable).
Response: We have completed the required reporting checklist in accordance with the guidelines and uploaded it as requested. In addition, we have carefully reviewed the reporting requirements for chemical and biomolecular material characterization and have revised the manuscript where necessary to ensure that all material characterization complies with these standards.

Point-to-point responses to the Reviewers:

Reviewer #1:
General comment: All the comments have been carefully addressed. Accept this as it is.
Response: We sincerely appreciate your time and consideration.

Reviewer #2:
General comment: The authors have carefully and comprehensively addressed the comments raised in the first round of review. The revisions are thorough and well justified, and the overall quality of the manuscript has been significantly improved. The additional analyses, clarifications, and improved presentation have strengthened the scientific rigor and clarity of the work.
Response: We sincerely thank the reviewer for the positive and encouraging evaluation of our work. We greatly appreciate the time and effort invested in reviewing our manuscript, and we are grateful for the constructive feedback that has helped improve its quality.

Reviewer #3:
General comment: I co-reviewed this manuscript with one of the reviewers who provided the listed reports. This is part of the Nature Communications initiative to facilitate training in peer review and to provide appropriate recognition for Early Career Researchers who co-review manuscripts.

Response: We sincerely appreciate the reviewer's contribution and support in the peer review process.
